# Trace element-dictated exosome modules and self-adaptive dual-network hydrogel orchestrate diabetic foot regeneration through complement-mitochondria-autophagy circuitry

**DOI:** 10.1186/s40779-025-00658-4

**Published:** 2025-10-28

**Authors:** Shuang-Qing Wang, Ming-Ji Jin, Ze-Ke Guo, Dong-Ri Shen, Li-Na Jin, Fang Cheng, Yan-Ru Zhao, Teng Liu, Yu-Cai Li, Nuo-Ya Wang, Ling-Qing Chen, Wei Huang, Xiu-Quan Quan, Zhong-Gao Gao

**Affiliations:** 1 State Key Laboratory of Bioactive Substance and Function of Natural Medicines, Institute of Materia Medica, Chinese Academy of Medical Sciences and Peking Union Medical College, Beijing, 100050 China; 2 Beijing Key Laboratory of Drug Delivery Technology and Novel Formulations, Department of Pharmaceutics, Institute of Materia Medica, Chinese Academy of Medical Sciences and Peking Union Medical College, Beijing, 100050 China; 3 Langfang Kangbao Huitai Biotechnology Co. Ltd, Langfang, 065001 Hebei China; 4 Lan-Jayeon Medical Device Co., Ltd, Beijing, 100050 China; 5 Emergency Department, Yanbian University Hospital, Yanji, Jilin, 133000 China

**Keywords:** Exosomes (Exo), Hydrogel, Trace element, Diabetic foot ulcers (DFU), Complement 1q binding protein (C1QBP)

## Abstract

**Background:**

Diabetic foot ulcers (DFU), perpetually trapped in a vicious cycle of inflammation and ischemia, remain a significant clinical challenge. Exosomes (Exo) therapy holds promise for tissue repair, yet its functional potency and delivery efficiency are often limited.

**Methods:**

We proposed an integrated strategy combining trace elements (TE) programming, Exo engineering, and intelligent delivery to overcome both functional and delivery constraints. Multiple TE (Fe, Mg, Zn, Mn, and Se) were incorporated into a three-dimensional (3D) dynamic culture system to construct high-activity engineered Exo (3D-TE-Exo). The biological mechanisms were explored via transcriptomics, mitochondrial function assays, and oxidative stress analyses. A dual-network hydrogel, incorporating dynamic Schiff base bonds and ultraviolet (UV)-triggered disulfide bond reorganization, was developed for precise and sustained Exo release in vivo.

**Results:**

3D-TE-Exo achieved a yield of 1.9 × 10^12^ particles/ml, representing a 29-fold increase over conventional culture (6.5 × 10^10^ particles/ml). These Exo modulated the complement pathway, restored mitochondrial membrane potential, enhanced adenosine triphosphate (ATP) production, and activated autophagy, thereby alleviating oxidative stress, with complement 1q binding protein (C1QBP) identified as a key mediator. The hydrogel enabled prolonged Exo retention and controlled release at the wound site. In DFU rat models, this system achieved 89.71% wound closure by day 14, significantly higher than the 50.64% observed in controls.

**Conclusions:**

This study presents a synergistic approach integrating engineered Exo and smart biomaterials to accelerate DFU healing. The platform offers a multi-target intervention strategy with strong translational potential for the clinical management of chronic wounds.

**Supplementary Information:**

The online version contains supplementary material available at 10.1186/s40779-025-00658-4.

## Background

Diabetes is a major global public health challenge, and its prevalence is projected to increase from 529 million in 2021 to 1.31 billion in 2050 [1]. Diabetic foot ulcers (DFU), a devastating complication, affecting over 30% of the 537 million patients with diabetes worldwide [2], culminate in limb amputation every 30 s [3, 4]. With the expected rise in diabetes, DFU will impose an even bigger burden on health systems worldwide and may become one of the most costly complications of the disease [5, 6]. At the core of this crisis lies a pathogenic triad [7]: nutritional deficiency, macrophage paralysis trapped in proinflammatory states, and vascular deserts [8]. The current therapeutic approaches include debridement, off-loading, antibiotic cocktails, and wound dressings. However, more than 40% of ulcers recur within a year [9], whereas biofilm-armed superbugs evade most antibiotics, thus highlighting the ineffectiveness of symptom-centric approaches. Regenerative attempts using stem cells face the challenges of limited cell survival and reduced cell adaptation at the wound site [10]. This therapeutic context demands an innovative solution and a unified strategy to quell inflammation, rejuvenate cellular energy production, and stimulate angiogenesis, while overcoming hostile wound environments.

This challenge in cell therapy has spurred the development of cell-free therapeutic strategies. Exosomes (Exo), which are central mediators of the paracrine effects of stem cells, have attracted significant attention because of their low immunogenicity and non-tumorigenicity. These nanoscale carriers laden with miRNAs, enzymes, and growth factors have offered promise for precision therapeutics capable of remodeling the DFU microenvironment [11]. Exo reflect the biological effects of their parental cells [12]. Studies have shown that Exo derived from mesenchymal stem cells (MSCs) can suppress M1 macrophage polarization, restore endothelial connectivity, and replenish the redox balance [13], thereby exhibiting superior efficacy in promoting wound healing [14]. Yet no Exo-based formulations have yet been approved for clinical trials. Multiple barriers hinder the development of Exo therapeutics, including production limitations, weak biological activity, loss of integrity, short retention time, and low bioavailability [15]. Factors such as culture environment, isolation protocols, and purification methods play critical roles [16]. Exo derived from two-dimensional (2D) planar culture of MSCs has a low production and faces challenges in large-scale production, hindering clinical translation [17]. In contrast, three-dimensional (3D) culture more accurately simulates cell-cell and cell-substrate interactions and is superior to conventional 2D platforms in terms of Exo production [18]. Microcarrier-based cultures in bioreactors demonstrate excellent scalability for the expansion of both primary and pluripotent stem cells. The substantially increased surface area of these microcarriers enabled enhanced cell proliferation, achieving high productions within a clinically relevant timeframe [19]. Various research strategies have been developed to enhance the functionality of Exo, including modifications to MSCs culture conditions [20, 21].

Trace elements (TE) are essential for maintaining optimal human health, and play critical roles in various physiological processes, including growth, development, and metabolic functions [22]. Nutritional deficiencies are among the major risk factors for the development and healing of DFU, as patients with DFU exhibit lower levels of magnesium (Mg), zinc (Zn), iron (Fe), and selenium (Se) than healthy controls [23–25]. In addition, manganese (Mn) has also been widely utilized to accelerate diabetic wound healing [26, 27]. In recent years, the modulation of Exo secretion and activity through the TE preconditioning of cells has emerged as a research hotspot [28, 29]. For instance, Sr-preconditioned MSCs enhanced the yield of MSC-derived Exo and demonstrated superior therapeutic effects by alleviating chondrocyte iron-induced apoptosis and mitigating osteoclast-mediated joint pain [30]. Mo-inspired macrophage-derived exosomes (Mo-Exo) promoted the proliferation, migration, and angiogenic capacity of human umbilical vein endothelial cells (HUVECs) [31]. Although many studies have explored the relationships between various TE and Exo, it is important to recognize that the human body is a complex and integrated organism. The current research is primarily limited to the effects of individual TE. The physiological and biochemical functions of TE are multifaceted, and the impact of a single element may not fully reflect the true conditions of the body. The pharmacological interactions between multiple TE and diseases are often underestimated. Previously, we developed a solution containing multiple TE for the treatment of rheumatoid arthritis [32] and gastric ulcers [33], which demonstrated excellent immune modulation and tissue repair capabilities.

Even if these Exo reach the wound, their therapeutic windows are narrow. Most topically applied Exo disappear within a short time and are victims of neutrophil phagocytosis or matrix metalloproteinase digestion [34]. The low retention and instability of Exo in vivo pose significant obstacles to their therapeutic efficacy. Current delivery platforms, while improving retention, face the trilemma of compromising controlled release, mechanical stability, and microenvironment responsiveness [35]. Hydrogels are 3D hydrophilic networks with high biocompatibility, biodegradability, and mechanical tunability. Hydrogels are particularly suitable as wound dressings because of their ability to conform to irregular wound shapes, provide a moist healing environment, and facilitate oxygen (O_2_) exchange and nutrient transport [36]. The use of hydrogels to deliver Exo and accelerate wound healing has been widely reported [37, 38]. However, hydrogels that combine stimuli-responsive release and excellent mechanical properties are less common. Double-network hydrogels formed by reversible interactions (such as hydrogen bonds, disulfide bonds, imine bonds, and phenylboronic ester bonds) can self-heal while maintaining network integrity and mechanical stability, thereby meeting the requirements for long-term use [39, 40]. Hyaluronic acid (HA) is a polysaccharide that is abundant in the human body and known for its excellent biocompatibility [41]. Chitosan (CS), a natural polymer derived from chitin, is attracting increasing attention as a promising biomaterial for various biomedical applications [42]. Moreover, HA and CS accelerate tissue repair and promote wound contraction, making them promising for advanced wound dressings [43]. Fabricating HA/CS-based hydrogels that are easily loaded with drugs or bioactive substances and feature stimulus-responsive release in complex microenvironments could significantly expedite commercialization [44].

Given the prevalent deficiencies of multiple TE in patients with DFU and their critical roles in cellular metabolism, inflammation resolution, and tissue repair, there is a compelling need for strategies that can both replenish these elements and harness their biological benefits. We hypothesized that targeted TE supplementation could comprehensively meet the growth requirements of MSCs, thereby enhancing the secretion of Exo with superior therapeutic potential. Building on this rationale, the present study was designed to develop an integrated and intelligent DFU treatment approach by synergistically modulating Exo bioactivity through TE enrichment and achieving their precise, sustained delivery via a stimuli-responsive hydrogel platform.

## Materials and methods

### Materials

HA [H909939, molecular weight (1.5–2.5) × 10^6^ kD], streptozocin (STZ; S817944), phenol red (P816237), and 2,2-diphenyl-1-picrylhydrazyl (DPPH; D807297) were acquired from Shanghai Macklin Biochemical Co., Ltd. (Shanghai, China). CS (R013969, deacetylation degree ≥ 95%) was obtained from Rhawn (Shanghai, China). Lipoic acid (LA) was purchased from Shanghai Acmec Biochemical Technology Co., Ltd. (Shanghai, China). Bicinchoninic acid (BCA) protein assay kit (PC0020), 4',6-diamidino-2-phenylindole (DAPI) solution (ready-to-use) (C0065), radio immunoprecipitation assay (RIPA) buffer (high) (R0010), phosphate buffered saline (PBS; P1020), and matrigel basement membrane matrix (356234) were purchased from Beijing Solarbio Science & Technology Co., Ltd. (Beijing, China). N-hydroxysuccinimide (NHS; A54070) and Trizol (B46930) were purchased from Beijing InnoChem Science & Technology Co., Ltd. (Beijing, China). Mitochondrial membrane potential assay kit with JC-1 (C2006), crystal violet staining solution (C0121), calcein AM cell viability assay kit (C2013M), quickblock™ blocking buffer for immunol staining (P0260), 4% paraformaldehyde (P0099), immunostaining permeabilization buffer with Triton X-100 (P0096), and protease and phosphatase inhibitor cocktail for general use (P1046) were purchased from Shanghai Beyotime Biotech. Inc. (Shanghai, China). Cell counting kit-8 (CCK-8, CK001) and reactive oxygen species (ROS) assay kit (O040) were obtained from LABLEAD Inc. (Beijing, China). Ethanol (1170) and ethylene glycol were purchased from Beijing TongGuang Fine Chemicals Company (Beijing, China). Goat anti-rabbit IgG H&L (Alexa Fluor^®^ 488) (ab150077), goat anti-rabbit IgG H&L (Alexa Fluor^®^ 647) (ab150079), vascular endothelial growth factor (VEGF; ab32152), anti-CD86 antibody (ab239075), anti-mannose receptor antibody (CD206; ab64693), and anti-platelet endothelial cell adhesion molecule-1 antibody (CD31; ab281583) were purchased from Abcam (Shanghai, China). Sequestosome 1 (p62; GB11531), microtubule-associated protein light chain 3 (LC3; GB113801), and β-actin were purchased from Servicebio (Wuhan, China). Silent information regulator 1 (SIRT1; 13161–1-AP) and complement 1q binding protein (C1QBP) monoclonal antibody (1F9B1) were purchased from Proteintech Group, Inc. (Wuhan, China).

The mouse mononuclear macrophage leukemia cell line (RAW264.7), HUVECs, and human immortal keratinocyte line (HaCaT) cells were obtained from the Cell Resource Center, IBMS, CAMS/PUMC. All cells were cultured in Dulbecco’s modified Eagle’s medium (DMEM) supplemented with 10% fetal bovine serum (FBS) and 1% penicillin-streptomycin, and maintained in a humidified incubator at 37 °C in an atmosphere containing 5% CO_2_. The culture medium was replaced every 2 d, and cells were passaged when they reached 80% to 90% confluency to ensure optimal growth conditions.

Male SD rats (SPF, 6 weeks old, 160–180 g, *n* = 111) were purchased from Beijing Vital River Laboratory Animal Technology Co., Ltd., China [SCXK(jing)2021–0006]. All animal experiments were approved by the Laboratory Animal Ethics Committee in the Institute of Materia Medica and Peking Union Medical College (00004441). During the experiment, all procedures followed ethical standards.

### MSC isolation, culture, and characterization

Human umbilical cord MSCs (hUC-MSCs) were isolated from human umbilical cord tissue obtained from Yanbian University. This project has been approved by the Ethics Committee of Scientific Research/Cell Clinical Research of Yanbian University Hospital (20250022). Briefly, the tissue was digested in 0.075% collagenase type I prepared in PBS containing 2% penicillin/streptomycin for 30 min at 37 °C, 5% CO_2_. The resulting mixture was centrifuged at 2000 r/min for 5 min, and the cell pellet was resuspended in DMEM supplemented with 20% FBS, 1% L-glutamine, and 1% penicillin/streptomycin. The cell suspension was then filtered through a cell strainer, and the cells were plated in tissue culture plates to allow adhesion for 48 h. Once the culture reached 80% confluence, the MSCs were subcultured for further expansion and characterization.

The isolated MSCs were characterized by flow cytometry. The cells were incubated with a panel of human MSC marker antibodies for 30 min at room temperature, followed by centrifugation for 3 min and 2 washes in PBS containing 3% bovine serum albumin. Next, the cells were incubated with a secondary antibody for 30 min in the dark, washed again, and then resuspended in flow cytometry staining buffer for analysis using FlowJo (v7.6). In addition, the osteogenic, chondrogenic, and adipogenic differentiation potentials of the MSCs were evaluated. MSCs were cultured in normal medium and TE-rich medium, respectively. Cells cultured in medium containing TE were designated TE-MSCs. CCK-8 test was used to identify the proliferation rate of MSCs and TE-MSCs.

In a culture flask, 10 ml of microcarriers, 20 million cells, and 100 ml of TE-supplemented medium were combined. The flask was placed on a magnetic stirrer set to intermittent stirring at 30 r/min for 10 min, followed by 0 r/min for 1 h. When the MSCs on the microcarriers had proliferated 8- to tenfold, all microcarriers were transferred to a larger culture vessel. Fresh microcarriers were added to further expand the culture in serum-containing medium, after which the medium was replaced with serum-free medium for an additional 2 d of culture. These MSCs are referred to as 3D-TE-MSCs. For comparison, MSCs were also cultured in a conventional 3D system without TE (following the same medium protocol: serum-containing for expansion, serum-free for the final 2 d), designated as 3D-MSCs.

### Exo extraction and identification

This study employed differential ultracentrifugation to isolate Exo from 3D cultured MSCs conditioned medium. The collected medium underwent sequential centrifugation at 4 °C: initial preprocessing at 300×*g* for 10 min to remove intact cells, followed by 2000 × *g* for 30 min to eliminate cellular debris and apoptotic bodies. The clarified supernatant was then subjected to 10,000 × *g* centrifugation for 30 min to pellet large protein aggregates. Following this, ultrafiltration purification was performed using a 0.22 μm membrane (Millipore Steritop) to exclude vesicles >220 nm and protein complexes. For Exo enrichment, ultracentrifugation at 100,000 × *g* for 70 min was conducted using an Optima MAX-XP ultracentrifuge (Beckman Coulter) under 4 °C. The resulting pellet was gently resuspended in chilled pH 7.4 PBS and subjected to repeated ultracentrifugation (100,000 × *g*, 70 min) to remove residual impurities. The final Exo preparation was resuspended in sterile PBS and aliquoted for storage at –80 °C. Two Exo groups were established based on their parental MSCs cultures: 3D-Exo (derived from 3D-MSCs cultured in standard medium) and 3D-TE-Exo (derived from 3D-TE-MSCs cultured in TE-supplemented medium).

Exo were comprehensively characterized through multimodal analytical approaches. The TE concentrations in 3D-Exo and 3D-TE-Exo were quantified using inductively coupled plasma mass spectrometry (ICP-MS). Morphological evaluation was conducted via transmission electron microscopy (TEM; Hitachi H-600, Japan), where Exo samples were fixed with 2% paraformaldehyde, deposited on carbon-coated grids, and negatively stained with uranyl acetate to visualize their cup-shaped nanostructure and bilayer membrane integrity. Protein quantification was performed using a commercial BCA assay kit, following the manufacturer's protocols. Nanoparticle tracking analysis (NTA; ZetaView, Particle Metrix, Germany) was conducted to determine particle size distribution (30–200 nm) and concentration under dynamic light scattering mode (laser wavelength: 488 nm; camera sensitivity: 11). Zeta potential and hydrodynamic diameter measurements of Exo were measured using a ZS90 Malvern Zeta Nanosizer, and Exo suspensions were diluted in PBS to avoid aggregation. Surface marker validation by Western blotting confirmed the enrichment of exosomal tetraspanins and Endosomal Sorting Complex Required for Transport-associated protein tumor susceptibility gene 101 (TSG101), while excluding endoplasmic reticulum marker calnexin to ensure purity. In the cellular uptake assays, Exo were fluorescently labeled with Paul Karl Horan 67 (PKH-67), incubated with HUVECs and HaCaT cells for 4 h, and internalization efficiency was quantified via confocal microscopy. This integrated workflow ensures rigorous validation of exosomal identity, functionality, and bioavailability for downstream applications.

### Inflammation and oxidative stress

#### ROS staining

HUVECs and HaCaT cells were seeded at a density of 1 × 10^5^ cells per well in a 12-well plate. The cells were induced with 200 μmol/L hydrogen peroxide (H_2_O_2_) for 24 h. Afterward, the cells were co-cultured with different formulations for 24 h. The cells were then treated with 10 μmol/L 2',7'-dichlorodihydrofluorescein diacetate (DCFH-DA) for 30 min. Before imaging, the cells were washed 3 times with pH = 7.4 PBS. Finally, ROS images were captured using a confocal laser scanning microscope (CLSM; Biotek, Winooski, VT, USA).

#### Mitochondrial membrane potential

HUVECs and HaCaT cells were seeded at a density of 1 × 10^5^ cells per well in a 12-well plate. The cells were induced with 200 μmol/L H_2_O_2_ for 24 h. Afterward, the cells were co-cultured with different formulations for 24 h. A 5 μg/ml JC-1 working solution was then added to the culture medium and mixed. The cell plates were incubated in the incubator for 30 min. The cells were washed 3 times with pre-cooled PBS to remove unbound probes. The mitochondrial membrane potential was observed using CLSM. In terms of emission wavelengths, red fluorescence was detected at 590 nm and green fluorescence at 530 nm. Healthy cells predominantly exhibited red fluorescence, while inflammatory cells primarily showed green fluorescence.

#### Inflammatory cytokine and antioxidant enzyme assay

The concentrations of cytokines produced by the cells were measured to evaluate the anti-inflammatory and antioxidant activity of the formulations. First, HUVECs and HaCaT cells were pre-incubated with 200 μmol/L H_2_O_2_ for 24 h to establish an inflammation model. Then, different formulations were added, and the cells were further incubated for 24 h. Enzyme-linked immunosorbent assay (ELISA) was used to measure the levels of inflammatory cytokines [tumour necrosis factor (TNF)-α, interleukin (IL)-1β, IL-6, and IL-10] (Elabscience Biotechnology Co. Ltd., Wuhan, China) secreted by HUVECs and HaCaT cells using relevant kits. Meanwhile, the activity of superoxide dismutase (SOD) (NJJCBIO, Nanjing, China), the level of glutathione (GSH), and the content of malondialdehyde (MDA) in the supernatants of HUVECs and HaCaT cells were determined using appropriate kits.

#### Western blotting

HUVECs and HaCaT cells were seeded at a density of 2 × 10^5^ cells per well in a 6-well plate and treated with 200 μmol/L H_2_O_2_ for 24 h. The cells were then co-cultured with OLUE [ultraviolet (UV) light-irradiated oxidized hyaluronic acid (OHA) and lipoic acid-grafted chitosan (LACS) constructed hydrogel for Exo] for an additional 24 h. Cells were lysed using RIPA buffer supplemented with 1 mmol/L phenylmethanesulfonyl fluoride. After centrifuging the lysates at 12,000×*g* for 10 min at 4 °C, the supernatants were collected, and protein concentrations were measured using a BCA assay. The samples were separated by SDS-PAGE (Dakewe, Shenzhen, China) and transferred onto PVDF membranes (0.45 μm, Servicebio, China, WGPVDF45). Membranes were blocked with 5% nonfat milk in TBST for 1.5 h at room temperature, then incubated overnight with primary antibodies (1:1000) against p62, LC3-I/II, SIRT1, and β-actin at 4 °C. After washing 3 times, the membranes were incubated with HRP-conjugated secondary antibodies (1:3000) for 1.5 h at room temperature. Immunoreactive bands were detected using an ECL detection kit.

#### TEM

HaCaT cells were seeded into 6-well plates and induced to establish an inflammatory model. The cells were then incubated with OLUE for 24 h. After incubation, the HaCaT cells were collected into new centrifuge tubes and centrifuged at 3000 r/min for 10 min. The supernatant was discarded, and the cells were fixed with 2.5% glutaraldehyde for 24 h. The morphology of autophagic vesicles was then observed using a Bio-TEM.

### Repair of DFU

#### Model establishment

A type I diabetes model in rats was established by intraperitoneal injection of 60 mg/kg STZ (dissolved in citrate buffer, pH = 4.5) into SD rats. The model was considered successful when the fasting blood glucose (Glu) remained above 16.7 mmol/L for 1 week. Subsequently, under anesthesia, a full-thickness wound with a diameter of 4 mm was created on the dorsal aspect of the rat’s foot. The rats were randomly assigned to the following groups: a model group (no treatment, *n* = 6), a 3D-Exo group (0.5 ml, *n* = 6), a 3D-TE-Exo group (0.5 ml, *n* = 6), an OLUE hydrogel group (0.5 ml, *n* = 6), and a positive control group (Tegaderm™, *n* = 6). In addition, 6 healthy SD rats without any model establishment were included as an independent healthy control group. Wound healing was evaluated by capturing optical images at predetermined time points and calculating the wound closure rate using ImageJ software, while body weight was continuously recorded to assess the impact of the various treatments on diabetic foot wound healing.

#### Histopathology and blood chemistry analysis

Histological evaluation and analysis of inflammatory cytokines in wound tissues were conducted to assess the degree of repair and the progression of wound healing. The collected wound tissues were divided into several portions: one portion was fixed in 4% paraformaldehyde for histopathological analysis, including hematoxylin-eosin (H&E) staining, Masson staining, Sirius red staining, immunofluorescence staining, and immunohistochemical staining; another portion was homogenized for the detection of inflammatory cytokine concentrations and the expression of related genes using ELISA and reverse transcription-polymerase chain reaction (RT-PCR); and a third portion was stored on ice for ROS staining. H&E staining was used to evaluate epithelial regeneration, while Masson and Sirius red staining were employed to assess collagen deposition in the skin. ELISA was utilized to measure the levels of TNF-α, IL-1β, SOD, GSH, MDA, and transforming growth factor (TGF)-β (Hengyuan Biological Technology, Shanghai, China). RT-PCR was performed to quantitatively assess the expression of inflammation-related genes, such as *Tnf-α*, *Il-1β*, arginase-1 (*Arg-1*), and *Il-10*, (primer sequences are provided in Additional file 1: Table S1). Additionally, to further evaluate the efficacy of the hydrogel in promoting wound healing, immunofluorescence staining for CD31, VEGF, and α-smooth muscle actin (α-SMA) was performed to assess angiogenesis in the wound site, while fluorescent staining for CD86 and CD206 was used to observe macrophage phenotypes. Kiel-67 (Ki-67) immunohistochemical staining was employed to evaluate cellular proliferation, and immunofluorescence staining for CD11c and cluster of differentiation (CD3) was used to examine changes in immune cell populations at the wound site.

Other detailed methods for material synthesis and characterization, as well as in vitro and in vivo experiments, are provided in the Additional file 1: Methods.

### Statistical analysis

All data are presented as mean ± standard deviation (SD). Statistical analysis was performed using GraphPad Prism 9.5 (GraphPad Software, Inc., La Jolla, CA, USA). Comparisons of two groups utilized the Student’s *t*-test. When comparing 3 or more groups, one-way analysis of variance (ANOVA) was employed, followed by Tukey’s post hoc test. Nonparametric tests were used for comparisons involving non-normal distributions. A *P*-value < 0.05 was considered statistically significant.

## Results

### Extraction and identification of Exo

**Fig. 1:**
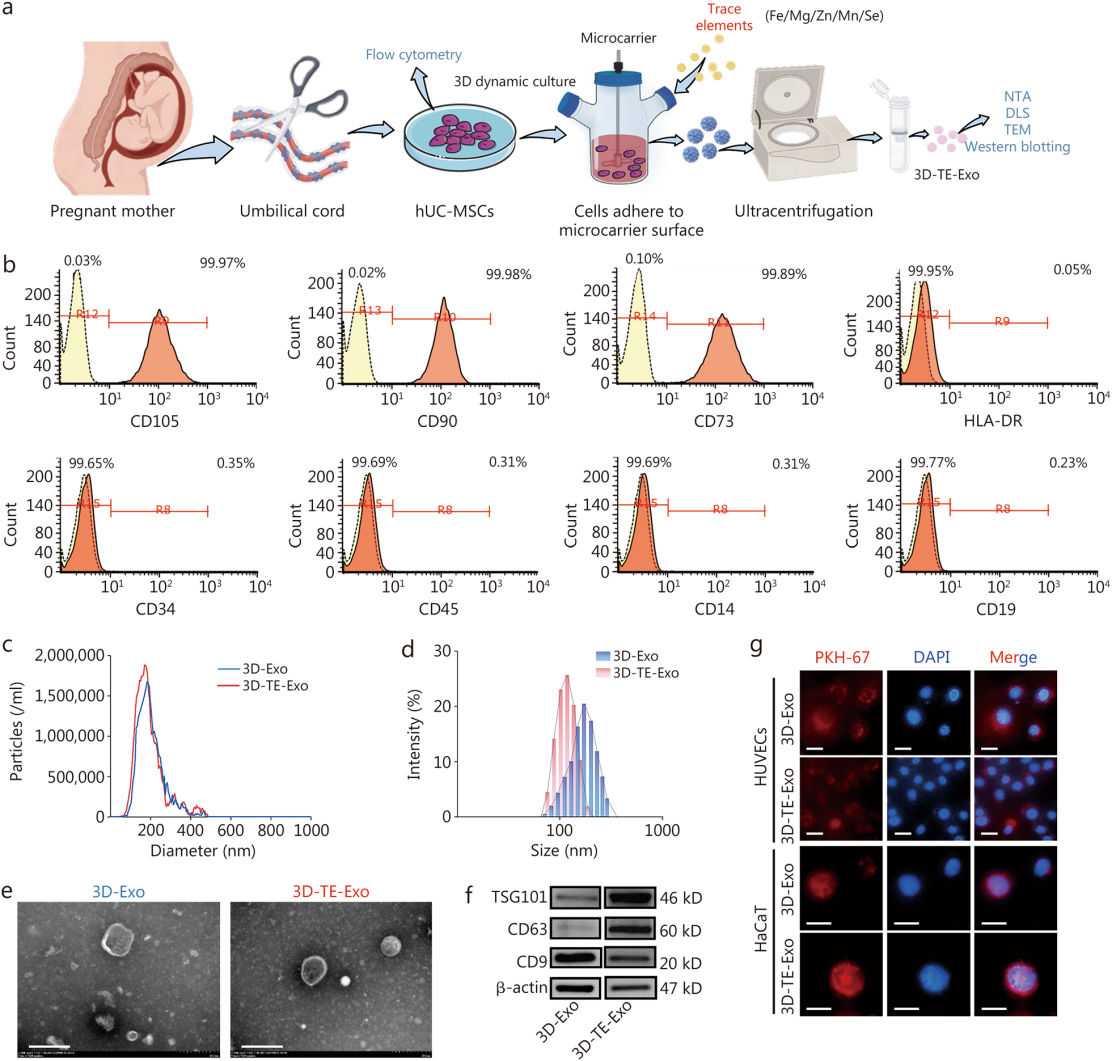
Characterization of 3D-Exo and 3D-TE-Exo and their intense uptake by HUVECs and HaCaT cells. **a** Schematic diagram of the extraction and detection of 3D-TE-Exo. **b** Flow cytometry analysis of cell surface markers on hUC-MSCs. **c** Size distribution profile of 3D-Exo and 3D-TE-Exo detected by NTA. **d** Size distribution profile of 3D-Exo and 3D-TE-Exo detected by DLS. **e** Morphology of 3D-Exo and 3D-TE-Exo was examined by TEM (scale bar = 200 nm). **f** Western blotting analysis for exosomal marker proteins TSG101, CD63, and CD9. **g** Representative image of 3D-Exo and 3D-TE-Exo uptake by HUVECs and HaCaT cells (scale bar = 20 μm). 3D three-dimensional, 3D-TE-Exo exosome derived from trace element-supplemented medium, 3D-Exo exosome derived from standard medium, hUC-MSCs human umbilical cord mesenchymal stem cells, NTA nanoparticle tracking analysis, DLS dynamic light scattering, TEM transmission electron microscopy, HLA-DR human leukocyte antigen-DR, CD cluster of differentiation, HLA-DR human leukocyte antigen d-related, TSG101 tumor susceptibility gene 101, HUVECs human umbilical vein endothelial cells, HaCaT human immortal keratinocyte line, Exo Exosomes, PKH-67 paul karl horan 67, DAPI 4',6-diamidino-2-phenylindole

The extraction and characterization process of 3D-TE-Exo is illustrated in Fig. 1a. The TE-enriched culture medium contains various elements, including Fe, Mg, Zn, Mn, and Se, with specific concentrations detailed in Additional file 1: Table S2. For comparison, Exo isolated from culture medium without TE supplementation is referred to as 3D-Exo. Initially, MSCs were isolated and cultured under 3D conditions to promote the formation of cellular spheroids. In accordance with the International Society for Cellular Therapy’s position statement, flow cytometric analysis confirmed that the MSCs exhibited high expression of surface markers CD105, CD90, and CD73, while lacking the expression of hematopoietic and immune markers human leukocyte antigen d-related (HLA-DR), CD34, CD45, CD14, and CD19 (Fig. 1b). To confirm the multipotency of the MSCs, trilineage differentiation assays were performed, demonstrating their capacity for osteogenic, chondrogenic, and adipogenic differentiation (Additional file 1: Fig. S1a). Compared with conventional medium, the TE-supplemented medium promoted MSCs proliferation by 115% to 120% (Additional file 1: Fig. S1b). Notably, the addition of TE during the 3D culture process markedly altered the cellular microenvironment, characterized by reduced cytoskeletal tension, upregulation of multiple gene expression pathways, and modulation of differentiation trajectories [16]. Exo were subsequently isolated via differential ultracentrifugation [45]. Their elemental composition was quantitatively analyzed using ICP-MS. The results revealed that the concentrations of Fe, Mg, Zn, Mn, and Se in 3D-TE-Exo were 2.36, 1.90, 0.08, 0.12, and 0.27 μg/mg, respectively, whereas the corresponding concentrations in 3D-Exo were 0, 1.01, 0, 0.05, and 0.22 μg/mg. These values indicate that all 5 elements were significantly elevated in 3D-TE-Exo compared to 3D-Exo, confirming successful TE enrichment within the exosomal cargo. A key mechanism by which TE exerts its biological functions involves its interaction with the cell membrane or internalization via endocytosis, followed by intracellular release that influences gene expression and cellular signaling pathways [46]. Based on NTA and BCA results, the particle number of 3D-TE-Exo (1.9 × 10^12^ particles/ml) was significantly higher than that of 3D-Exo (6.5 × 10^10^ particles/ml) (Fig. 1c). Both Exo types exhibited a typical size distribution ranging from 30–150 nm (Fig. 1d), possessed a negative surface zeta potential (Additional file 1: Fig. S2), and displayed characteristic cup-shaped and disc-like morphologies (Fig. 1e). Furthermore, Western blotting analysis confirmed the presence of canonical exosomal markers, including TSG101, CD63, and CD9 (Fig. 1f), in both 3D-Exo and 3D-TE-Exo, in agreement with previously published literature [47]. The cellular uptake studies indicated that both 3D-Exo and 3D-TE-Exo were efficiently internalized by HUVECs and HaCaT cells within 6 h, with no statistically significant difference in uptake efficiency (Fig. 1g).

### Preparation and characterization of polymeric materials

#### Synthesis and characterization of OHA

The ring-opening reaction of HA using NaIO_4_ is a well-established method (Additional file 1: Fig. S3a) for synthesizing OHA. The stretching vibration peaks of the hydroxyl (-OH) groups of HA and OHA were observed at 3262 and 3269 cm^−1^, respectively (Additional file 1: Fig. S3b). Notably, a new absorption peak appeared at 1739.37 cm^−1^ in the OHA spectrum, corresponding to the stretching vibration of aldehyde groups, confirming successful oxidation. In the nuclear magnetic resonance hydrogen spectroscopy (^1^H NMR) spectrum of OHA, the solvent peak of D_2_O was observed at 4.78 ppm, while newly emerged peaks at 4.83, 4.99, and 5.07 ppm were attributed to the hemiacetal protons formed through intramolecular interactions between aldehyde groups and adjacent hydroxyl groups (Additional file 1: Fig. S3c). Using the hydroxylamine hydrochloride titration method, the degree of oxidation of OHA was calculated to be 20.8%. Collectively, these results confirm that NaIO_4_ effectively oxidizes HA to generate OHA with reactive aldehyde functionalities. The process is straightforward, highly reproducible, and amenable to downstream purification, making it well-suited for scalable industrial production.

#### Synthesis and characterization of LACS

LA was covalently grafted onto CS via an ethyldimethylaminopropyl carbodiimide (EDC)/NHS-mediated amidation reaction (Additional file 1: Fig. S4a), which significantly improved the water solubility of CS and expanded its application potential. The Fourier transform infrared (FT-IR) spectrum of LACS (Additional file 1: Fig. S4b) exhibited characteristic peaks at 1556.27 and 1634.86 cm^−1^, corresponding to the in-plane bending vibrations of amide groups, confirming the successful formation of amide bonds between CS and LA. Additionally, a distinct peak at 1548.56 cm^−1^ was attributed to C–N stretching vibrations. A notable absorption peak at 604.57 cm^−1^ was observed, which is assigned to the S–S bond in LACS. The ^1^H NMR spectrum revealed characteristic signals for CS backbone in the range of 2.3–2.7 ppm and for LACS at 3.5–3.9 ppm, corresponding to the C1-glucosamine ring and the glucopyranose ring, respectively (Additional file 1: Fig. S4c). Compared to CS, LACS exhibited new proton signals in the regions of 2.0–2.5 ppm and 3.0–3.5 ppm, which are attributed to methylene groups and thiol groups (S-CH_2_). The combined FT-IR and ^1^H NMR results unequivocally confirm the successful grafting of LA onto CS backbone. The amino-containing LACS possesses photo-crosslinkable properties. The mechanism involves the cleavage of disulfide bonds to generate free radicals, which subsequently re-polymerize to form new disulfide bonds, thereby establishing a crosslinked network. This dynamic process endows the material with tunable mechanical properties and stimuli-responsive behavior.

### Construction and evaluation of the hydrogel

#### Construction of the hydrogel

**Fig. 2:**
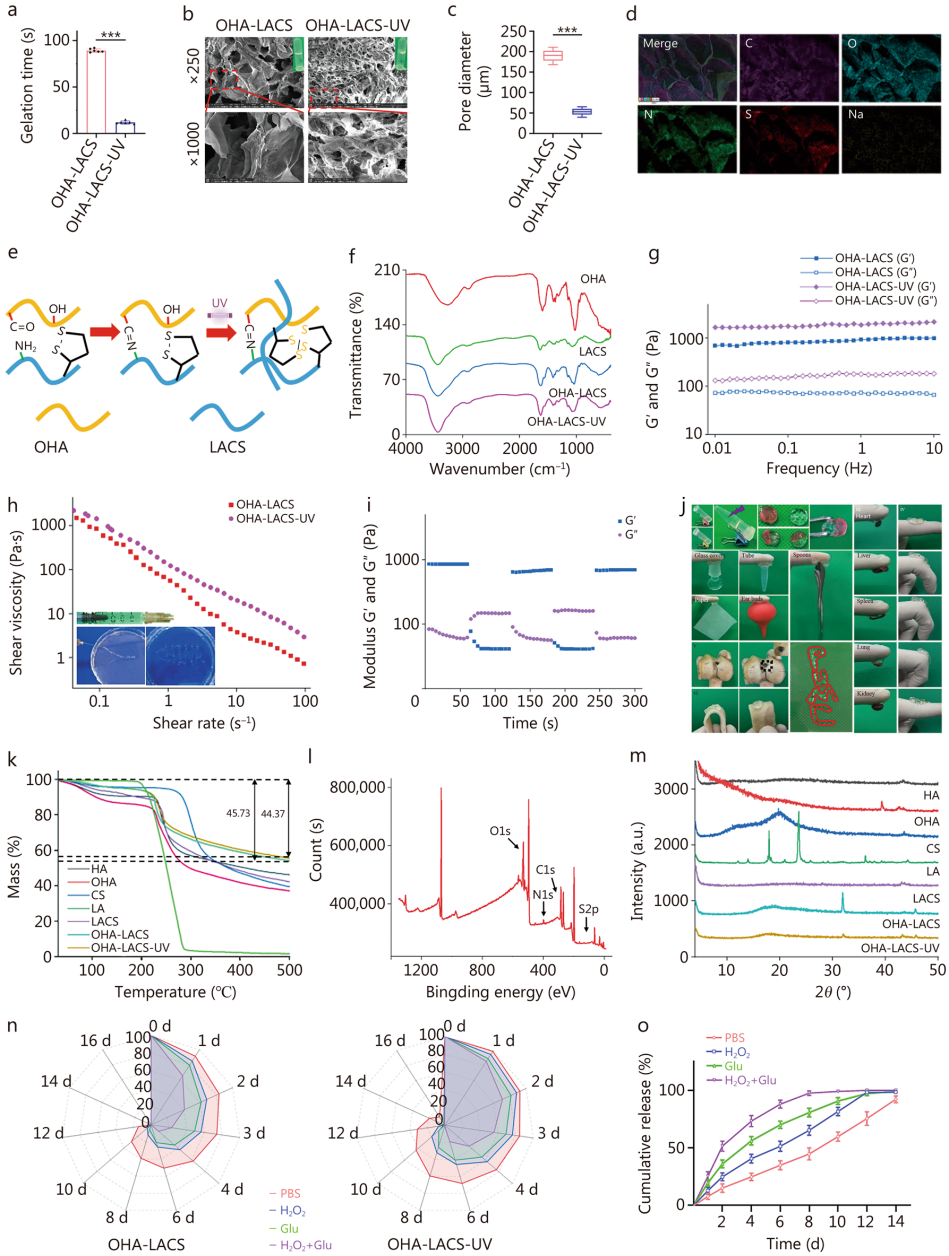
Physicochemical properties of OHA-LACS and OHA-LACS-UV hydrogels. **a** Gelation time of OHA-LACS and OHA-LACS-UV hydrogels (*n* = 6). **b** The representative SEM of OHA-LACS and OHA-LACS-UV hydrogels.** c** Pore diameter of OHA-LACS and OHA-LACS-UV hydrogels (*n* = 10). **d** The representative EDS elemental mapping of OHA-LACS-UV hydrogel, including C, O, N, S, and Na. **e** Schematic diagram of the gelation mechanism of the OHA-LACS-UV hydrogel. **f** The FT-IR spectra of OHA, LACS, OHA-LACS, and OHA-LACS-UV. **g** Frequency sweep test results for OHA-LACS and OHA-LACS-UV hydrogels at a fixed strain. **h** Viscosity test of OHA-LACS and OHA-LACS-UV hydrogels with various shear rates. **i** Continuous alternate strain sweep test results for OHA-LACS-UV hydrogel from 1–300%. **j** Macroscopic characteristics of hydrogels. i: Hydrogel formation; ii: Self-healing; iii: Adhesion; IV and V: stretchability; VI: injectability. **k** The TGA spectra of HA, OHA, CS, LA, LACS, OHA-LACS, and OHA-LACS-UV. **l** The XPS spectra of OHA-LACS hydrogel. **m** The XRD spectra of HA, OHA, CS, LA, LACS, OHA-LACS, and OHA-LACS-UV. **n** Degradation properties of OHA-LACS and OHA-LACS-UV hydrogels (*n* = 3). **o** In vitro cumulative release of 3D-TE-Exo from OLUE at different solutions (*n* = 3). ^***^P< 0.001. SEM scanning electron microscope, EDS energy dispersive spectrometer, FT-IR fourier transform infrared, TGA thermogravimetry analysis, XPS X-ray photoelectron spectroscopy, XRD X-ray diffraction, G′ storage modulus, G″ loss modulus, OHA oxidized hyaluronic acid, HA hyaluronic acid, CS chitosan, LA lipoic acid, LACS lipoic acid-grafted chitosan, OHA-LACS hydrogels constructed from oxidized hyaluronic acid and lipoic acid-grafted chitosan, OHA-LACS-UV hydrogels constructed from oxidized hyaluronic acid and lipoic acid-grafted chitosan by ultraviolet light, OLUE ultraviolet light (UV)-irradiated oxidized hyaluronic acid (OHA) and lipoic acid-grafted chitosan (LACS) constructed hydrogel for exosomes, Glu glucose, H_2_O_2_ hydrogen peroxide

The OHA-LACS hydrogel was prepared by mixing equal volumes of OHA and LACS. Gelation time is a critical parameter for biomedical applications. Using the vial inversion method, the gelation time of the OHA-LACS hydrogel was measured to be (89.17 ± 1.94) s. Notably, upon UV irradiation (365 nm, 10 mW/cm^2^) the gelation time was significantly reduced to (11.83 ± 1.72) s for the OHA-LACS-UV hydrogel (Fig. 2a). This rapid photo-induced crosslinking allows the hydrogel to quickly adhere to the wound surface, effectively preventing the loss of bioactive substances and providing immediate wound protection, thereby reducing patient discomfort and enhancing clinical operability. Scanning electron microscope (SEM) revealed that the OHA-LACS hydrogel exhibited a porous structure with relatively large and irregular pore sizes. In contrast, the OHA-LACS-UV hydrogel displayed a denser microarchitecture characterized by significantly reduced pore sizes and thickened pore walls (Fig. 2b), with an average pore size of (52.94 ± 8.27) μm (Fig. 2c). This more uniform porous structure not only enhances the control of Exo release but also improves the mechanical properties of the hydrogel. Meanwhile, it facilitates the absorption of wound exudate and promotes the homogeneous distribution of nutrients, thereby creating a favorable microenvironment for cell adhesion and tissue regeneration. Energy dispersive spectroscopy (EDS) analysis further confirmed the uniform distribution of elements such as C, O, N, S, and Na within the OHA-LACS-UV hydrogel (Fig. 2d), validating the compositional homogeneity and successful integration of all hydrogel components.

The formation mechanism of the hydrogel involves two primary crosslinking pathways (Fig. 2e). The first is the formation of dynamic Schiff base linkages, resulting from the condensation reaction between aldehyde groups on OHA and amino groups on LACS. The FT-IR spectrum of OHA-LACS revealed characteristic Schiff base vibrations in the range of 1586.16–1579.89 cm^−1^ (Fig. 2f). Additionally, a broad and intense absorption peak at 3426.89 cm^−1^ was observed, corresponding to extensive hydrogen bonding within the hydrogel network, which contributes to its structural integrity. Second, the second pathway involves UV-responsive disulfide bond dynamics. In the OHA-LACS-UV hydrogel, the hydrogen bonding peak exhibited a red-shift to 3440.87 cm^−1^, indicating the photolytic cleavage of disulfide bonds into free thiol groups. These thiol groups subsequently undergo thiol-thiol coupling or thiol-amine crosslinking with adjacent CS chains, resulting in the formation of an interpenetrating polymer network. Distinct from conventional light-triggered hydrogels, the OHA-LACS-UV system does not require the addition of photoinitiators for UV-induced crosslinking. This initiator-free strategy not only improves the biocompatibility of the hydrogel by avoiding potential cytotoxicity associated with residual initiators but also simplifies the preparation process and reduces manufacturing costs, thereby enhancing its translational potential for clinical applications.

#### Rheology

The rheological properties of the hydrogel were determined by its storage modulus (G′) and loss modulus (G″). G′ represents the ability of hydrogel to store elastic deformation energy and reflects its stiffness. For both the OHA-LACS and OHA-LACS-UV hydrogels, G′ consistently exceeded G″ across the entire frequency range tested (Fig. 2g), indicating a predominantly elastic, solid-like behavior typical of viscoelastic hydrogels. This also suggests that the hydrogel network maintained structural integrity under varying frequencies. Notably, the OHA-LACS-UV exhibited significantly higher G′ values compared to the OHA-LACS, demonstrating enhanced mechanical strength and robustness, which are advantageous for wound dressing applications. The injectability of the OHA-LACS was further evaluated via rheological measurements. As shown in Fig. 2h, the viscosities of the OHA-LACS and OHA-LACS-UV hydrogels decreased sharply with increasing shear rate before gradually stabilizing, confirming their shear-thinning behavior typical of a non-Newtonian pseudoplastic fluid, a property that facilitates their injectability. UV-induced intermolecular disulfide crosslinking links discrete molecular chains into a penetrating 3D network. The network produces strong elastic resistance and viscous dissipation to external forces. This increases the viscosity of the hydrogel. Given that skin is subject to constant and frequent motion, an ideal hydrogel should also exhibit excellent fatigue resistance. Oscillatory strain sweep tests at a fixed frequency of 1.0 Hz were conducted to record the G′ and G″ of the OHA-LACS-UV hydrogel, assessing its self-healing capacity (Fig. 2i). During the initial 60 s under low strain, G′ remained substantially higher than G″, confirming the mechanical stability of the hydrogel. Upon application of high strain, G″ transiently exceeded G′, indicating temporary network disruption and loss of structural integrity. Remarkably, when the strain was reduced to a low level, both G′ and G″ rapidly recovered to their initial values, demonstrating the hydrogel’s excellent self-healing ability. This dynamic recovery is attributed to the reversible nature of Schiff base linkages and disulfide bond exchange, which enable the rapid reconstruction of the polymer network. This self-healing property is particularly crucial for wound care applications, where the hydrogel must withstand repeated mechanical stress without compromising its functionality. Collectively, the rheological analysis confirms that the OHA-LACS-UV hydrogel possesses a desirable combination of elasticity, mechanical robustness, injectability, and self-healing capacity, making it highly suitable for dynamic wound healing environments.

#### Macroscopic characteristics of hydrogels

The superior physicochemical properties of the hydrogel are fundamental to its enhanced performance in biomedical applications. We systematically evaluated the macroscopic characteristics of the OHA-LACS-UV, including self-healing, adhesion, tensile resistance, and the injectability of the OHA-LACS hydrogel (Fig. 2j). When the two solutions, OHA and LACS, are mixed and then irradiated with UV light, a hydrogel is formed (Fig. 2ji). The results demonstrated that two separate pieces of cut OHA-LACS-UV rapidly re-adhered, confirming its robust self-healing capability (Fig. 2jii). The OHA-LACS-UV adhered firmly to various substrates such as glass covers, tubes, paper, ear buds, spoons, and various rat tissues (heart, liver, spleen, lung, and kidney) (Fig. 2jiii). This superior adhesion arises from the abundant presence of carboxyl, catechol, and amino groups, which engage in both covalent bonds and non-covalent interactions with tissue surfaces. This multi-modal adhesion is particularly beneficial for achieving rapid wound closure and stable fixation to irregular or dynamic tissue environments. Moreover, when applied to a finger and porcine skin, OHA-LACS-UV maintained its structural integrity under repeated bending, twisting, and stretching, and quickly recovered to its original shape without fracture (Fig. 2jIV, V). The injectability of the OHA-LACS was validated by injecting it into drilled bone cavities and using it to write “PUMC” (Fig. 2jVI). Following UV exposure, the hydrogel rapidly hardened, further enhancing its mechanical properties and enabling it to perfectly fill irregular wounds and tightly adhere to injured tissues. In summary, the hydrogel precursor is rapidly formed via a Schiff base reaction, followed by UV-induced disulfide bond reorganization that significantly enhances the mechanical strength of the OHA-LACS-UV hydrogel. The hydrogel exhibits a desirable combination of injectability, flexibility, tissue adhesion, tensile resistance, and self-healing capability.

#### Physicochemical properties of hydrogels

The structural and performance characteristics of the hydrogels were investigated via thermal stability experiments. Thermogravimetric analysis (TGA) (Fig. 2k) and differential thermogravimetry (DTG) (Additional file 1: Fig. S5a) were used to analyze the thermal stability of the samples. The results indicated that, owing to the long polymeric chains of OHA and LACS, both the OHA-LACS and OHA-LACS-UV exhibited good thermal stability up to 200 °C. As the temperature increased, a marked decline in the TGA curve was observed, primarily owing to water loss from the samples during heating. The TGA curve revealed that thermal degradation of the polymer occurred in 3 stages. The first stage, occurring at approximately 230 °C, is attributed to the evaporation of water. The second stage, between 230–400 °C, shows weight loss related to the cleavage of sugar rings, the expulsion of intermolecular water, and the degradation of macromolecular chains. The third stage, occurring between 400–500 °C, is mainly due to further degradation of the molecular chains. Both OHA and LACS possess inherently high thermal stabilities, which are further enhanced by hydrogel formation. During the constant weight loss process, the hydrogels continuously shed free and bound water, and OHA-LACS-UV exhibited superior thermal stability compared to OHA-LACS (Fig. 2k). The peaks appearing on the DTG curve correspond to the weight change stages on the TGA curve. Differential scanning calorimetry (DSC), commonly used to characterize polymer crystallinity, intramolecular and intermolecular interactions, miscibility, and homogeneity, showed that the characteristic peaks of OHA-LACS-UV remained nearly unchanged compared with those of OHA-LACS (Additional file 1: Fig. S5b). Furthermore, TGA, DTG, and DSC results demonstrated that the incorporation of LA enhanced the thermal stability of CS. X-ray photoelectron spectroscopy (XPS) was used to analyze the chemical composition and relative elemental content of the samples. The XPS spectrum of OHA-LACS-UV (Fig. 2l; Additional file 1: Fig. S6) revealed characteristic peaks corresponding to C, O, N, and S, which were mainly derived from the polymer and PBS, indicating that no additional impurities were present in the hydrogel. X-ray diffraction (XRD), which was used to elucidate the crystallinity of the materials, indicated that LACS did not exhibit the characteristic peaks of CS (Fig. 2m), suggesting that the LA modification disrupted the crystalline structure between the CS chains, thereby enhancing its solubility in water. In comparing OHA with LACS, changes in the diffraction peaks between 2*θ* = 30° –45° can be attributed to interactions between aldehyde and amino groups, while the enhancement of the characteristic peak at 43.19° in OHA-LACS-UV may indicate a rearrangement of the topological network within the hydrogel.

#### Swelling, degradation, and release

The degradation properties of hydrogels are a critical factor in tissue engineering, as they not only absorb wound exudate but also provide a moist environment conducive to tissue regeneration [48]. Our results showed that both OHA-LACS and OHA-LACS-UV hydrogels rapidly swelled in pH = 7.4 PBS (Additional file 1: Fig. S7). After 10 h, the swelling rate of OHA-LACS was (779.70 ± 21.34)%, while that of OHA-LACS-UV was (461.10 ± 16.62)%, indicating that the OHA-LACS-UV hydrogel exhibited a lower swelling rate, likely due to its smaller pore size, which restricts the ingress of water molecules. Excessive swelling may accelerate hydrogel degradation, thereby increasing the administration frequency. Considering the inflammatory and high-Glu microenvironment in diabetic wounds, we evaluated the degradation behavior of the hydrogels under conditions of pH = 7.4 PBS, 1 mmol/L H_2_O_2_, 3 mg/ml Glu, and a combination of 1 mmol/L H_2_O_2_ + 3 mg/ml Glu (H_2_O_2_ + Glu). The results demonstrated that in pH = 7.4 PBS, both OHA-LACS and OHA-LACS-UV degraded slowly, with residual weights at day 12 of (14.10 ± 1.94)% and (31.13 ± 3.76)% (w/w), respectively (Fig. 2n). However, in the presence of high levels of H_2_O_2_ and Glu, the degradation rate of the hydrogels was significantly accelerated. Notably, in the H_2_O_2_ + Glu condition, OHA-LACS was nearly completely degraded by day 6, exhibiting a degradation rate markedly higher than that at pH = 7.4 PBS, thereby demonstrating the significant responsiveness attributable to the sensitivity of the Schiff base to ROS and Glu. In contrast, OHA-LACS-UV also displayed pronounced responsiveness, retaining (19.17 ± 2.04)% of its weight on day 6. Secondary crosslinking induced by UV irradiation significantly enhanced the mechanical properties of the hydrogel, thereby delaying its degradation rate.

As an Exo carrier, the hydrogel achieves sustained release of Exo by controlling its degradation rate and enhancing its bioavailability. Based on the ROS and Glu responsiveness of the Schiff bases, we further investigated the release behavior of the OHA-LACS-UV hydrogel loaded with 3D-TE-Exo (OLUE). The incorporation of Exo did not significantly affect the gelation time or mechanical properties of the hydrogel. In pH = 7.4 PBS, the OLUE hydrogel released Exo gradually, with a cumulative release of (35.10 ± 3.94)% (w/w) by day 6 (Fig. 2o). Under conditions of H_2_O_2_ and Glu, the cumulative release of Exo from OLUE was (56.33 ± 3.66)% and (53.90 ± 4.39)%, respectively. Most importantly, under treatment with H_2_O_2_ + Glu, Exo were released rapidly, reaching a cumulative release of (88.10 ± 3.44)% by day 6 and complete release by day 12. This phenomenon is attributed to the rapid cleavage of Schiff bases and disulfide bonds under high ROS and Glu conditions, which causes the hydrogel to collapse and swiftly release Exo, exerting its anti-inflammatory, antioxidant, and tissue repair-promoting effects. Moreover, the cleavage of Schiff bases and disulfide bonds consumes ROS and Glu, which further modulates the wound microenvironment. To further elucidate the release properties of the hydrogels, we investigated their release kinetics. The regression coefficient (*R*^*2*^) was used to evaluate the goodness-of-fit of the release models. The in vitro release profile of OLUE hydrogels was best described by the first-order model (Additional file 1: Table S3). Their release behavior followed a non-Fickian diffusion mechanism [42], where both diffusion and polymer relaxation contributed to controlling drug release. Throughout the process, swelling and relaxation of the polymer chains, along with infiltration of the matrix polymers, play key roles.

### Biocompatibility

Different concentrations of Exo exert varying effects on the cells, making it crucial to determine the optimal concentration. First, the effects of different concentrations (0.10–2.00 μg/ml) of 3D-Exo and 3D-TE-Exo on the viability of HUVECs and HaCaT cells were evaluated using the CCK-8 assay. The results showed that as the concentration increased, both types of Exo promoted cell proliferation (Additional file 1: Fig. S8a), with the most pronounced proliferative effect observed at 2.00 μg/ml. On day 3, the viability of HUVECs reached (126.92 ± 5.54)% with 3D-Exo and (135.70 ± 8.79)% with 3D-TE-Exo, while HaCaT cell viability was (119.32 ± 10.58)% with 3D-Exo and (126.85 ± 8.61)% with 3D-TE-Exo. At the same concentration, 3D-TE-Exo exhibited a significantly greater proliferative effect than 3D-Exo, highlighting the advantages of TE. Subsequently, we evaluated the safety of OHA-LACS-UV at various concentrations (10–100 μg/ml), which did not significantly affect the viability of HUVECs and HaCaT cells (Additional file 1: Fig. S8b, c). Furthermore, upon assessing the effect of OLUE on cells, HUVECs and HaCaT cells were found to adhere well and exhibit robust growth (Additional file 1: Fig. S8d), indicating that OLUE promotes cell viability (Additional file 1: Fig. S8e). 5-Ethynyl-2'-deoxyuridine (EdU) staining was performed to further investigate the mechanism underlying OLUE-induced cell proliferation. EdU staining, which accurately reflects cell proliferation rate and cell cycle status under non-damaging conditions, revealed that cell proliferation was markedly inhibited in the model group [49]. Compared with the control group, these results were consistent with those of the CCK-8 and live/dead assays. In contrast, the number of EdU-positive cells increased upon treatment with the formulations (Additional file 1: Fig. S8f), indicating that 3D-TE-Exo promotes DNA replication and cell proliferation, further confirming the excellent biocompatibility of the hydrogel. Finally, the hemolysis rate of the OLUE was below 5% (Additional file 1: Fig. S9), in accordance with the international standards for biomaterials. In summary, the results of the CCK-8 assay, live/dead staining, EdU staining, and hemolysis tests collectively demonstrated that the hydrogel exhibited excellent biocompatibility, and significantly promoted the proliferation of HUVECs and HaCaT cells.

### Cellular behavior

**Fig. 3:**
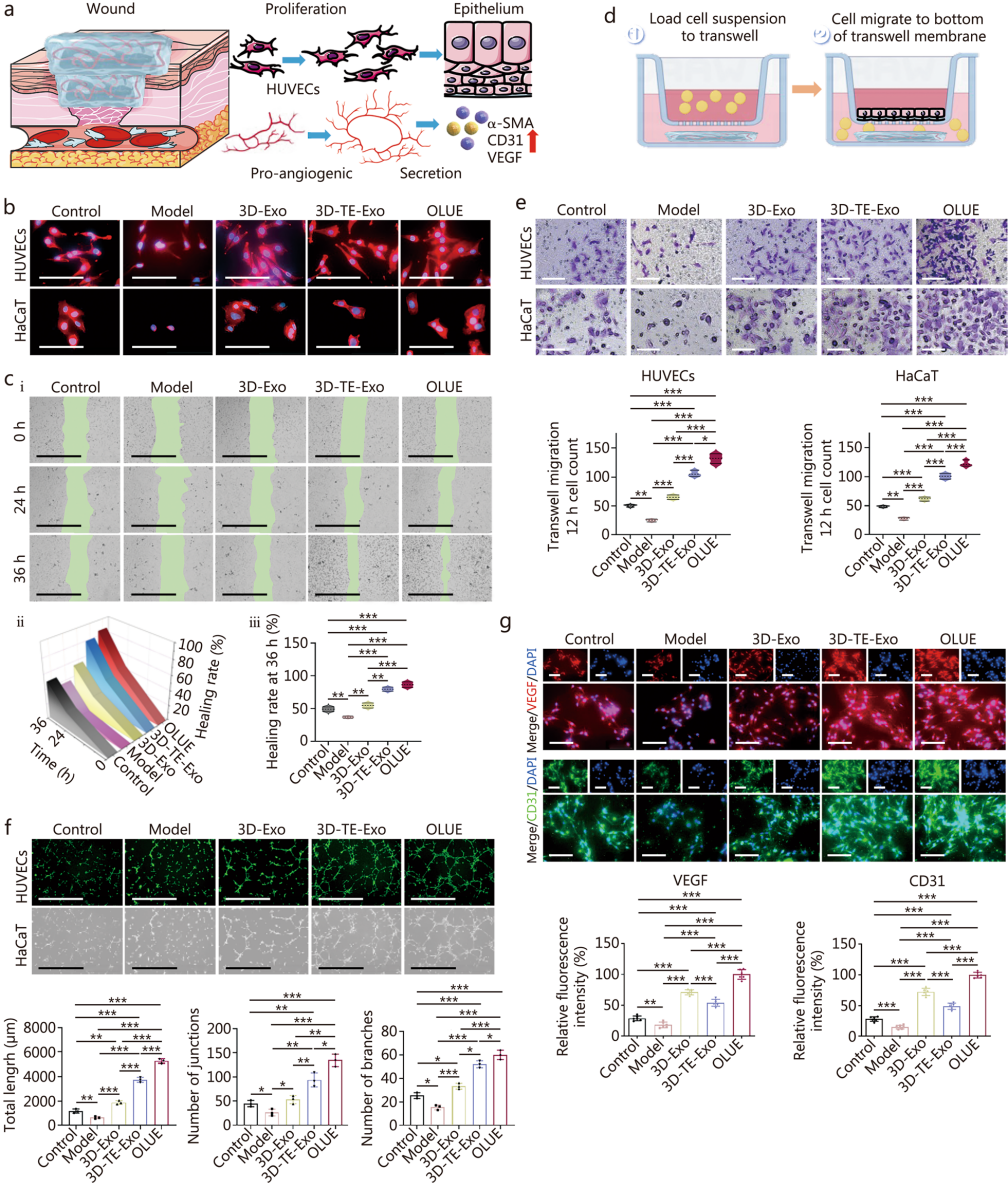
Cell migration and angiogenesis properties of OLUE hydrogel. **a** Schematic diagram of OLUE hydrogel on HUVEC cell proliferation, migration, and angiogenesis. **b** Immunostaining for cytoskeleton F-actin of HUVECs and HaCaT cells (scale bar = 10 μm). **c** Cell migration results of RAW264.7 + HUVECs + HaCaT cells: representative micrographs of different groups at 0, 24, and 36 h (scale bar = 1000 μm) (i), migration trends of different groups at 0, 24 and 36 h (*n* = 5) (ii), and healing rate of different groups at 36 h (*n* = 5) (iii). **d** Schematic diagram of HUVECs and HaCaT cells Transwell migration. **e** Representative micrographs (scale bar = 200 μm) and quantitative analysis (*n* = 3) of OLUE on Transwell migration 12 h of HUVECs and HaCaT cells. **f** Representative images (scale bar = 200 μm) and quantitative analysis (*n* = 5) of tube formation assays of HUVECs treated with H_2_O_2_, 3D-Exo, 3D-TE-Exo, or OLUE on Matrigel. **g** Representative micrographs (scale bar = 200 μm) and quantitative analysis (*n* = 3) of VEGF and CD31 by HUVECs incubated in different preparations for 24 h (*n* = 5). ^*^P < 0.05, ^**^P < 0.01, ^***^P < 0.001. 3D-TE-Exo exosome derived from trace element-supplemented medium, 3D-Exo exosome derived from standard medium, RAW264.7 mouse monocytic macrophage leukemia cells, HUVECs human umbilical vein endothelial cells, HaCaT human immortal keratinocyte line, OLUE ultraviolet light-irradiated oxidized hyaluronic acid and lipoic acid-grafted chitosan constructed hydrogel for exosomes, H_2_O_2_ hydrogen peroxide, α-SMA α-smooth muscle actin, CD31 platelet endothelial cell adhesion molecule-1, VEGF vascular endothelial growth factor, DAPI 4',6-diamidino-2-phenylindole

The ability of dressings to promote cell proliferation, migration, and angiogenesis is crucial (Fig. 3a). The cytoskeleton not only serves as a structural scaffold for cells but also plays a role in organelle transport, cell division, cell movement, and signal transduction. Upon inflammatory stimulation, the distribution of F-actin changes, becoming more concentrated around the nucleus (Fig. 3b). In contrast, treatment alleviated this cellular stress, leading to a more uniform distribution of F-actin rather than nuclear localization. Rearrangement of the intracellular cytoskeleton enhances cell stability, making subsequent migration more efficient [50]. The speed of cell migration significantly affects the efficiency of wound healing [51]. Macrophages, endothelial cells, and keratinocytes are characteristic components of the wound microenvironment [52]. Because of the complexity of the wound microenvironment, studying the migration of one cell type cannot fully demonstrate the healing-promoting effect of hydrogels. To better simulate the actual wound microenvironment and objectively evaluate the ability of the hydrogel to promote cell migration, we established a monolayer co-culture model of macrophages, endothelial cells, and keratinocytes. In the model group, migration efficiency was further reduced (Fig. 3c), with a scratch closure rate of (36.76 ± 1.79)% at 36 h. We hypothesized that macrophages secrete several pro-inflammatory cytokines, resulting in oxidative stress that reduces HUVEC and HaCaT cell activity, thereby delaying cell migration. In contrast, migration ability was restored in the treatment group. A schematic of the vertical migration experiment is shown in Fig. 3d, and the vertical migration results for HUVECs and HaCaT cells were consistent with those of horizontal migration (Fig. 3e). These findings indicated that OLUE strongly promoted cell proliferation, with 3D-TE-Exo playing a crucial role. During wound healing, endothelial cells promote angiogenesis by secreting various vasoactive substances [53]. Rapid vascular reconstruction is essential for the repair and regeneration of tissue [54]. In the model group, the cells were dispersed, with no obvious vascular network formation (Fig. 3f). Inflammation significantly inhibited angiogenesis, which explains the delayed tissue repair in the inflamed areas. The formulation accelerated angiogenesis, with 3D-TE-Exo exhibiting longer vascular structures, more connections, and increased branching than 3D-Exo group. Given the potent pro-angiogenic effects of the hydrogel, we further explored its mechanism of action. VEGF plays a positive role in wound healing by promoting angiogenesis and modulating the inflammatory response [55]. CD31 is currently the best endothelial differentiation marker and is not expressed in non-endothelial tissues or tumors. Inflammation suppressed the secretion of VEGF and CD31 by endothelial cells (Fig. 3g), whereas the formulation significantly enhanced the expression of VEGF and CD31. This indicates that 3D-TE-Exo accelerates angiogenesis by promoting the expression of VEGF and CD31. Overall, 3D-TE-Exo demonstrated superior cell migration and angiogenesis-promoting capabilities compared with 3D-Exo, highlighting their greater potential for clinical applications.

### Anti-inflammatory and antioxidant effects

**Fig. 4:**
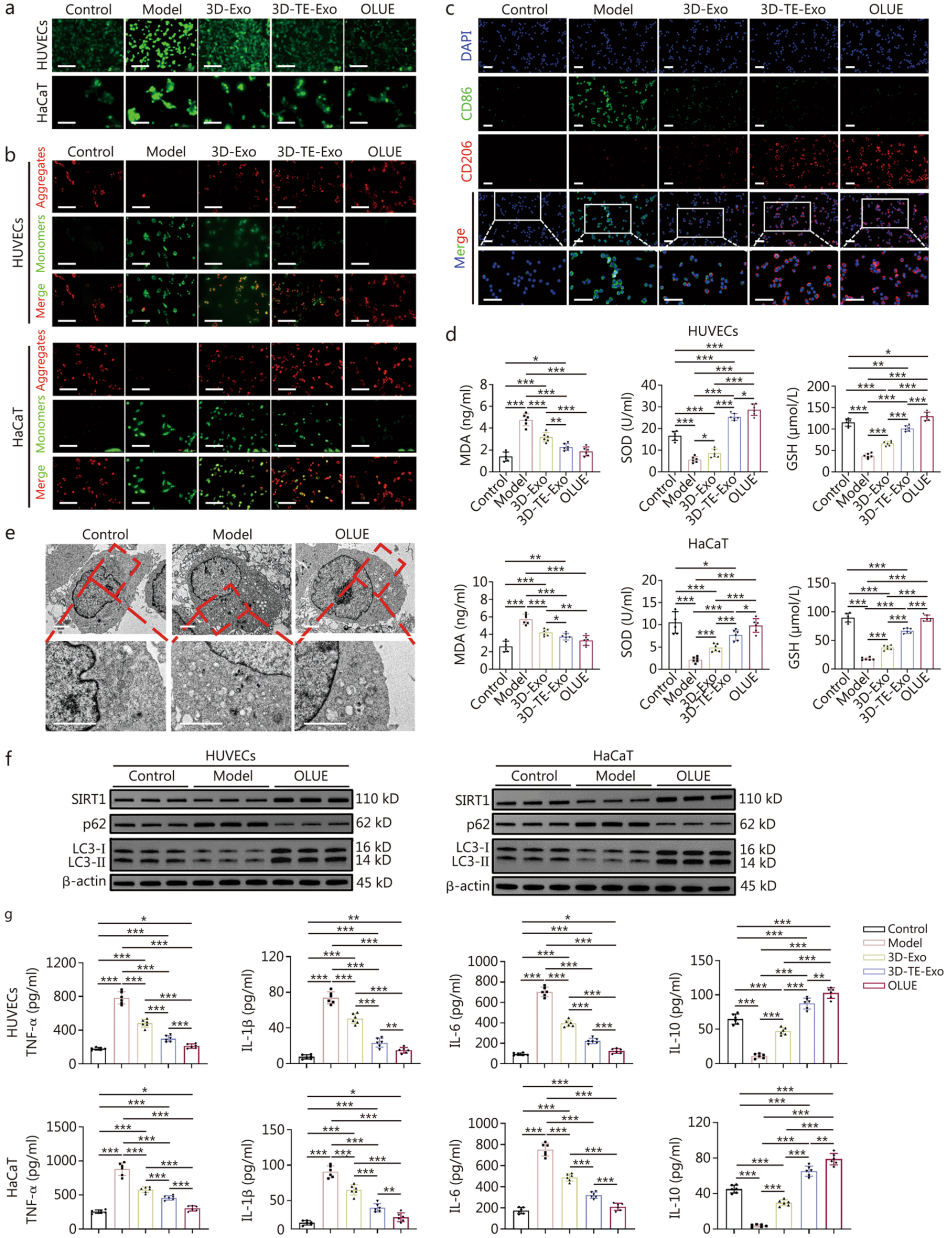
Anti-inflammatory and antioxidant mechanisms of OLUE hydrogel. **a** Representative fluorescence images of H_2_O_2_-induced HUVECs and HaCaT stained with DCFH-DA (scale bar = 200 μm). **b** Representative plot of mitochondrial membrane potential shown by JC-1 staining of HUVECs and HaCaT cells (scale bar = 20 μm). **c** Representative fluorescence images of RAW264.7 macrophage polarization by CD86 (M1, green), CD206 (M2, red), and DAPI (blue) (scale bar = 40 μm). **d** Oxidative stress indexes in HUVECs and HaCaT cells, including MDA, SOD, and GSH (*n* = 6). **e** TEM analysis of autophagosome distribution in HaCaT cells (scale bar = 2 μm). **f** Expression of SIRT1 and autophagy pathway proteins in HUVECs and HaCaT cells. **g** The levels of TNF-α, IL-1β, IL-6, and IL-10 in culturing medium of HUVECs and HaCaT cells after incubation for 1 d (*n* = 6). ^*^P < 0.05, ^**^P < 0.01, ^***^P < 0.001. HUVECs human umbilical vein endothelial cells, HaCaT human immortal keratinocyte line, RAW264.7 mouse monocytic macrophage leukemia cells, DCFH-DA 2',7'-dichlorodihydrofluorescein diacetate, JC-1 5,5′,6,6′-tetrachloro-1,1′,3,3′-tetraethyl-imidacarbocyanine iodide, CD86 cluster of differentiation 86, CD206 mannose receptor, DAPI 4',6-diamidino-2-phenylindole, MDA malondialdehyde, SOD superoxide dismutase, GSH glutathione, TNF-α tumour necrosis factor-alpha, IL-1β interleukin-1β, IL-6 interleukin-6, IL-10 interleukin-10, TEM transmission electron microscopy, SIRT1 silent information regulator 1, p62 sequestosome 1, LC3 microtubule-associated protein light chain 3, H_2_O_2_ hydrogen peroxide, ROS reactive oxygen species, 3D-TE-Exo exosome derived from trace element-supplemented medium, 3D-Exo exosome derived from standard medium, OLUE ultraviolet light-irradiated oxidized hyaluronic acid and lipoic acid-grafted chitosan constructed hydrogel for exosomes

Suppressing inflammation is a crucial strategy for promoting chronic wound healing. As a natural byproduct of O_2_ metabolism, ROS can accumulate excessively in cells under various stimuli, leading to oxidative stress, DNA damage, and enzymatic dysfunction, thereby causing cellular toxicity [56]. In the control group, only minimal ROS expression was observed (Fig. 4a; Additional file 1: Fig. S10). However, inflammation significantly increased intracellular ROS levels. The treatment groups exhibited a marked reduction in ROS expression, with 3D-TE-Exo demonstrating a particularly pronounced effect. OLUE further enhanced the clearance of ROS. The mitochondrial membrane potential (ΔΨm) is critical for maintaining normal mitochondrial function, as its stability is directly related to cellular energy supply and survival [57]. Using the mitochondrial-specific fluorescent dye JC-1, we examined the effect of OLUE on the ΔΨm changes in HUVECs and HaCaT cells induced by H_2_O_2_. When the membrane potential is high, JC-1 forms aggregates (J-aggregates) in the mitochondrial matrix, emitting red fluorescence. Conversely, when the membrane potential decreases, JC-1 dissociates into monomers that emit green fluorescence. After OLUE treatment, the JC-1 polarization ratio increased, indicating restoration of mitochondrial membrane potential (Fig. 4b), and suggesting that OLUE can mitigate H_2_O_2_-induced mitochondrial dysfunction in HUVECs and HaCaT cells. Furthermore, 3D-TE-Exo exhibited efficacy in regulating the mitochondrial membrane potential.

Inflammatory response is a crucial phase in normal skin repair, and its regulation plays a key physiological role in wound healing. In the early stages of inflammation, macrophages play a pivotal role in wound healing by phagocytosing cellular debris and pathogens, and regulating inflammation through polarization-dependent cytokine secretion [58]. Using the neutral red uptake assay, we found that inflammation suppressed macrophage phagocytic activity (Additional file 1: Fig. S11) and affected phenotype transition (Fig. 4c). However, OLUE treatment not only enhanced macrophage phagocytosis but also promoted a shift from the pro-inflammatory M1 phenotype to the anti-inflammatory M2 phenotype, thereby exerting potent anti-inflammatory effects. Additionally, GSH and SOD, key components of the natural antioxidant defense system of the body, help eliminate accumulated superoxide and ROS, thereby reducing tissue damage and enhancing the antioxidant capacity of the skin. This ultimately creates a favorable microenvironment for diabetic wound healing [58]. MDA, a product of lipid peroxidation, is an indicator of oxidative stress [59]. OLUE treatment effectively counteracted the inflammation-induced increase in MDA levels (Fig. 4d) and significantly reduced cellular oxidative stress. The recovery of intracellular SOD and GSH activities further confirmed this antioxidative effect. It is well known that mitochondria are the primary sites of adenosine triphosphate (ATP) production, and ATP is the most important energy metabolite involved in various life processes. Inflammatory conditions suppressed ATP production in the mitochondrial respiratory chain (Additional file 1: Fig. S12). However, 3D-TE-Exo significantly restored ATP levels, while OLUE further enhanced ATP generation capacity. Autophagy plays a crucial role in wound healing by regulating the inflammatory response, preventing excessive inflammation-induced tissue damage, and maintaining normal cellular physiological functions [60]. Under inflammatory stimulation, significant accumulation of autophagic vesicles and a swollen endoplasmic reticulum were observed in HaCaT cells, indicating that inflammation severely impaired autophagic activity (Fig. 4e). To investigate the function of autophagy further, we examined the expression of autophagy proteins. Consistent with the Bio-TEM results, OLUE treatment activated SIRT1 protein expression, downregulated p62 levels, and increased LC3-I/II expression (Fig. 4f; Additional file 1: Fig. S13), effectively restoring autophagic activity that was suppressed by inflammation. TNF-α, IL-1β, and IL-6 are essential inflammatory mediators involved in various pathological conditions. IL-10, a multifunctional cytokine with potent immunoregulatory properties, is critical in controlling inflammation [61]. Under inflammatory stimulation, TNF-α levels in HUVECs and HaCaT cells significantly increased, along with elevated IL-1β and IL-6 levels, whereas IL-10 expression decreased (Fig. 4g). However, OLUE treatment markedly reversed these effects, which could be attributed to the robust inflammation regulating capability of 3D-TE-Exo.

### 3D-TE-Exo exerts immunomodulatory effects via C1QBP

The skin repair effects induced by Exo stem from their various functional molecules. Our preliminary results indicated that, compared to 3D-Exo, 3D-TE-Exo exhibits superior pro-proliferative, pro-migratory, angiogenic, antioxidant, and anti-inflammatory effects. We hypothesized that TE pre-treatment alters the function of MSCs and affects the cargo of 3D-TE-Exo. Many proteins in Exo play crucial roles in regulating gene expression and participate in diverse biological and pathological processes [62]. To investigate the proteins affected by TE and the mechanisms underlying Exo-mediated skin repair, we conducted a proteomic analysis (APExBIO, Houston, USA). The results including the Gene Ontology (GO) enrichment bubble chart and Gene Set Enrichment Analysis (GSEA), indicated that 3D-TE-Exo are closely associated with the complement pathway (Additional file 1: Fig. S14a). Differential protein heatmaps revealed that the level of C1QBP in 3D-TE-Exo was significantly higher than in 3D-Exo. As a classical complement pathway protein, C1QBP inhibits the mitochondrial permeability transition pore, thereby protecting against oxidative stress-induced damage [63, 64]. The experimental results (Additional file 1: Fig. S14b) confirmed that C1QBP was significantly elevated in 3D-TE-Exo compared with in 3D-Exo. Transcriptomic analysis revealed differential gene expression between MSCs and TE-MSCs (Additional file 1: Fig. S15a), Kyoto Encyclopedia of Genes and Genomes enrichment highlighted the complement and coagulation cascades pathway (Additional file 1: Fig. S15b), and GO enrichment (Additional file 1: Fig. S15c) plus GSEA (Additional file 1: Fig. S15d) further characterized enriched biological processes/pathways. To validate this hypothesis, TE-MSCs were pretreated with *C1qbp* siRNA for 24 h, and the resultant 3D-TE-Exo-*siC1qbp* was collected. Western blotting analysis revealed a marked decrease in C1QBP levels in both TE-MSCs- *siC1qbp* and 3D-TE-Exo-*siC1qbp* following knockdown (Additional file 1: Fig. S16a, b). According to the above data (Additional file 1: Fig. S8; Fig. 3; Fig. 4), 3D-TE-Exo alleviates oxidative stress and promotes cell proliferation and migration. However, EdU staining of cells treated with 3D-TE-Exo-*siC1qbp* revealed a reduction in the proliferative effect, which was even lower than that observed with 3D-Exo-*siNC* (Additional file 1: Fig. S16c), indicating that the inhibition of C1QBP reverses the proliferation-promoting effects of 3D-TE-Exo on HUVECs and HaCaT cells. Further investigations showed that although the 3D-TE-Exo-*siC1qbp* retained some ability to restore mitochondrial membrane potential, its effect was lower than that of 3D-TE-Exo-*siNC* (Additional file 1: Fig. S16d). Additionally, the pro-migratory effect of 3D-TE-Exo-*siC1qbp* on HUVECs and HaCaT cells was diminished, with reductions observed in both migration distance and the number of migrating cells compared to the 3D-TE-Exo-*siNC* group (Additional file 1: Fig. S16e, f). Concurrently, the transcriptomic results from MSCs indicated alterations in the extracellular matrix structure of TE-MSCs. Extracellular matrix remodeling and cytoskeletal dynamics are known to influence multivesicular body formation and Exo biogenesis. In conjunction with Fig. 1c, we speculated that TE pretreatment may also affect the ability of MSCs to assemble Exo. Collectively, these results indicate that culturing MSCs with TE not only alters the expression of the complement pathway within MSCs but also enhances Exo assembly efficiency. The anti-inflammatory and wound healing mechanisms of 3D-TE-Exo are primarily attributed to C1QBP. We established a method to obtain high-concentration and high-bioactivity Exo, thereby laying the foundation for innovative strategies in diabetic foot care.

### In vivo therapeutic efficacy and safety of the hydrogel

The in vivo degradation properties of a hydrogel directly affect its pharmacological efficacy and biosafety, and frequent dosing can interfere with wound healing. Therefore, it is crucial to develop a hydrogel with an appropriate degradation profile to reduce dosing frequency. In this study, OHA-LACS hydrogel containing 3D-TE-Exo was subcutaneously injected into the abdominal region of rats and subsequently exposed to brief UV irradiation to form OLUE hydrogel, with the aim of observing its in vivo retention. The results demonstrated that at a body temperature of 37 °C and within the complex in vivo environment, the OLUE hydrogel exhibited an ideal degradation rate and was completely metabolized by day 14 (Additional file 1: Fig. S17a). Furthermore, the biosafety of the hydrogel was evaluated using routine blood tests, serum biochemistry, and histopathological analyses. Compared to normal rats, those treated with the hydrogel showed no significant changes in liver and kidney function indices (Additional file 1: Fig. S17b), and all routine blood parameters remained within normal ranges (Additional file 1: Fig. S17c), indicating that the presence of TE in Exo did not adversely affect these indices. H&E staining of the skin at the hydrogel retention site revealed no significant inflammatory cell infiltration or damage to the skin integrity (Additional file 1: Fig. S17d). Also, no signs of inflammation were observed in the major organs (heart, liver, spleen, lung and kidney) (Additional file 1: Fig. S17e). These results further confirmed the safety of the hydrogel. In summary, the OLUE hydrogel is a promising localized controlled-release drug delivery system for clinical applications.

### Hemostatic and coagulation properties of the hydrogel

The wound healing process for chronic wounds is divided into hemostasis, inflammation, proliferation, and remodeling phases, with hemostasis being the critical initial step [65]. We evaluated the hemostatic performance of the OLUE using rat tail bleeding, liver injury, and cardiac injury models. The results showed that the OLUE exhibited excellent adhesion to the injured tissue surface, remaining firmly attached even under conditions involving stretching and flexing without any noticeable residue (Additional file 1: Fig. S18a-c). In the untreated wounds, bleeding was rapid and continuous. However, upon application of the hydrogel, hemostasis was immediately achieved, and subsequent severe bleeding was prevented. Quantitative analysis revealed that blood loss in the hydrogel-treated group was significantly reduced compared to that in the control group. Moreover, OLUE demonstrated superior in vitro hemostatic performance compared to OHA-LACS, primarily because of its rapid gelation rate. An in vitro whole blood coagulation assay, a common method for evaluating hemostatic agents, showed that the clotting time in the control group exceeded 5 min. In contrast, the hemostatic time of both OHA-LACS and OLUE hydrogels was significantly shortened. In fact, blood in the control group remained fluid after being inverted in a centrifuge tube for 1 min, whereas the hydrogel-treated blood showed clear coagulation (Additional file 1: Fig. S18d). In summary, the excellent hemostatic and coagulation properties of OLUE are mainly attributed to: 1) its superior adhesive ability, which forms an effective physical hemostatic barrier; 2) the presence of positive charges in the hydrogel that attract negatively charged platelets via electrostatic interactions, thereby promoting clot formation; 3) its swelling capacity, which aids in the absorption of red blood cells and platelets; and 4) the catechol structure within the hydrogel, which imparts certain anticoagulant properties. Based on these characteristics, OLUE shows potential for clinical applications in the hemostatic treatment of both acute and chronic wounds.

### Full-thickness wound healing

Given the excellent biological properties of OLUE, we investigated its effects on the acceleration of full-thickness wound healing. The experimental protocol is illustrated in Additional file 1: Fig. S19a. A circular wound with a diameter of 0.8 cm was created on the dorsal skin of healthy rats using a biopsy punch, and different formulations were applied to the wound area. The results (Additional file 1: Fig. S19b) demonstrated that OLUE significantly accelerated the healing of full-thickness wounds, with the wounds nearly completely closed by day 7. Additional file 1: Fig. S19c presents a schematic diagram of the wound healing process, indicating that the OLUE group exhibited the fastest healing rate. Notably, the healing efficiency of 3D-TE-Exo was superior to that of 3D-Exo. On day 7, the wound healing rate in the model group was (50.42 ± 6.17)%, with a wound area of (0.25 ± 0.03) cm^2^, whereas the OLUE group achieved a healing rate of (91.95 ± 4.11)% and a wound area of only (0.04 ± 0.02) cm^2^. Moreover, the recovery of body weight during wound healing is a crucial indicator of the overall health status of the animals. The OLUE group exhibited the most substantial weight recovery (Additional file 1: Fig. S19d), with the average body weight reaching (209.55 ± 5.94) g on day 7 compared to only (185.22 ± 5.87) g in the model group. To further assess the wound healing and collagen deposition-promoting effects of OLUE, histological evaluation was performed using H&E and Masson staining. H&E staining (Additional file 1: Fig. S19e) revealed that by day 7, the model group had only minimal epidermis, whereas the OLUE group exhibited a nearly complete epidermal structure, accompanied by abundant fibroblasts, neovascularization, hair follicles, and well-organized connective tissue. The reepithelialization rate in the OLUE group was the highest at (90.83 ± 3.15)%. Masson’s staining, which was used to observe newly formed collagen, showed that by day 7, the model group expressed only a small amount of collagen, highlighting the challenges of achieving full-thickness wound healing (Additional file 1: Fig. S19f). In contrast, the OLUE group exhibited a pronounced red epidermal layer and substantial collagen deposition with the most complete fibrous structure, with a collagen deposition rate of (76.62 ± 4.27)%. Throughout the experiment, we observed that the healing effects of both 3D-TE-Exo and OLUE were superior to those of 3D-Exo and clinically used Tegaderm™. The accelerated wound closure achieved by OLUE was attributed to the combined effects of the bioactive 3D-TE-Exo and self-adaptive hydrogel. 3D-TE-Exo, enriched with C1QBP, orchestrates the complement-mitochondria-autophagy circuitry, alleviates oxidative stress, restores mitochondrial function, and modulates immune responses. The OLUE hydrogel enabled a responsive and sustained Exo release, preserved Exo bioactivity, and provided mechanical support. This synergistic approach collectively facilitated enhanced keratinocyte proliferation, endothelial cell-driven angiogenesis, and extracellular matrix remodeling, ultimately expediting full-thickness wound repair.

### Healing of DFU

#### Effectiveness evaluation

**Fig. 5:**
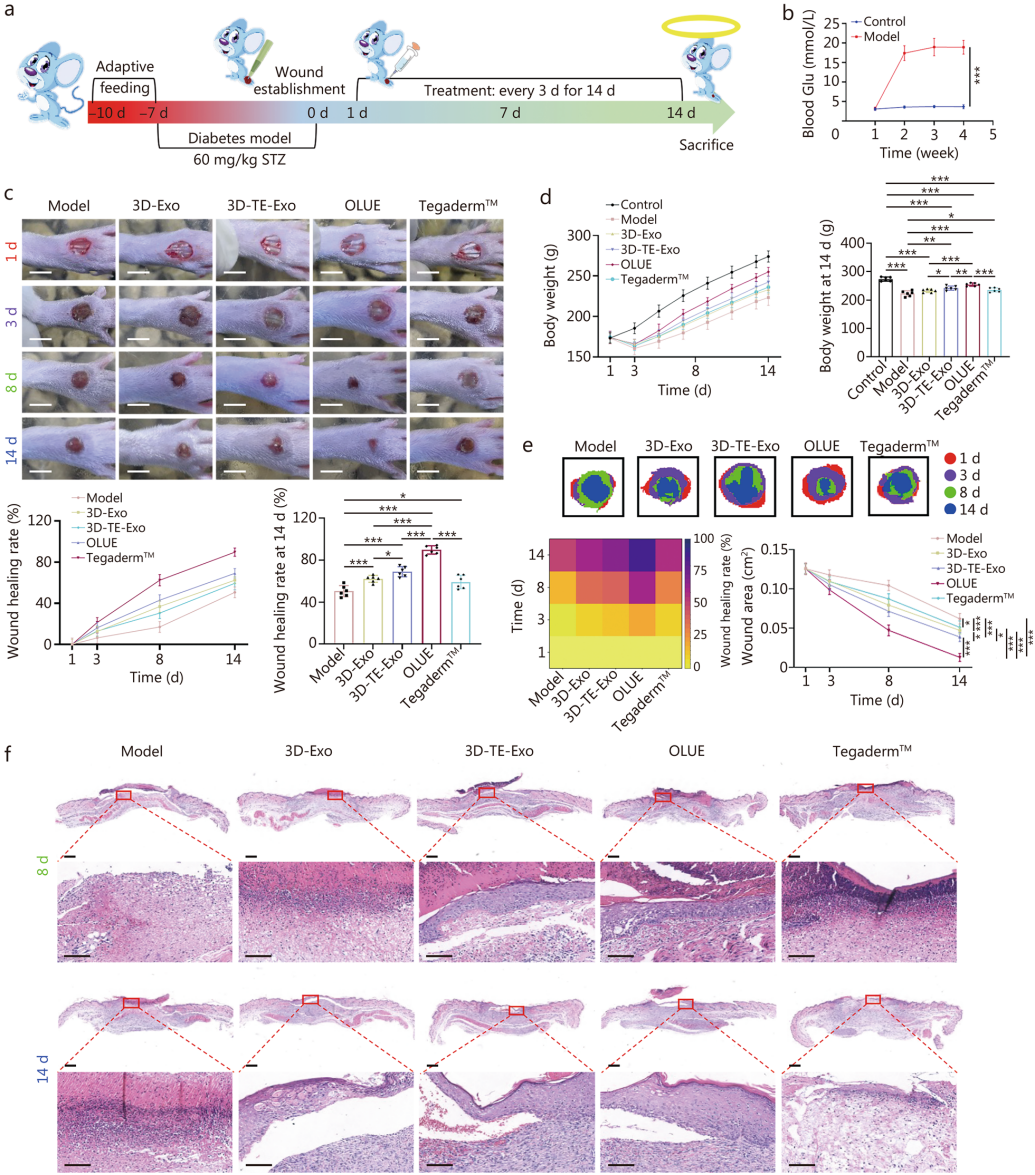
In vivo evaluation of OLUE hydrogel in DFU rats. **a** Schematic diagram of the establishment and treatment of DFU model. **b** Changes of blood Glu in rats (*n* = 4).** c** Macroscopic images taken at 1, 3, 8, and 14 d of each group (scale bar = 0.4 cm) and wound healing rate (*n* = 6). **d** Body weight changes in rats at 14 d (*n* = 6). **e** Heat map and quantitative analysis of wound healing trends during treatment (*n* = 6). **f** Representative images of H&E staining of wounds for different groups at 8 and 14 d (scale bar = 100 μm). ^*^P < 0.05, ^**^P < 0.01, ^***^P < 0.001. DFU diabetic foot ulceration, H&E staining hematoxylin-eosin staining, 3D-TE-Exo exosome derived from trace element-supplemented medium, 3D-Exo exosome derived from standard medium, OLUE ultraviolet light-irradiated oxidized hyaluronic acid and lipoic acid-grafted chitosan constructed hydrogel for exosomes, STZ streptozocin, Glu glucose

Given that OLUE significantly accelerated full-thickness wound healing in healthy rats, we evaluated its efficacy in DFU repair. The experimental protocol is illustrated in Fig. 5a. A type 1 diabetic rat model was established by a single intraperitoneal injection of STZ [66]. Diabetic rats exhibited the classic symptoms of diabetes, including polydipsia, polyphagia, and polyuria, accompanied by a marked decline in energy and responsiveness. Rats with sustained blood Glu levels above 16.7 mmol/L were selected for subsequent hydrogel testing (Fig. 5b). A circular wound with a diameter of 0.4 cm was created on the dorsal surface of the foot using a biopsy punch, and different treatment regimens were applied. Photographic records of the foot during treatment are shown in Fig. 5c, with the wound areas gradually decreasing over time in all groups. Due to the inflammatory and hyperglycemic microenvironment, the wounds in the model group became ulcerated and healed very slowly. In contrast, the OLUE group exhibited a significantly accelerated healing rate. Furthermore, recovery of body weight during wound healing is critical. Normal rats gradually gained weight, while diabetic rats initially gained weight due to increased food consumption, but subsequently exhibited slowed weight gain and even weight loss later, especially after the onset of DFU (Fig. 5d). After OLUE treatment, the rate of weight gain was significantly restored, with the model group weighing (223.45 ± 9.17) g on day 14 compared to (255.23 ± 5.58) g in the OLUE group. This is illustrated in a schematic of wound healing (Fig. 5e). On day 14, the wound healing rate in the model group was (50.64 ± 5.28)% with a wound area of (0.06 ± 0.01) cm^2^, whereas the OLUE group achieved a healing rate of (89.71 ± 4.01)% with a wound area of only (0.01 ± 0.01) cm^2^. Consistent with our expectations, the healing efficiency of 3D-TE-Exo was superior to that of 3D-Exo. Finally, pathological staining was performed to assess skin wound healing in each group. H&E staining results (Fig. 5f) from days 8 and 14 revealed that the model group still exhibited substantial inflammatory cell infiltration, indicating that, without intervention, the inflammatory state in the DFU persisted and healing was delayed. Notably, the OLUE group not only showed a well-reconstructed epidermis but also demonstrated the presence of hair follicles and neovascularization, suggesting that the OLUE hydrogel significantly accelerated the DFU healing. Collagen deposition, remodeling, and follicle regeneration are key indicators of skin integrity repair, as they contribute to the tensile strength and overall integrity of the epidermis.

#### Histopathology

**Fig. 6:**
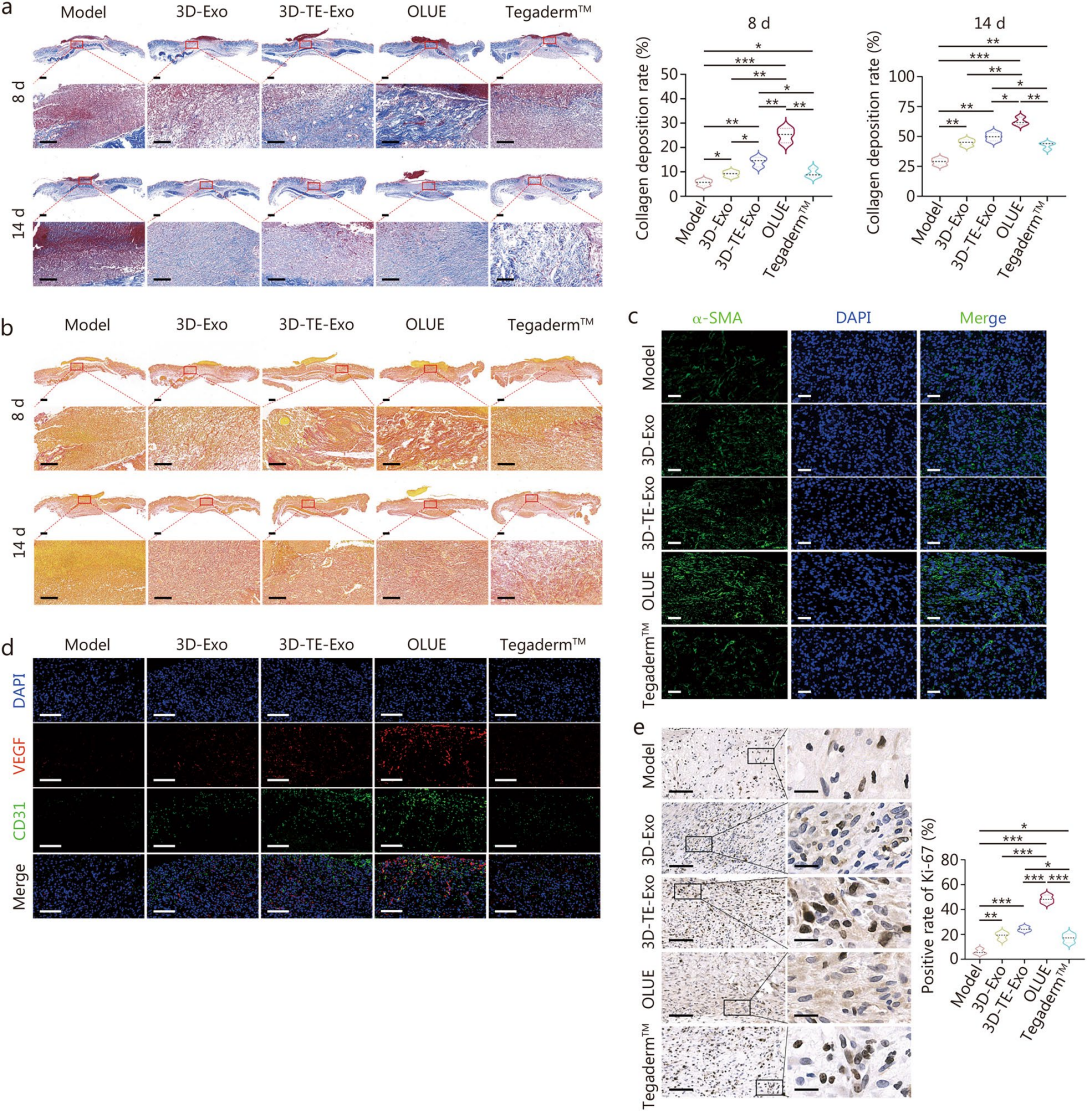
Histopathology of wound sites in the proliferation phase. **a** Representative images (scale bar = 100 μm) and quantitative analysis (*n* = 3) of Masson staining of different treatments at 8 and 14 d. **b** Representative images of picro-Sirius red staining of different treatments at 8 and 14 d (scale bar = 100 μm). **c** Representative images of α-SMA of different treatments at 14 d (scale bar = 40 μm). Green, α-SMA; Blue, DAPI. **d** Representative images of VEGF and CD31 staining of different treatments at 14 d (scale bar = 1000 μm). Red, VEGF; Green, CD31; Blue, DAPI. **e** Representative images of Ki-67 staining of different treatments (scale bar = 80 μm), and the quantification of Ki-67 deposition (%) at 14 d (*n* = 3). ^*^P < 0.05, ^**^P < 0.01, ^***^P < 0.001. α-SMA α-smooth muscle actin, DAPI 4',6-diamidino-2-phenylindole, VEGF vascular endothelial growth factor, CD31 platelet endothelial cell adhesion molecule-1, Ki-67 kiel-67, 3D-TE-Exo exosome derived from trace element-supplemented medium, 3D-Exo exosome derived from standard medium, OLUE ultraviolet light-irradiated oxidized hyaluronic acid and lipoic acid-grafted chitosan constructed hydrogel for exosomes

Masson’s (Fig. 6a) and Sirius red staining (Fig. 6b) were used to evaluate collagen maturation in the regenerated tissue. Extensive red necrotic tissue and sparse blue collagen fibers were observed in the model group, whereas the OLUE group exhibited abundant blue collagen deposition. In Sirius red staining, immature collagen, generally considered type III collagen, appears yellow, whereas mature collagen and organized fibers appear intensely orange, which is typically indicative of type I collagen. On day 8, the model group displayed sparse light yellow staining, indicating that most of the collagen was immature. In contrast, the OLUE group showed relatively dense light yellow staining. By day 14, the OLUE group exhibited mature collagen fibers with strong orange staining distributed widely, suggesting favorable healing. In contrast, although the control and Tegaderm™ groups showed somewhat denser staining on day 8, they remained predominantly yellow, indicating persistence of immature collagen. α-SMA, a marker of fibroblast-to-myofibroblast differentiation, plays a critical role in wound healing. During the proliferation phase of wound repair, the sustained expression of α-SMA accelerates reepithelialization. The model group exhibited the lowest expression of α-SMA (Fig. 6c; Additional file 1: Fig. S20), consistent with its lower healing rate, whereas OLUE dramatically enhanced α-SMA secretion. The high expression of α-SMA in the OLUE groups indicates increased formation of myofibroblasts during wound healing. Impaired angiogenesis is a major challenge in DFU treatment. To observe neovascularization at the wound site, immunofluorescence staining for VEGF and CD31was performed at various time points. VEGF and CD31 form a complex regulatory network during chronic wound healing. In the model group, almost no expression of VEGF or CD31 was observed (Fig. 6d), confirming that angiogenesis was severely impaired in DFU. In contrast, the OLUE treatment promoted the expression of VEGF and CD31, thereby accelerating neovascularization. 3D-TE-Exo are internalized by endothelial cells, where they release their cargo and modulate the secretion of VEGF and CD31. Ki-67, a well-known marker of cell proliferation, provides important biological insights into the efficacy of DFU treatment. Immunohistochemical analysis showed that the model group exhibited few Ki-67 positive cells (Fig. 6e), indicating suppressed cell proliferation at the DFU site, which is a key factor that contributes to poor wound healing. After OLUE treatment, the number of Ki-67 positive cells increased significantly, demonstrating the efficacy of this therapeutic approach. These results collectively indicate that OLUE enhances collagen and α-SMA secretion and increases cell proliferation rates, thereby promoting reepithelialization of the wound. Simultaneously, OLUE upregulated the expression of VEGF and CD31, thereby accelerating angiogenesis. Overall, OLUE has tremendous potential as a DFU treatment.

#### Microenvironmental regulation

**Fig. 7:**
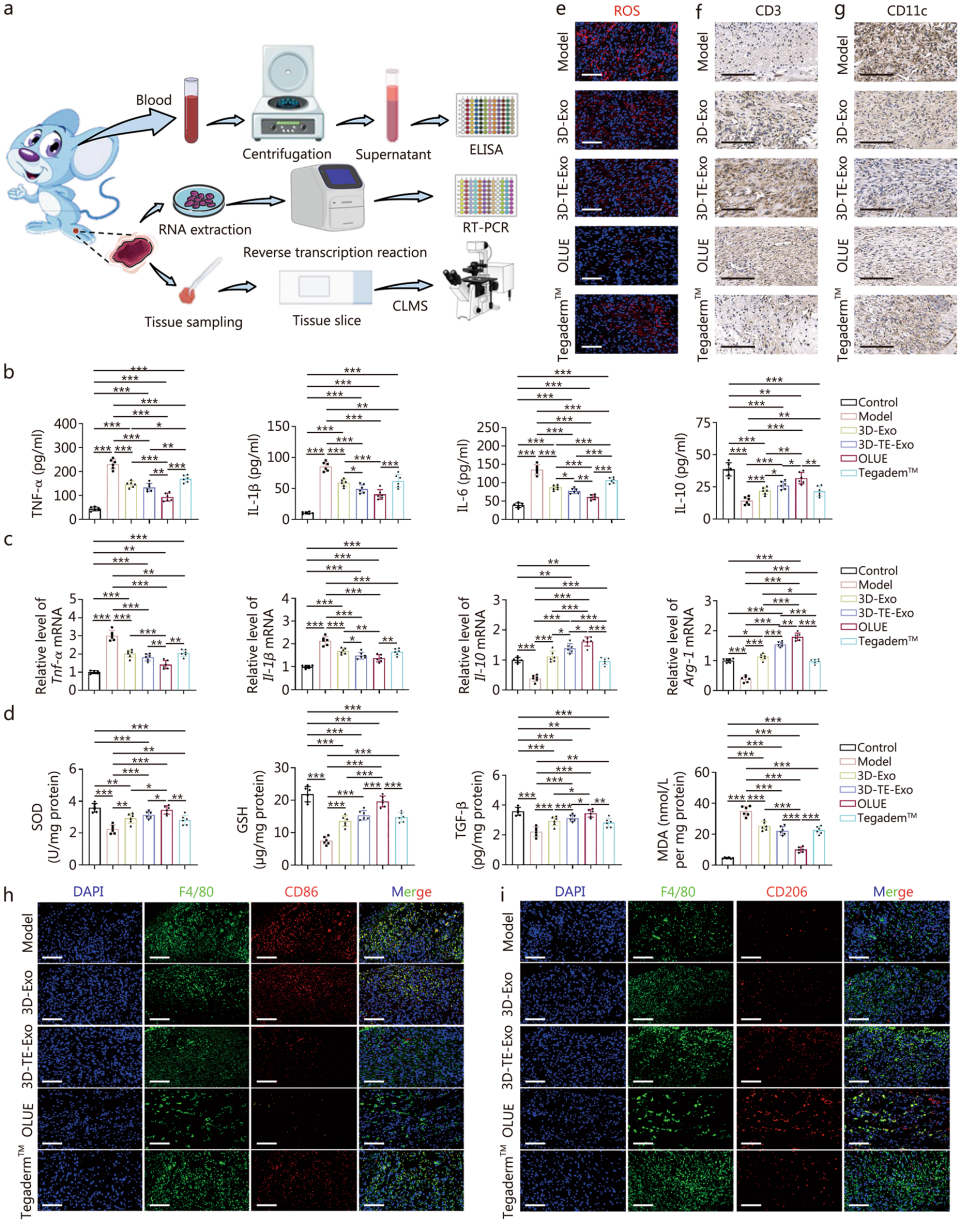
Microenvironment of DFU in rats. **a** Schematic diagram of rat skin and blood test. **b** Cytokine levels in rats at 14 d of treatment, including TNF-α, IL-1β, IL-6, and IL-10 (*n* = 6). **c** Gene levels of *Tnf*-β, *Il-1*β, *Il-10*, and *Arg-1* at 14 d of treatment (*n* = 6). **d** SOD, GSH, TGF-β, and MDA levels in wound site of rats treated for 14 d (*n* = 6). **e** ROS levels at wound sites in rats at 14 d of treatment (scale bar = 100 μm). **f** Immunohistochemistry of CD3 in the skin wounds of diabetic rats treated at 14 d (scale bar = 100 μm). **g** Immunohistochemistry of CD11c in the skin wounds of diabetic rats treated at 14 d (scale bar = 100 μm). **h** M1 macrophages in the skin wounds of diabetic rats (scale bar = 100 μm). Blue, DAPI; Green, F4/80; Red, CD86. **i** M2 macrophages in the skin wounds of diabetic rats (scale bar = 100 μm). Blue, DAPI; Green, F4/80; Red, CD206. ^*^P < 0.05, ^**^P < 0.01, ^***^P < 0.001. ELISA enzyme-linked immunosorbent assay, RT-PCR reverse transcription-polymerase chain reaction, CLSM confocal laser scanning microscope, TNF-α tumor necrosis factor-α, IL-1β interleukin-1β, IL-6 interleukin-6, IL-10 interleukin-10, Arg-1 arginase-1, SOD superoxide dismutase, GSH glutathione, MDA malondialdehyde, TGF-β transforming growth factor-β, ROS reactive oxygen species, CD3 cluster of differentiation 3, CD11c integrin subunit alpha X, F4/80 mouse EGF-like module-containing mucin-like hormone receptor-like 1, CD86 cytotoxic T-lymphocyte-associated protein 4, CD206 mannose receptor, DAPI 4',6-diamidino-2-phenylindole, 3D-TE-Exo exosome derived from trace element-supplemented medium, 3D-Exo exosome derived from standard medium, OLUE ultraviolet light-irradiated oxidized hyaluronic acid and lipoic acid-grafted chitosan constructed hydrogel for exosomes

The DFU microenvironment is highly complex and characterized by hyperglycemia, persistent inflammation, hypoxia, ischemia, dynamic pH with high protease activity, and dysregulation of various immune cells [67]. Pathologically, prolonged and widespread inflammation is one of the major factors that disrupts the normal cascade of DFU healing. Figure 7a illustrates a schematic diagram of how the OLUE modulates the inflammatory microenvironment in rats. In diabetic rats, the levels of proinflammatory cytokines (TNF-α, IL-1β, and IL-6) were significantly elevated (Fig. 7b; Additional file 1: Fig. S21). However, treatment with OLUE and 3D-TE-Exo markedly inhibited the secretion of these cytokines and increased IL-10 concentrations, thereby exerting a robust anti-inflammatory effect (Fig. 7b; Additional file 1: Fig. S21). Moreover, OLUE modulated the expression of DFU-related genes by reducing the mRNA levels of *Tnf-α* and *Il-1β* and increasing the mRNA levels of *Il-10* and *Arg-1* (Fig. 7c), which further alleviated the inflammatory microenvironment in DFU. The dysregulation of the antioxidant defense system adversely affects DFU repair. TGF-β, a multifunctional protein, regulates cell survival, metabolism, growth, proliferation, differentiation, adhesion, migration, and apoptosis [68]. In the model group, levels of SOD, GSH, and TGF-β were significantly decreased, while MDA levels were elevated (Fig. 7d), indicating a disruption of the antioxidant defense system. In contrast, the OLUE group showed significant increases in SOD, GSH, and TGF-β levels, along with a reduction in MDA, demonstrating that OLUE restored the antioxidant defense system in DFU. Excessive accumulation of ROS is detrimental to wound healing, and DFU lesions exhibited high levels of ROS (Fig. 7e), which severely inhibited cell proliferation and angiogenesis. OLUE effectively cleared excess ROS and alleviated oxidative stress. The combination of SIRT1 activation, p62 reduction, and LC3-I/II accumulation in OLUE group provides robust evidence for mitophagy induction at the tissue level (Additional file 1: Fig. S22). Immune cell dysregulation prolongs the inflammatory phase. First, T cells and dendritic cells (DC) were analyzed by immunohistochemistry. The results showed that OLUE-treated skin exhibited the highest CD3 positive area (Fig. 7f), indicating an increased proportion of CD3 positive T cells due to treatment. Conversely, the model group exhibited high CD11c expression, indicating severe infiltration of DC in the skin (Fig. 7g), which was mitigated by OLUE treatment. Macrophage phenotype modulation is a fundamental strategy for reducing autoimmunity and chronic inflammation, thereby accelerating wound repair and tissue remodeling [41]. To investigate the effect of OLUE on the macrophage phenotype, we performed multiplex immunofluorescence staining. The model group showed the highest percentage of CD86 positive cells (Fig. 7h) and the lowest percentage of CD206 positive cells (Fig. 7i), indicating an abundance of pro-inflammatory M1 macrophages at the DFU sites. In contrast, OLUE-treated DFU exhibited a reversal of this profile, with a significant increase in CD206 positive cells in injured tissues, indicating that OLUE can induce the transition of macrophages from the pro-inflammatory M1 phenotype to the anti-inflammatory M2 phenotype.

## Discussion

DFU is a common and disabling complication in diabetic patients, characterized by persistent inflammation, oxidative stress, and impaired tissue regeneration that ultimately leads to chronic, non-healing wounds. These pathological features severely diminish patients’ quality of life and impose a substantial burden on healthcare systems. While MSCs and their secreted Exo have recently garnered considerable attention for their capacity to mediate intercellular communication and promote tissue repair, we propose an innovative strategy that integrates TE (Fe, Mg, Zn, Mn, and Se) into a 3D culture system to modulate the biological function of mesenchymal stem cells (TE-MSCs), thereby generating functionally enhanced engineered Exo (3D-TE-Exo). Our experimental findings demonstrate that, compared to Exo derived from conventional 3D culture (3D-Exo), 3D-TE-Exo exhibits significantly higher particle numbers and concentrations. The 3D culture environment better recapitulates the native in vivo microenvironment, fostering extensive cell-cell and cell-extracellular matrix interactions. This biomimetic setup enables MSCs to adopt more physiologically relevant morphologies, behaviors, and functional states, which in turn enhances Exo yield, bioactivity, and molecular diversity.

Moreover, ICP-MS analysis revealed a substantial enrichment of TE levels within 3D-TE-Exo, underscoring the critical role of TE in regulating cellular function and Exo biogenesis. The balanced incorporation of multiple TE is essential for maintaining physiological homeostasis, given their pivotal roles in cellular signaling, muscle contraction, neurotransmission, and cardiovascular health. However, this aspect is often underappreciated in clinical practice. Notably, the selected TE align closely with the pathological hallmarks of DFU. All 5 elements are deeply involved in redox regulation. Zn, for instance, serves as a cofactor for Cu/Zn SOD, which catalyzes the dismutation of superoxide radicals into O_2_ and H_2_O_2_ [69]. It also inhibits nicotinamide adenine dinucleotide phosphate oxidase, thereby reducing ROS production, supporting immune function, and promoting wound healing. Although oxidative stress is not solely attributable to Fe deficiency, the 2 frequently coexist. Inflammatory or infectious conditions often impair Fe absorption as a host defense mechanism to restrict Fe availability to pathogenic bacteria [70]. Mg plays an indispensable role in cellular activities, including proliferation, differentiation, and activation, while concurrently mitigating inflammation and oxidative stress [71]. Mn is critical for antioxidant defense, carbohydrate and lipid metabolism, and free radical neutralization. Specifically, mitochondrial Mn-SOD serves as a primary enzymatic defense against oxidative damage [72]. Se, an essential micronutrient, exhibits potent anti-inflammatory and antioxidant properties and plays a key role in immune modulation [73]. The synergistic effect of these TE profoundly influences MSC physiology and Exo assembly, culminating in the production of 3D-TE-Exo with superior therapeutic potential. This approach not only offers a promising avenue for DFU treatment but also serves as a reference framework for TE-mediated modulation of MSCs function. Unlike traditional studies that focus on the role of individual elements, our strategy adopts a systems biology perspective to elucidate how a composite TE microenvironment reshapes the immune microenvironment in DFU. This methodology confers several distinct advantages: 1) it more accurately mirrors clinical practices involving combined micronutrient supplementation; 2) it leverages integrated multi-omics analyses to uncover synergistic regulatory networks that would likely be overlooked in single-variable experimental designs.

**Fig. 8:**
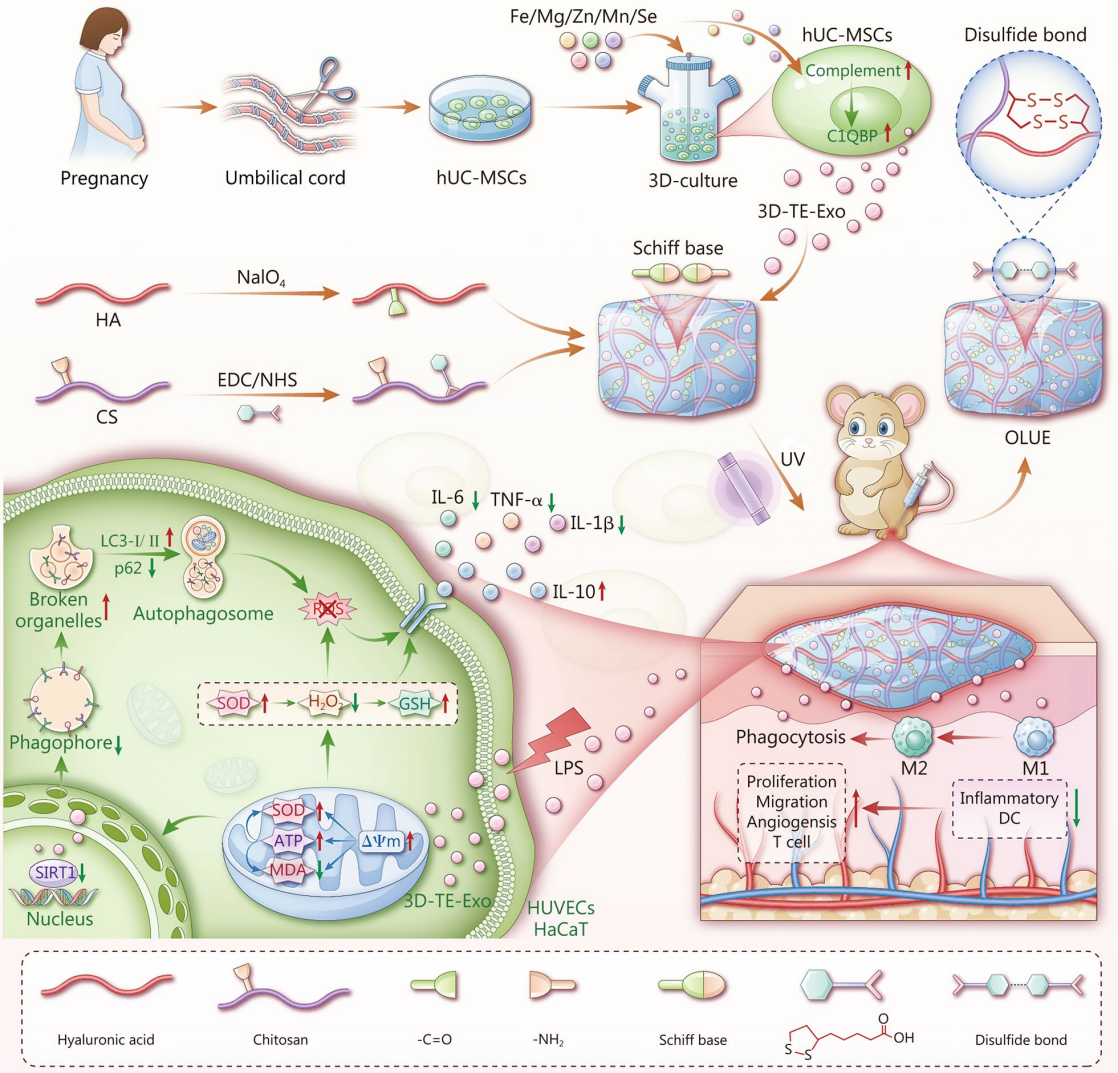
The 3D-TE-Exo with high activity were constructed by Fe-Mg-Zn-Mn-Se multi-TE synergy and 3D dynamic culture. 3D-TE-Exo mitigates inflammation and promotes angiogenesis through a triple mechanism involving C1QBP-mediated regulation of the complement pathway, restoration of mitochondrial membrane potential, and remodeling of autophagic flux, thereby disrupting the pathological cycle. HA hyaluronic acid, CS chitosan, EDC ethyldimethylaminopropyl carbodiimide, NHS N-hydroxysuccinimide, 3D three-dimensional, 3D-TE-Exo exosome derived from trace element-supplemented medium, MDA malondialdehyde, SOD superoxide dismutase, GSH glutathione, TNF-α tumour necrosis factor-alpha, IL-1β interleukin-1β, IL-6 interleukin-6, IL-10 interleukin-10, SIRT1 silent information regulator 1, p62 sequestosome 1, LC3 microtubule-associated protein light chain 3, ATP adenosine triphosphate, ROS reactive oxygen species, HUVECs human umbilical vein endothelial cells, HaCaT human immortal keratinocyte line, H_2_O_2_ hydrogen peroxide, ΔΨm mitochondrial membrane potential, UV ultraviolet, LPS lipopolysaccharide, C1QBP complement 1q binding protein, hUC-MSCs human umbilical cord mesenchymal stem cells, DC dendritic cells

In addition to their high concentration and particle count, 3D-TE-Exo exhibit remarkable biological activity. Integrated proteomic and transcriptomic analyses revealed a significant enrichment of the multifunctional protein C1QBP in 3D-TE-Exo. C1QBP plays a pivotal role in cellular immune function by preserving mitochondrial plasticity and metabolic homeostasis [74, 75]. Preliminary functional validation demonstrated that silencing *C1qbp* markedly attenuated the therapeutic efficacy of 3D-TE-Exo in modulating inflammatory responses, as well as in promoting cell proliferation and migration. These results provide compelling evidence that C1QBP is a key molecular mediator underlying the enhanced bioactivity of TE-engineered Exo. C1QBP is a well-characterized protein essential for mitochondrial oxidative phosphorylation, primarily through its role in facilitating the translation of mitochondrial-encoded proteins. While the precise molecular mechanisms governing its action in the context of Exo-mediated therapy require further elucidation, these initial findings establish a robust foundation for future mechanistic studies. Collectively, this study demonstrates that C1QBP-enriched 3D-TE-Exo modulated the complement pathway in HUVECs and HaCaT cells, restored mitochondrial membrane potential, enhanced ATP production, and activated autophagic flux. As a result, treatment with 3D-TE-Exo leads to a significant reduction in pro-inflammatory cytokines and MDA levels, alongside marked increases in anti-inflammatory cytokine IL-10, GSH, and SOD activity, thereby effectively clearing excessive ROS. Ultimately, 3D-TE-Exo alleviates oxidative stress, restores the proliferative capacity of HUVECs and HaCaT cells, and demonstrates robust tissue repair capabilities in vivo (Fig. 8).

As a promising biomaterial for skin repair, CS and HA-based hydrogels offer numerous advantages, including facile application, rapid and controllable gelation, strong tissue adhesion, excellent biocompatibility, tunable biodegradation, and the capacity to encapsulate and deliver bioactive agents. Given the inherent challenges associated with the in vivo delivery, retention, and stability of Exo, we developed a stimuli-responsive hydrogel delivery platform for precise, localized Exo release. This hydrogel system is based on a double-network structure composed of OHA and LACS. A Schiff base reaction rapidly forms an injectable precursor hydrogel, followed by UV-assisted secondary crosslinking, which significantly enhances the mechanical strength and structural stability of the hydrogel. Importantly, the hydrogel exhibits microenvironment-responsive degradation, specifically tailored to the inflammatory milieu characteristic of DFU, thereby controlling and sustaining Exo release at the wound site. Furthermore, OLUE exhibits exceptional flexibility, strong adhesion, and effective hemostatic properties, offering comprehensive wound protection to support the tissue regeneration process. Moreover, OLUE reduces the levels of TNF-α, IL-1β, IL-6, and MDA, effectively clearing excess ROS, while simultaneously increasing SOD, GSH, and TGF-β. This restoration of the antioxidant defense system helps to alleviate the inflammatory microenvironment in DFU. In addition, our study also showed that OLUE modulates immune cell infiltration by regulating T cell and DC populations and facilitates the polarization of macrophages toward an anti-inflammatory (M2) phenotype. These immunomodulatory effects, combined with enhanced cellular proliferation, collagen deposition, accelerated cell migration, and promoted angiogenesis, collectively contribute to the effective resolution of DFU and significantly accelerate wound healing (Additional file 1: Fig. S23).

This study acknowledges inherent limitations in fully elucidating the synergistic mechanisms underlying the interplay of TE. While the Fe-Mg-Zn-Mn-Se pentameric system demonstrates clear therapeutic potential in targeting the pathological features of DFU, the intrinsic complexity of multi-element synergy currently limits the precise quantification of the individual contribution of each element. This experimental framework is grounded in two core scientific premises. First, the human body functions as a highly integrated biochemical network, in which pathological processes are governed by interconnected regulatory pathways [32]. As such, single-element interventions fail to accurately replicate physiological environments, particularly given the well-documented competitive absorption, functional antagonism, and homeostatic balance among TE [76]. Second, the cascade amplification effects arising from TE interactions have been empirically validated through their influence on the molecular sorting mechanisms involved in Exo cargo loading. It is noteworthy that essential TE remain indispensable in clinical practice due to their fundamental roles as enzymatic cofactors and regulators of redox homeostasis. Regarding the antimicrobial properties of OLUE hydrogel, this study intentionally did not conduct redundant validations, as the antimicrobial mechanism of CS is already extensively documented in the literature. Specifically, CS exerts bacteriolytic effects through electrostatic interactions mediated by its protonated amine groups, which disrupt bacterial cell membranes, a mechanism that has been robustly demonstrated across numerous studies [77, 78]. Therefore, the core innovation of this work centers on the relatively underexplored domain of immune microenvironment modulation, rather than reiterating established antibacterial effects.

## Conclusions

This study addresses the "inflammation-ischemia" cycle in DFU via a multimodal strategy integrating TE-modulated MSCs function, engineered 3D-TE-Exo, stimuli-responsive hydrogel delivery, and molecular mechanism elucidation. 3D-TE-Exo exhibited potent pro-angiogenic and anti-inflammatory, alleviating oxidative stress by modulating the complement pathway, restoring mitochondrial function, boosting ATP production, and activating autophagy, C1QBP as a key mediator. This platform engineers Exo to counter inflammation and delivers them via a wound-adaptive hydrogel, providing an innovative DFU treatment and a foundation for synergistic engineered Exo-smart biomaterial designs in regenerative medicine. Future work will validate C1QBP in large animals, clarify 3D-TE-Exo mechanisms, and advance clinical translation.

## Supplementary Information

**Additional file 1. Methods. Fig. S1** Identification and evaluation of MSCs. **Fig. S2** Zeta potentials of 3D-Exo and 3D-TE-Exo detected by DLS. **Fig. S3** Synthesis and identification of OHA. **Fig. S4** Synthesis and identification of LACS. **Fig. S5** Simultaneous thermal analysis of HA, OHA, CS, LA, LACS, OHA-LACS, and OHA-LACS-UV. **Fig. S6** The XPS spectra of OHA-LACS hydrogel, including C, O, N, and S. **Fig. S7** The swelling rate of OHA-LACS and OHA-LACS-UV hydrogels (*n* = 3). **Fig. S8** Biocompatibility of OLUE hydrogel. **Fig. S9** Hemolytic properties of OHA-LACS-UV hydrogels. **Fig. S10** Quantification of ROS fluorescence intensity by ImageJ software HUVECs and HaCaT cells (*n* = 3). **Fig. S11** Neutral red cell proliferation test (*n* = 6). **Fig. S12** ATP in the mitochondria of HUVECs and HaCaT cells (*n* = 6). **Fig. S13** Quantification of Western blotting results. **Fig. S14** Expression and identification of C1QBP in 3D-TE-Exo. **Fig. S15** Expression and identification of C1QBP in MSCs and TE-MSCs. **Fig. S16** Silence and functional evaluation of C1QBP. **Fig. S17** In vivo retention and safety properties of OLUE hydrogel. **Fig. S18** In vivo hemostasis and coagulation properties of OLUE hydrogel. **Fig. S19** In vivo evaluation of OLUE hydrogel in the healthy rats full-thickness wound. **Fig. S20** Quantification of α-SMA fluorescence intensity by ImageJ software (*n* = 3). **Fig. S21** Immunohistochemistry staining for IL-6 and IL-10 of representative wound tissues after 14 days of treatment (scale bar = 100 μm). **Fig. S22** Expression of SIRT1 and autophagy pathway proteins in diabetic wound tissues. **Fig. S23** Schematic diagram of anti-inflammatory and antioxidant properties of OLUE hydrogel. **Table S1** Primer sequences used for RT-PCR. **Table S2** Trace element species and concentrations in cell culture medium. **Table S3** Mathematical models of the regression for in vitro release profiles of preparations (*R*^2^).

## Data Availability

The authors confirm that the data supporting the findings of this study are available within the article and its Additional file 1.

## Abbreviations

ATP: Adenosine triphosphate
BCA: Bicinchoninic acid
CCK-8: Cell counting kit-8
CD206: Mannose receptor
CD3: Cluster of differentiation 3
CD11c: Integrin subunit alpha X
CD31: Platelet endothelial cell adhesion molecule-1
CD63: Cluster of differentiation 63
CD86: Cluster of differentiation 86
CD9: Cluster of differentiation 9
CLSM: Confocal laser scanning microscope
C1QBP: Complement 1q binding protein
CS: Chitosan
DAPI: 4',6-Diamidino-2-phenylindole
DC: Dendritic cells
DCFH-DA: 2',7'-Dichlorodihydrofluorescein diacetate
DFU: Diabetic foot ulcers
DLS: Dynamic light scattering
DMEM: Dulbecco’s modified Eagle’s medium
3D-Exo: Exosome derived from standard medium
DSC: Differential scanning calorimetry
2D: Two-dimensional
3D: Three-dimensional
3D-TE-Exo: Exosome derived from trace element-supplemented medium
EDC: Ethyldimethylaminopropyl carbodiimide
EDS: Energy dispersive spectroscopy
EdU: 5-Ethynyl-2'-deoxyuridine
ELISA: Enzyme-linked immunosorbent assay
Exo: Exosomes
FBS: Fetal bovine serum
FT-IR: Fourier transform infrared
G′: Storage modulus
G″: Loss modulus
GSH: Glutathione
Glu: Glucose
HA: Hyaluronic acid
HaCaT: Human immortal keratinocyte line
H&E staining: Hematoxylin-eosin staining
HLA-DR: Human leukocyte antigen d-related
^1^H NMR: Nuclear magnetic resonance hydrogen spectroscopy
H_2_O_2_: Hydrogen peroxide
HUVECs: Human umbilical vein endothelial cells
ICP-MS: Inductively coupled plasma mass spectrometry
IL-1β: Interleukin-1β
IL-6: Interleukin-6
IL-10: Interleukin-10
Ki-67: Kiel-67
LACS: Lipoic acid-grafted chitosan
LA: Lipoic acid
LC3: Microtubule-associated protein light chain 3
LPS: Lipopolysaccharide
MDA: Malondialdehyde
Mo-Exo: Mo-inspired macrophage-derived exosomes
MSCs: Mesenchymal stem cells
NHS: N-hydroxysuccinimide
NTA: Nanoparticle tracking analysis
O_2_: Oxygen
OHA: Oxidized hyaluronic acid
OHA-LACS: Hydrogels constructed from oxidized hyaluronic acid and lipoic acid-grafted chitosan
OHA-LACS-UV: Hydrogels constructed from oxidized hyaluronic acid and lipoic acid-grafted chitosan by ultraviolet light
OLUE: Ultraviolet light-irradiated oxidized hyaluronic acid and lipoic acid-grafted chitosan constructed hydrogel for exosomes
PBS: Phosphate buffered saline
p62: Sequestosome 1
RAW264.7: Mouse monocytic macrophage leukemia cells
ROS: Reactive oxygen species
RT-PCR: Reverse transcription-polymerase chain reaction
SEM: Scanning electron microscopy
SOD: Superoxide dismutase
SIRT1: Silent information regulator 1
STZ: Streptozocin
TEM: Transmission electron microscopy
TE: Trace elements
TGA: Thermogravimetric analysis
TGF-β: Transforming growth factor-β
TNF-α: Tumour necrosis factor-α
TSG101: Tumor susceptibility gene 101
VEGF: Vascular endothelial growth factor
XPS: X-ray photoelectron spectroscopy
XRD: X-ray diffraction

## References

[1] Ong KL, Stafford LK, Mclaughlin SA, Boyko EJ, Vollset SE, Smith AE, et al. Global, regional, and national burden of diabetes from 1990 to 2021, with projections of prevalence to 2050: a systematic analysis for the Global Burden of Disease Study 2021. Lancet. 2023;402(10397):203–34. 37356446 10.1016/S0140-6736(23)01301-6 PMC10364581

[2] Armstrong DG, Tan TW, Boulton AJM, Bus SA. Diabetic foot ulcers: a review. JAMA. 2023;330(1):62–75. 37395769 10.1001/jama.2023.10578 PMC10723802

[3] Armstrong DG, Swerdlow MA, Armstrong AA, Conte MS, Padula WV, Bus SA. Five year mortality and direct costs of care for people with diabetic foot complications are comparable to cancer. J Foot Ankle Res. 2020;13(1):16. 32209136 10.1186/s13047-020-00383-2 PMC7092527

[4] Theocharidis G, Thomas BE, Sarkar D, Mumme HL, Pilcher WJR, Dwivedi B, et al. Single cell transcriptomic landscape of diabetic foot ulcers. Nat Commun. 2022;13(1):181. 35013299 10.1038/s41467-021-27801-8 PMC8748704

[5] Mizelle RM Jr. Diabetes, race, and amputations. Lancet. 2021;397(10281):1256–7. 33812484 10.1016/S0140-6736(21)00724-8

[6] Godavari A, Moorthi M, Rajasekar A. Review on anti-diabetic research on two important spices: *Trachyspermum ammi* and *Pimpinella anisum*. Bio Integr. 2023;4(3):132.

[7] Bandyk DF. The diabetic foot: pathophysiology, evaluation, and treatment. Semin Vasc Surg. 2018;31(2):43–8. 30876640 10.1053/j.semvascsurg.2019.02.001

[8] Chen Y, Wang X, Tao S, Wang Q, Ma PQ, Li ZB, et al. Research advances in smart responsive-hydrogel dressings with potential clinical diabetic wound healing properties. Mil Med Res. 2023;10(1):37. 37608335 10.1186/s40779-023-00473-9 PMC10463485

[9] Edmonds M, Manu C, Vas P. The current burden of diabetic foot disease. J Clin Orthop Trauma. 2021;17:88–93. 33680841 10.1016/j.jcot.2021.01.017 PMC7919962

[10] Hetta HF, Elsaghir A, Sijercic VC, Akhtar MS, Gad SA, Moses A, et al. Mesenchymal stem cell therapy in diabetic foot ulcer: an updated comprehensive review. Health Sci Rep. 2024;7(4):e2036. 38650719 10.1002/hsr2.2036 PMC11033295

[11] Park KS, Lässer C, Lötvall J. Extracellular vesicles and the lung: from disease pathogenesis to biomarkers and treatments. Physiol Rev. 2025;105(3):1733–821. 40125970 10.1152/physrev.00032.2024

[12] Kumar MA, Baba SK, Sadida HQ, Marzooqi SA, Jerobin J, Altemani FH, et al. Extracellular vesicles as tools and targets in therapy for diseases. Signal Transduct Target Ther. 2024;9(1):27. 38311623 10.1038/s41392-024-01735-1 PMC10838959

[13] Yang Y, Zhang J, Wu S, Deng Y, Wang S, Xie L, et al. Exosome/antimicrobial peptide laden hydrogel wound dressings promote scarless wound healing through miR-21-5p-mediated multiple functions. Biomaterials. 2024;308:122558. 38581764 10.1016/j.biomaterials.2024.122558

[14] Yoo D, Jung SY, Go D, Park JY, You DG, Jung WK, et al. Functionalized extracellular vesicles of mesenchymal stem cells for regenerative medicine. J Nanobiotechnol. 2025;23(1):219. 10.1186/s12951-025-03300-6 PMC11921732 40102934

[15] Fan MH, Pi JK, Zou CY, Jiang YL, Li QJ, Zhang XZ, et al. HydroHydrogel-exosome system in tissue engineering: a promising therapeutic strategy. Bioact Mater. 2024;38:1–30. 38699243 10.1016/j.bioactmat.2024.04.007 PMC11061651

[16] Zhu D, Hu Y, Kong X, Luo Y, Zhang Y, Wu Y, et al. Enhanced burn wound healing by controlled-release 3D ADMSC-derived exosome-loaded hyaluronan hydrogel. Regen Biomater. 2024;11:rbae035. 38628545 10.1093/rb/rbae035 PMC11018541

[17] Cao J, Wang B, Tang T, Lv L, Ding Z, Li Z, et al. Three-dimensional culture of MSCs produces exosomes with improved yield and enhanced therapeutic efficacy for cisplatin-induced acute kidney injury. Stem Cell Res Ther. 2020;11(1):206. 32460853 10.1186/s13287-020-01719-2 PMC7251891

[18] Chen W, Wu P, Jin C, Chen Y, Li C, Qian H. Advances in the application of extracellular vesicles derived from three-dimensional culture of stem cells. J Nanobiotechnol. 2024;22(1):215. 10.1186/s12951-024-02455-y PMC11064407 38693585

[19] Eguchi T, Sheta M, Fujii M, Calderwood SK. Cancer extracellular vesicles, tumoroid models, and tumor microenvironment. Semin Cancer Biol. 2022;86(Pt 1):112–26. 35032650 10.1016/j.semcancer.2022.01.003

[20] Lou S, Hu W, Wei P, He D, Fu P, Ding K, et al. Artificial nanovesicles derived from cells: a promising alternative to extracellular vesicles. ACS Appl Mater Interfaces. 2025;17(1):22–41. 39692623 10.1021/acsami.4c12567

[21] Jeong J, Park JK, Shin J, Jung I, Kim HW, Park A, et al. Inflammatory cytokine-primed MSC-derived extracellular vesicles ameliorate acute lung injury via enhanced immunomodulation and alveolar repair. Stem Cell Res Ther. 2025;16(1):450. 40846969 10.1186/s13287-025-04576-z PMC12374373

[22] Chen L, Shen Q, Liu Y, Zhang Y, Sun L, Ma X, et al. Homeostasis and metabolism of iron and other metal ions in neurodegenerative diseases. Signal Transduct Target Ther. 2025;10(1):31. 39894843 10.1038/s41392-024-02071-0 PMC11788444

[23] Kurian SJ, Baral T, Unnikrishnan MK, Benson R, Munisamy M, Saravu K, et al. The association between micronutrient levels and diabetic foot ulcer: a systematic review with meta-analysis. Front Endocrinol. 2023;14:1152854. 10.3389/fendo.2023.1152854 PMC10090454 37065742

[24] Reddy S, Anoop S, Jebasingh FK, Dasgupta R, Joseph M, Saravanan B, et al. Differentials in dietary intake of macro and micronutrients in patients with type 2 diabetes and foot ulcers: observations from a pilot study. Clin Nutr ESPEN. 2022;47:170–6. 35063197 10.1016/j.clnesp.2021.12.023

[25] Liu X, Wang W, Wang Y, Duan W, Liu C, Quan P, et al. Biochemical strategy-based hybrid hydrogel dressing-mediated in situ synthesis of selenoproteins for DFU immunity-microbiota homeostasis regulation. Biomaterials. 2025;317:123114. 39854881 10.1016/j.biomaterials.2025.123114

[26] Wei J, Zhang X, Sui B, Ding X, Li Y, Liu B, et al. Potassium-doped MnO_2_ nanoparticles reprogram neutrophil calcium signaling to accelerate healing of methicillin-resistant *Staphylococcus aureus*-infected diabetic wounds. ACS Nano. 2025;19(12):11807–22. 40100101 10.1021/acsnano.4c14057 PMC11966767

[27] Ge Y, Rong F, Lu Y, Wang Z, Liu J, Xu F, et al. Glucose oxidase driven hydrogen sulfide-releasing nanocascade for diabetic infection treatment. Nano Lett. 2023;23(14):6610–8. 37458704 10.1021/acs.nanolett.3c01771

[28] Wu Z, He D, Li H. Bioglass enhances the production of exosomes and improves their capability of promoting vascularization. Bioact Mater. 2021;6(3):823–35. 33024902 10.1016/j.bioactmat.2020.09.011 PMC7530219

[29] Wang Z, Zhao Y, Zhao Y, Zhang Y, Yao X, Hang R. Exosomes secreted by macrophages upon copper ion stimulation can promote angiogenesis. Mater Sci Eng C Mater Biol Appl. 2021;123:111981. 33812609 10.1016/j.msec.2021.111981

[30] Yuan W, Liu J, Zhang Z, Ye C, Zhou X, Yi Y, et al. Strontium-alix interaction enhances exosomal miRNA selectively loading in synovial MSCs for temporomandibular joint osteoarthritis treatment. Int J Oral Sci. 2025;17(1):6. 39890774 10.1038/s41368-024-00329-5 PMC11785994

[31] Deng Y, Xie J, Xiao J, Huang X, Cao Z. Gelatin methacryloyl hydrogel encapsulating molybdenum-inspired macrophage-derived exosomes accelerates wound healing via immune regulation and angiogenesis. Int J Biol Macromol. 2025;291:138947. 39725118 10.1016/j.ijbiomac.2024.138947

[32] Wang S, Yin J, Liu Y, Jin M, Wang Q, Guo J, et al. An organic state trace element solution for rheumatoid arthritis treatment by modulating macrophage phenotypic from M1 to M2. Biomed Pharmacother. 2024;170:116025. 38113625 10.1016/j.biopha.2023.116025

[33] Piao Y, Wang N, Jin M, Piao J, Han M, Wang Z, et al. Multi-trace elements-enriched functional drink accelerates gastric ulcer repair via the HGF/c-Met/STAT3 pathway. J Funct Foods. 2025;125:106674.

[34] Jin W, Li Y, Yu M, Ren D, Han C, Guo S. Advances of exosomes in diabetic wound healing. Burns Trauma. 2025;13:tkae078. 39980588 10.1093/burnst/tkae078 PMC11836438

[35] Simon L, Lapinte V, Morille M. Exploring the role of polymers to overcome ongoing challenges in the field of extracellular vesicles. J Extracell Vesicles. 2023;12(12):e12386. 38050832 10.1002/jev2.12386 PMC10696644

[36] Lu P, Ruan D, Huang M, Tian M, Zhu K, Gan Z, et al. Harnessing the potential of hydrogels for advanced therapeutic applications: current achievements and future directions. Signal Transduct Target Ther. 2024;9(1):166. 38945949 10.1038/s41392-024-01852-x PMC11214942

[37] Teng X, Liu T, Zhao G, Liang Y, Li P, Li F, et al. A novel exosome-based multifunctional nanocomposite platform driven by photothermal-controlled release system for repair of skin injury. J Control Release. 2024;371:258–72. 38815704 10.1016/j.jconrel.2024.05.049

[38] Cheng N, Luo Q, Yang Y, Shao N, Nie T, Deng X, et al. Injectable pH responsive conductive hydrogel for intelligent delivery of metformin and exosomes to enhance cardiac repair after myocardial ischemia-reperfusion injury. Adv Sci. 2025;12(24):e2410590. 10.1002/advs.202410590 PMC12199616 39965141

[39] Guo L, Fu Z, Li H, Wei R, Guo J, Wang H, et al. Smart hydrogel: a new platform for cancer therapy. Adv Colloid Interface Sci. 2025;340:103470. 40086017 10.1016/j.cis.2025.103470

[40] Wang S, Liu Y, Sun Q, Zeng B, Liu C, Gong L, et al. Triple cross-linked dynamic responsive hydrogel loaded with selenium nanoparticles for modulating the inflammatory microenvironment via PI3K/Akt/NF-κB and MAPK signaling pathways. Adv Sci. 2023;10(31):e2303167. 10.1002/advs.202303167 PMC10625091 37740428

[41] Wang S, Liu Y, Wang X, Chen L, Huang W, Xiong T, et al. Modulating macrophage phenotype for accelerated wound healing with chlorogenic acid-loaded nanocomposite hydrogel. J Control Release. 2024;369:420–43. 38575075 10.1016/j.jconrel.2024.03.054

[42] Wang S, Jiang L, Meng S, Liu C, Wang H, Gao Z, et al. Hollow mesoporous silica nanoparticles-loaded ion-crosslinked bilayer films with excellent mechanical properties and high bioavailability for buccal delivery. Int J Pharm. 2022;624:122056. 35905934 10.1016/j.ijpharm.2022.122056

[43] Zhao L, Zhou Y, Zhang J, Liang H, Chen X, Tan H. Natural polymer-based hydrogels: from polymer to biomedical applications. Pharmaceutics. 2023;15(10):2514. 37896274 10.3390/pharmaceutics15102514 PMC10610124

[44] Yang P, Ju Y, Shen N, Zhu S, He J, Yang L, et al. Exos-loaded gox-modified smart-response self-healing hydrogel improves the microenvironment and promotes wound healing in diabetic wounds. Adv Healthc Mater. 2025;14(7):e2403304. 39473310 10.1002/adhm.202403304

[45] Wang Z, Qing H, Li R, Li X, Guo X, Zhou S. M2 macrophage-derived exosomes inhibiting neutrophil extracellular traps for ischemic stroke therapy. Adv Funct Mater. 2024;34(42):2402724.

[46] Fan MH, Zhang XZ, Jiang YL, Pi JK, Zhang JY, Zhang YQ, et al. Exosomes from hypoxic urine-derived stem cells facilitate healing of diabetic wound by targeting SERPINE1 through miR-486-5p. Biomaterials. 2025;314:122893. 39418849 10.1016/j.biomaterials.2024.122893

[47] Kostyusheva A, Romano E, Yan N, Lopus M, Zamyatnin AA Jr., Parodi A. Breaking barriers in targeted therapy: advancing exosome isolation, engineering, and imaging. Adv Drug Deliv Rev. 2025;218:115522. 39855273 10.1016/j.addr.2025.115522

[48] Zheng Z, Chen X, Wang Y, Wen P, Duan Q, Zhang P, et al. Self-growing hydrogel bioadhesives for chronic wound management. Adv Mater. 2024;36(41):e2408538. 39149779 10.1002/adma.202408538

[49] Zhu W, Wang H, Feng B, Liu G, Bian Y, Zhao T, et al. Self-healing hyaluronic acid-based hydrogel with miRNA140-5p loaded MON-PEI nanoparticles for chondrocyte regeneration: schiff base self-assembly approach. Adv Sci. 2025;12(1):e2406479. 10.1002/advs.202406479 PMC11714154 39498998

[50] Park J, Wu Y, Suk Kim J, Byun J, Lee J, Oh YK. Cytoskeleton-modulating nanomaterials and their therapeutic potentials. Adv Drug Deliv Rev. 2024;211:115362. 38906478 10.1016/j.addr.2024.115362

[51] Zhang SJ, Xu R, He SB, Sun R, Wang GN, Wei SY, et al. Nanozyme-driven multifunctional dressings: moving beyond enzyme-like catalysis in chronic wound treatment. Mil Med Res. 2025;12(1):27. 40448212 10.1186/s40779-025-00611-5 PMC12125814

[52] Song J, Zhao T, Wang C, Sun X, Sun J, Zhang Z. Cell migration in diabetic wound healing: molecular mechanisms and therapeutic strategies (review). Int J Mol Med. 2025;56(2):126. 40539458 10.3892/ijmm.2025.5567 PMC12180913

[53] Saadh MJ, Allela OQB, Kareem RA, Baldaniya L, Roopashree R, Thakur V, et al. Harnessing exosomal mediators for advanced wound healing: mechanisms and therapeutic potential in angiogenesis. Microvasc Res. 2025;162:104861. 40848894 10.1016/j.mvr.2025.104861

[54] Dawes JS, Abdelaal M, Landmesser ME, Asgardoon MH, Waldron OP, Park JH, et al. Exosomes: the future of acellular nanotherapeutics in regenerative vascularization. Front Bioeng Biotechnol. 2025;13:1607605. 40979643 10.3389/fbioe.2025.1607605 PMC12443856

[55] Lee C, Kim MJ, Kumar A, Lee HW, Yang Y, Kim Y. Vascular endothelial growth factor signaling in health and disease: from molecular mechanisms to therapeutic perspectives. Signal Transduct Target Ther. 2025;10(1):170. 40383803 10.1038/s41392-025-02249-0 PMC12086256

[56] Luo X, Zhang Y, Zeng Y, Yang D, Zhou Z, Zheng Z, et al. Nanotherapies based on ROS regulation in oral diseases. Adv Sci. 2025;12(9):e2409087. 10.1002/advs.202409087 PMC11884622 39887942

[57] Li M, Wu L, Si H, Wu Y, Liu Y, Zeng Y, et al. Engineered mitochondria in diseases: mechanisms, strategies, and applications. Signal Transduct Target Ther. 2025;10(1):71. 40025039 10.1038/s41392-024-02081-y PMC11873319

[58] Dawi J, Tumanyan K, Tomas K, Misakyan Y, Gargaloyan A, Gonzalez E, et al. Diabetic foot ulcers: pathophysiology, immune dysregulation, and emerging therapeutic strategies. Biomedicines. 2025;13(5):1076. 40426903 10.3390/biomedicines13051076 PMC12109115

[59] Slatter DA, Bolton CH, Bailey AJ. The importance of lipid-derived malondialdehyde in diabetes mellitus. Diabetologia. 2000;43(5):550–7. 10855528 10.1007/s001250051342

[60] Zhang J, Li L, Yu J, Zhang F, Shi J, Li M, et al. Autophagy-modulated biomaterial: a robust weapon for modulating the wound environment to promote skin wound healing. Int J Nanomedicine. 2023;18:2567–88. 37213350 10.2147/IJN.S398107 PMC10198186

[61] Saraiva M, Vieira P, O’garra A. Biology and therapeutic potential of interleukin-10. J Exp Med. 2020;217(1):e20190418. 31611251 10.1084/jem.20190418 PMC7037253

[62] Yu L, Wen H, Liu C, Wang C, Yu H, Zhang K, et al. Embryonic stem cell-derived extracellular vesicles rejuvenate senescent cells and antagonize aging in mice. Bioact Mater. 2023;29:85–97. 37449253 10.1016/j.bioactmat.2023.06.011 PMC10336196

[63] Gutiérrez-Aguilar M, Klutho PJ, Aguayo-Ortiz R, Song L, Baines CP. Endogenous complement 1q binding protein (C1qbp) regulates mitochondrial permeability transition and post-myocardial infarction remodeling and dysfunction. J Mol Cell Cardiol. 2024;196:1–11. 39209214 10.1016/j.yjmcc.2024.08.005 PMC11534557

[64] Bai Y, Wang W, Li S, Zhan J, Li H, Zhao M, et al. C1QBP Promotes homologous recombination by stabilizing MRE11 and controlling the assembly and activation of MRE11/RAD50/NBS1 complex. Mol Cell. 2019;75(6):1299-314.e6. 31353207 10.1016/j.molcel.2019.06.023

[65] Kim N, Lee H, Han G, Kang M, Park S, Kim DE, et al. 3D-printed functional hydrogel by DNA-induced biomineralization for accelerated diabetic wound healing. Adv Sci. 2023;10(17):e2300816. 10.1002/advs.202300816 PMC10265106 37076933

[66] Bairagi VA, Jagtap SD, Jadhav SB, Jadhav SR, Ahire YS. Rutin and glimepiride combination: a novel approach for managing diabetes nephropathy in streptozotocin-induced diabetes in Wistar rats. Bio Integr. 2024;5(1):963.

[67] Xie H, Wang Z, Wang R, Chen Q, Yu A, Lu A. Self-healing, injectable hydrogel dressing for monitoring and therapy of diabetic wound. Adv Funct Mater. 2024;34(36):2401209.

[68] Deng Z, Fan T, Xiao C, Tian H, Zheng Y, Li C, et al. TGF-β signaling in health, disease, and therapeutics. Signal Transduct Target Ther. 2024;9(1):61. 38514615 10.1038/s41392-024-01764-w PMC10958066

[69] Chen B, Yu P, Chan WN, Xie F, Zhang Y, Liang L, et al. Cellular zinc metabolism and zinc signaling: from biological functions to diseases and therapeutic targets. Signal Transduct Target Ther. 2024;9(1):6. 38169461 10.1038/s41392-023-01679-y PMC10761908

[70] Ru Q, Li Y, Chen L, Wu Y, Min J, Wang F. Iron homeostasis and ferroptosis in human diseases: mechanisms and therapeutic prospects. Signal Transduct Target Ther. 2024;9(1):271. 39396974 10.1038/s41392-024-01969-z PMC11486532

[71] Lima FDS, Santos MQD, Makiyama EN, Hoffmann C, Fock RA. The essential role of magnesium in immunity and gut health: impacts of dietary magnesium restriction on peritoneal cells and intestinal microbiome. J Trace Elem Med Biol. 2025;88:127604. 39884252 10.1016/j.jtemb.2025.127604

[72] Fekete M, Lehoczki A, Csípő T, Fazekas-Pongor V, Szappanos Á, Major D, et al. The role of trace elements in COPD: pathogenetic mechanisms and therapeutic potential of zinc, iron, magnesium, selenium, manganese, copper, and calcium. Nutrients. 2024;16(23):4118. 39683514 10.3390/nu16234118 PMC11644833

[73] Huang H, Pan W, Wang Y, Kim HS, Shao D, Huang B, et al. Nanoparticulate cell-free DNA scavenger for treating inflammatory bone loss in periodontitis. Nat Commun. 2022;13(1):5925. 36207325 10.1038/s41467-022-33492-6 PMC9546917

[74] Becker YLC, Gagné JP, Julien AS, Lévesque T, Allaeys I, Gougeard N, et al. Identification of mitofusin 1 and complement component 1q subcomponent binding protein as mitochondrial targets in systemic lupus erythematosus. Arthritis Rheumatol. 2022;74(7):1193–203. 35128841 10.1002/art.42082

[75] Tian H, Chai D, Wang G, Wang Q, Sun N, Jiang G, et al. Mitochondrial C1QBP is essential for T cell antitumor function by maintaining mitochondrial plasticity and metabolic fitness. Cancer Immunol Immunother. 2023;72(7):2151–68. 36828964 10.1007/s00262-023-03407-5 PMC10992850

[76] Cheng BJ, Wang J, Meng XL, Sun L, Hu B, Li HB, et al. The association between essential trace element mixture and cognitive function in Chinese community-dwelling older adults. Ecotoxicol Environ Saf. 2022;231:113182. 35026581 10.1016/j.ecoenv.2022.113182

[77] Mao Y, Zhao B, Xu L, Wang Y, Qiu X, Sun Y, et al. Protein-polysaccharide based nanogel/hydrogel composite with controlled continuous delivery of drug for enhanced wound healing. Carbohydr Polym. 2025;356:123407. 40049977 10.1016/j.carbpol.2025.123407

[78] Li L, Wang Y, Hu S, Chang X, Ding Q, Wang K, et al. Peroxidase-like copper-doped carbon-dots embedded in hydrogels for stimuli-responsive bacterial biofilm elimination and wound healing. Acta Biomater. 2025;195:467–78. 39938706 10.1016/j.actbio.2025.02.022

