# Supplementary material for: Trace element-dictated exosome modules and self-adaptive dual-network hydrogel orchestrate diabetic foot regeneration through complement-mitochondria-autophagy circuitry
Source: Mil Med Res. 2025 Oct 28;12:71. doi: 10.1186/s40779-025-00658-4 (PMC12560296; doi:10.1186/s40779-025-00658-4)
Supplement: Supplementary file 1 — Additional file 1. Methods. Fig. S1 Identification and evaluation of MSCs. Fig. S2 Zeta potentials of 3D-Exo and 3D-TE-Exo detected by DLS. Fig. S3 Synthesis and identification of OHA. Fig. S4 Synthesis and identification of LACS. Fig. S5 Simultaneous thermal analysis of HA, OHA, CS, LA, LACS, OHA-LACS, and OHA-LACS-UV. Fig. S6 The XPS spectra of OHA-LACS hydrogel, including C, O, N, and S. Fig. S7 The swelling rate of OHA-LACS and OHA-LACS-UV hydrogels (n = 3). Fig. S8 Biocompatibility of OLUE hydrogel. Fig. S9 Hemolytic properties of OHA-LACS-UV hydrogels. Fig. S10 Quantification of ROS fluorescence intensity by ImageJ software HUVECs and HaCaT cells (n = 3). Fig. S11 Neutral red cell proliferation test (n = 6). Fig. S12 ATP in the mitochondria of HUVECs and HaCaT cells (n = 6). Fig. S13 Quantification of Western blotting results. Fig. S14 Expression and identification of C1QBP in 3D-TE-Exo. Fig. S15 Expression and identification of C1QBP in MSCs and TE-MSCs. Fig. S16 Silence and functional evaluation of C1QBP. Fig. S17 In vivo retention and safety properties of OLUE hydrogel. Fig. S18 In vivo hemostasis and coagulation properties of OLUE hydrogel. Fig. S19 In vivo evaluation of OLUE hydrogel in the healthy rats full-thickness wound. Fig. S20 Quantification of α-SMA fluorescence intensity by ImageJ software (n = 3). Fig. S21 Immunohistochemistry staining for IL-6 and IL-10 of representative wound tissues after 14 days of treatment (scale bar = 100 μm). Fig. S22 Expression of SIRT1 and autophagy pathway proteins in diabetic wound tissues. Fig. S23 Schematic diagram of anti-inflammatory and antioxidant properties of OLUE hydrogel. Table S1 Primer sequences used for RT-PCR. Table S2 Trace element species and concentrations in cell culture medium. Table S3 Mathematical models of the regression for in vitro release profiles of preparations (R2). [file 40779_2025_658_MOESM1_ESM.pdf]

# Methods

## Polymer synthesis and characterization

### *Preparation and identification of oxidized hyaluronic acid (OHA)*

The synthesis of OHA was performed via sodium periodate ($NaIO_4$) oxidation. Briefly, 1.25 ml of 214.4 mg/ml $NaIO_4$ solution was added dropwise (0.5 ml/min) into 50 ml of 10 mg/ml hyaluronic acid (HA) solution under continuous stirring (600 r/min) at 25 °C in a light-protected reactor. After 3.5 h, the reaction was quenched by adding 2 ml ethylene glycol to chelate residual $NaIO_4$, followed by 15-minute stirring. The mixture was then dialyzed against deionized water using 3.5 kD MWCO regenerated cellulose membranes at 4 °C for 72 h. The purified product was lyophilized to yield white porous OHA powder.

The aldehyde group content of OHA was quantified via hydroxylamine hydrochloride titration. Briefly, 20 mg OHA was dissolved in 10 ml hydroxylamine hydrochloride-methyl orange solution and stirred at 25 °C for 4 h to facilitate oxime formation. Unmodified HA served as the blank control. The mixture was titrated with 0.05 mol/L NaOH until the solution color transitioned from red to yellow (methyl orange endpoint). The oxidation degree (DO) was calculated. The structure of OHA and HA was analyzed by the KBr pellet method. The spectra were recorded in the frequency range of 4000 to 400 $cm^{-1}$. Hydrogen atom types in OHA and HA were characterized by nuclear magnetic resonance hydrogen spectroscopy ($^1H$ NMR). The solvent is deuterated heavy water.

### *Synthesis of lipoic acid-grafted chitosan (LACS)*

Disperse 0.8 g of chitosan (CS) in 40 ml of 1.5% (v/v) acetic acid solution to prepare a CS solution. After stirring continuously for 1 h, add 20 ml of an ethanol solution containing 0.08 g lipoic acid (LA) to the CS solution until a homogeneous mixture is obtained. Subsequently, add 0.534 g of ethyldimethylaminopropyl carbodiimide (EDC) and 0.1334 g of N-hydroxysuccinimide (NHS) to the mixture, and allow the reaction to proceed for 4 h. Finally, dialyze the reaction mixture for 3 d using dialysis tubing and lyophilize the product for further use.

## Construction and evaluation of hydrogels

### *Construction of hydrogels*

A predetermined amount of OHA was dissolved in pH = 7.4 phosphate-buffered saline (PBS) to

prepare a 2% (w/v) OHA solution; simultaneously, a 2% (w/v) LACS solution was prepared by dissolving an appropriate amount of LACS in pH = 7.4 PBS. The 2 solutions, having the same concentration, were then mixed in equal volumes to form the OHA-LACS hydrogel. Subsequently, the mixture was exposed to ultraviolet (UV) irradiation (365 nm, 10 mW $cm^{-2}$) for secondary crosslinking, resulting in a hydrogel designated as OHA-LACS-UV, with the gelation time duly recorded. 3D-TE-Exo-loaded hydrogel was further constructed. Specifically, 3D-TE-Exo (at a concentration of 1% w/v) was uniformly dispersed into the OHA solution, followed by mixing with the LACS solution, and finally, UV irradiation was applied to solidify the mixture, yielding a hydrogel referred to as OHA-LACS-UV-3D-TE-Exo (OLUE).

***Physicochemical testing of hydrogels***

To comprehensively evaluate the chemical properties and physical morphology of the hydrogel, a variety of characterization techniques were employed. Firstly, changes in the functional groups of the hydrogel were analyzed using Fourier transform infrared (FT-IR) on a Nicolet ls10 instrument (manufactured in the USA), which enabled the investigation of the formation and breaking of chemical bonds. Secondly, the crystalline structure and morphology of the hydrogel were determined using X-ray diffraction (XRD) on a Bruker D8 Advance instrument (manufactured in Germany) over an angular range of $2\theta = 3° – 50°$. In parallel, the microstructure of the hydrogel was examined using scanning electron microscopy (SEM) with a Thermo Scientific Apreo S (manufactured in the USA), while its elemental composition was analyzed via energy dispersive spectroscopy (EDS) on an Oxford Max-65 system (manufactured in the UK). Finally, the thermal stability and transition properties of the hydrogel were assessed through thermogravimetric analysis (TGA) and differential scanning calorimetry (DSC) using a Mettler TG-DSC 3+ and a TA DSC25 (manufactured in Switzerland), respectively.

***Rheological properties***

To comprehensively evaluate the rheological properties of the hydrogel, a systematic analysis was conducted using a Kinexus Lab+ rheometer (Marvin, UK). At 37 °C, the hydrogel's storage modulus (G′) and loss modulus (G″) were measured. Ensuring operation within the linear viscoelastic region, a constant strain of 0.5% was applied while performing a frequency sweep over the range of 0.01 to 10 Hz. Additionally, strain sweep tests were conducted, varying the strain from a low value ($\gamma = 0.1\%$) to a high value ($\gamma = 300\%$), to assess the material's response under different deformations. Furthermore, the self-healing properties of the hydrogel were evaluated by applying alternating strain cycles at high

and low strain (1 – 500)% cyclic scanning. To further investigate the flow behavior under various shear conditions, viscosity tests were performed over a shear rate range of $\theta$'(t) = 0.05 – 100 rad/s, thereby elucidating the hydrogel's flow characteristics and its response to external stresses.

***Self-healing, injectability, adhesion, and tensile properties***

To assess the self-healing ability of the OHA-LACS-UV hydrogel, the material was first cut into two pieces using scissors, then the separated pieces were reassembled into a new shape and allowed to heal at 25 °C for 5 min. To verify the injectability, the OHA-LACS hydrogel was loaded into a 26G syringe and extruded to write the word "PUMC" on a substrate; furthermore, the hydrogel was injected into a perforated bone model to demonstrate its capacity to conform to complex surfaces.

At room temperature, the adhesion performance of the OHA-LACS-UV hydrogel was evaluated on both biological tissues and common materials. In this test, the hydrogel was pre-applied on a finger and then brought into contact with major tissues such as the rat heart, liver, spleen, lung, and kidney. Additionally, its adhesion was assessed on common materials, including polyethylene plastic, steel, glass, and paper. Visual observation and photographic documentation were employed to systematically evaluate the adhesive properties of the hydrogel on various substrates.

To evaluate the tensile strength and stretchability, the OHA-LACS-UV hydrogel was applied to moist porcine skin, which was then repeatedly folded and bent. Similarly, the hydrogel was applied to a finger and subjected to continuous bending to observe any occurrence of cracking or separation. These experiments validated the hydrogel's mechanical stability and flexibility under dynamic strain.

***In vitro swelling and degradation***

In vitro swelling and degradation tests were performed to evaluate the hydration capability and stability of the hydrogels. For the swelling experiments, hydrogels (OHA-LACS or OHA-LACS-UV) were immersed in pH = 7.4 PBS at 37 °C. At predetermined time points, the swollen hydrogels were removed, and excess surface water was gently blotted with filter paper; the weight of the hydrogel at this time was recorded as $W_t$, while the initial weight was recorded as $M_0$. The swelling percentage was calculated using the formula: Swelling (%) = $(W_t – M_0)/ M_0 \times 100\%$.

For the in vitro degradation experiments, the hydrogels were incubated in different solutions: pH = 7.4 PBS, 1 mmol/L hydrogen peroxide ($H_2O_2$), 3 mg/ml glucose (Glu), and a combination of 1 mmol/L $H_2O_2$ with 3 mg/ml glucose ($H_2O_2$+ Glu). All samples were maintained at 25 °C for 16 d. At predetermined intervals, the hydrogels were removed, freeze-dried, and the remaining weight was

recorded as $M_d$. The degradation percentage was calculated using the formula: Degradation (%) = ($M_0$ – $M_d$)/ $M_0$ × 100%. Three parallel samples were used for each condition

***In vitro release***

To evaluate the release characteristics of Exo from the OLUE hydrogel, the hydrogel was immersed in various release media, including pH = 7.4 PBS, 1 mmol/L $H_2O_2$, 3 mg/ml Glu, and $H_2O_2$ + Glu. All experiments were conducted at 37 °C with a stirring rate of 50 r/min. At predetermined time intervals, the release medium was collected and replaced with an equal volume of fresh solution to maintain a constant volume. The absorbance of the collected release media was measured at 595 nm using the bicinchoninic acid (BCA) assay, and the amount of Exo released was quantified based on a predetermined calibration curve. This method provided a systematic evaluation of the in vitro release profile of Exo from the OLUE hydrogel under various conditions.

**Cell biocompatibility**

***Concentration screening for Exo***

To evaluate the effect of different Exo concentrations on cell proliferation, a cell counting kit-8 (CCK-8) assay was employed. Specifically, human umbilical vein endothelial cells (HUVECs) and human immortal keratinocyte line (HaCaT) cells were seeded into 96-well plates at a density of 5 × $10^3$ cells per well. After allowing the cells to adhere and grow, various concentrations of either 3D-TE-Exo or 3D-Exo were added to the wells, and the cells were co-incubated for 1 or 3 d. After the incubation period, the culture medium was removed and replaced with 100 μl of serum-free medium containing 10% (v/v) CCK-8 reagent. Following further incubation, the absorbance of each well was measured at 490 nm using a microplate reader ($n$ = 6). Wells without any Exo treatment served as the control group, enabling the assessment of the proliferation-promoting effects of different Exo concentrations.

***Safety of hydrogels***

To evaluate the biocompatibility of hydrogel extracts at various concentrations, in vitro tests were performed using HUVECs and HaCaT cells. Specifically, HUVECs and HaCaT cells were seeded into 96-well plates at a density of 5 × $10^3$ cells per well. After allowing the cells to adhere, hydrogel extracts of different concentrations were added, and the cells were incubated for 1 and 3 d. After incubation, the culture medium was removed and replaced with 100 μl of serum-free medium containing 10% (v/v) CCK-8 reagent, followed by further incubation. Finally, the absorbance of each well was measured at

490 nm using a microplate reader ($n$ = 6), with wells that did not receive any hydrogel extract serving as the control group, thereby assessing the biocompatibility of the hydrogels.

***Hemolytic***

To evaluate the hemolytic activity of the hydrogels at various concentrations, fresh rat blood was first collected and centrifuged at 3000 r/min for 10 min to isolate red blood cells (RBC), which were subsequently washed repeatedly with pH = 7.4 PBS. Different concentrations of OHA-LACS-UV were then added to a 2% (v/v) RBC suspension and incubated at 37 °C for 2 h. PBS and Triton X-100 were used as negative and positive controls, respectively. Finally, the absorbance of the supernatant was measured at 540 nm using a microplate reader ($n$ = 3) to calculate the hemolysis rate.

***Cell viability***

Next, the effects of various formulations on cell proliferation were compared. Specifically, HUVECs and HaCaT cells were seeded in 96-well plates at a density of $5 \times 10^3$ cells per well. After allowing the cells to adhere, 200 μmol/L $H_2O_2$ was added to establish an inflammatory model, and the cells were incubated for 24 h. Subsequently, 3D-Exo, 3D-TE-Exo, and OLUE were added to the cells, which were then incubated for 1 and 3 d. Following incubation, the medium was replaced with 100 μl of serum-free medium containing 10% (v/v) CCK-8 reagent, and after further incubation, the absorbance was measured at 490 nm using a microplate reader ($n$ = 6). Wells without any formulation served as the control group, while those treated with $H_2O_2$ alone were designated as the model group.

***5-Ethynyl-2'-deoxyuridine (EdU) staining***

HUVECs and HaCaT cells were seeded in 12-well plates at $1 \times 10^5$ cells/well. After adhesion, oxidative stress was induced by 24-hour treatment with 200 μmol/L $H_2O_2$, followed by 24 h co-culture with experimental formulations. Cells were incubated with 50 μmol/L EdU in complete medium for 2 h. Cells were washed twice with PBS, fixed with 4% paraformaldehyde, and permeabilized with 0.5% Triton X-100. A Click reaction cocktail containing Azide 488 fluorophore, 1 mmol/L $CuSO_4$, and 100 mmol/L sodium ascorbate was added (0.5 ml/well) for 30-minute dark incubation. After PBS washing, nuclei were counterstained with 5 μg/ml Hoechst 33342 for 10 min. Fluorescent imaging was conducted via confocal laser scanning microscope (CLSM; Biotek, Winooski, VT, USA) with excitation/emission wavelengths set to 346/460 nm (Hoechst 33342, blue channel) and 495/519 nm (Azide 488, green channel). EdU positive cell ratios were quantified from five randomly selected fields

per group.

***Live/dead cell staining***

Finally, a live/dead cell staining assay was performed to further assess the effects of the different formulations on cell proliferation. Specifically, HUVECs and HaCaT cells were seeded into 12-well plates. After cell adhesion, 200 μmol/L $H_2O_2$ was added, and the cells were incubated for 24 h to establish an inflammatory model. Then, 3D-Exo, 3D-TE-Exo, and OHA-LACS-UV were added to the cells, which were incubated for 1 d and 3 d, respectively. Subsequently, the cells were stained with 500 μl of a calcein-AM/propidium iodide solution and further incubated at 37 °C for 30 min. The cells were washed three times with PBS, and cell morphology was observed using a CLSM. Wells without any formulation served as the control group, while those treated only with $H_2O_2$ were designated as the model group.

**Cell behavior**

***Cytoskeleton***

To observe the response of the actin cytoskeleton under inflammatory stimulation, immunofluorescence staining was performed. HUVECs and HaCaT cells were separately seeded into 12-well plates and cultured until reaching 50% confluence. Subsequently, 200 μmol/L $H_2O_2$ was added, and the cells were incubated for an additional 24 h to establish an inflammation model. Following this, 3D-Exo, 3D-TE-Exo, and OLUE were added to the inflammatory model, and the incubation continued for another day. After treatment, the culture medium was discarded, and the cells were fixed with 4% paraformaldehyde for 15 min. To increase membrane permeability, the cells were treated with 0.1% Triton X-100 for 10 min. Non-specific antigen sites were blocked using 5% bovine serum albumin, followed by incubation at room temperature for 30 min. Next, fluorescein isothiocyanate-labeled phalloidin working solution was added to stain F-actin, and the cells were incubated in the dark at room temperature for 30 min. After extensive washing with PBS to remove unbound dye, 10 nmol/L 4',6-diamidino-2-phenylindole (DAPI) staining solution was added to label the cell nuclei, with incubation at room temperature for 5 min. Finally, a CLSM was used to observe cell fluorescence.

***Cell scratch assay***

To simulate the skin wound microenvironment, a co-culture system comprising macrophages (RAW264.7), HUVECs, and HaCaT cells was established. Specifically, a suspension containing all

three types of cells at a ratio of RAW264.7: HUVECs: HaCaT = 1:10:10 was prepared and seeded into 6-well plates at a total density of 2 × $10^5$ cells per well. After 6 h of incubation, 200 μmol/L $H_2O_2$ was added, and the cells were incubated for another 24 h. A pipette tip was then used to create a scratch in the monolayer. The cells were incubated with different formulations, and images were captured at 0, 24, and 36 h to assess the scratch closure rate.

***Vertical migration assay***

HUVECs and HaCaT cells were seeded at a density of 1 × $10^5$ cells per well in the upper chamber of a Transwell (Costar 3422, US, 8.0 μm pore size), respectively. Different formulations were added to 200 μl of culture medium. After 12 h of incubation, the cells on the underside of the membrane were fixed with 4% paraformaldehyde. The cells were then stained with crystal violet for 30 min. After washing, the cells were observed under an inverted optical microscope.

***Angiogenesis assay***

Fifty microliters of Matrigel were added to pre-cooled 96-well plates and incubated for 1 h in a cell culture incubator. HUVECs were then seeded in the 96-well plates. The cells were incubated with 200 μl of different formulations for 6 h. Angiogenesis images were obtained using CLSM. Three random fields were selected from each well using ImageJ, and the total vessel length, number of connections, and branches were analyzed.

***Mechanism of angiogenesis***

HUVECs were seeded at a density of 2 × $10^5$ cells per well in a 12-well culture plate. An inflammation model was established by treating the HUVECs with $H_2O_2$, followed by incubation with different samples for 24 h. The cells were then fixed with 4% paraformaldehyde for 10 min, followed by permeabilization and blocking. The cells were incubated overnight at 4 °C with primary antibodies against vascular endothelial growth factor (VEGF; 1:200) or platelet endothelial cell adhesion molecule-1 (CD31; 1:200), and then with fluorescently labeled secondary antibodies for 1 h at room temperature. The nuclei were counterstained with DAPI for 10 min. Finally, immune-fluorescence images of the cells were captured using CLSM.

**Adenosine triphosphate (ATP) detection**

HUVECs and HaCaT cells were seeded at a density of 1 × $10^5$ cells per well in a 12-well plate to

establish an inflammation model. The cells were co-cultured with different formulations for 24 h. After removing the culture medium, 150 μl of lysis buffer was added to each well. The cells were lysed, and the lysates were centrifuged at 12,000× *g* for 5 min at 4 °C. The supernatant was collected for subsequent analysis. ATP detection working solution (100 μl) was added to the detection wells and incubated at room temperature for 5 min. Then, 20 μl of sample or standard was added to each well, mixed thoroughly, and ATP concentration was measured using a microplate reader.

**Neutral red uptake assay**

RAW264.7 cells were seeded at a density of $1 \times 10^5$ cells/ml in a 96-well plate, with 100 μl per well, and incubated for 6 h. The cells were then treated with 1 μg/ml lipopolysaccharide (LPS) for 24 h to induce inflammation. Afterward, the cells were co-cultured with different formulations for 24 h. Following incubation, 50 μl of 0.075% neutral red solution was added to each well. After 4 h, the neutral red was extracted from the cells, and absorbance at 540 nm was measured using a microplate reader

**Macrophage phenotype**

RAW264.7 cells were seeded in 6-well plates and incubated for 12 h to establish an inflammation model. The cells were then co-cultured with different formulations for 24 h. Following fixation with 4% paraformaldehyde, the cells were permeabilized and blocked. The cells were incubated overnight at 4 °C with primary antibodies targeting CD86 (1:400) and CD206 (1:500). After washing, the cells were incubated with fluorescence-conjugated secondary antibodies for 1 h. DAPI was used to stain the cell nuclei for 10 min. After PBS washing, immunofluorescence images were captured using a CLSM. FITC fluorescence was green, with an excitation wavelength of 490 nm and an emission wavelength of 525 nm. Cyanine 5 fluorescence was red, with an excitation wavelength of 649 nm and an emission wavelength of 670 nm.

***C1qbp* knockdown 3D-TE-Exo preparation**

When TE-MSCs reached 80% confluence, cell transfection was carried out using Lipofectamine 2000 (Invitrogen, USA). *C1qbp*-specific siRNA (CGGAGACAAAGCTTTTGTTGATT) or siNC was transfected into TE-MSCs following the manufacturer's instructions, and the cells were incubated for 24 h. Following transfection, 3D-Exo-*siNC*, 3D-TE-Exo-*siNC*, and 3D-TE-Exo-*siC1qbp* were prepared. Western blotting analysis was performed to confirm the knockdown efficiency.

**In vivo retention and safety**

Subcutaneous injection of 0.2 ml hydrogel was administered into the abdominal region of rats. Body weight was recorded daily throughout the experimental period, and photographic documentation was concurrently performed to monitor hydrogel degradation dynamics.

Following complete hydrogel degradation, rats were anesthetized, and 1 ml of blood was collected via the retro-orbital venous plexus using capillary tubes for hematological and biochemical analyses. Subsequently, euthanasia was performed, and subcutaneous tissue surrounding the injection site was excised and fixed in 4% paraformaldehyde. Histopathological evaluation of tissue integrity and inflammatory response was conducted via hematoxylin-eosin (H&E) staining. Major organs (heart, liver, spleen, lungs, and kidneys) were harvested for systemic toxicity assessment using H&E stained to detect potential pathological alterations.

**In vivo hemostatic studies**

The hemostatic efficacy of the hydrogel was evaluated using three distinct rat hemorrhage models: tail amputation, liver injury, and cardiac puncture. In the tail amputation model, rats were anesthetized with 2% isoflurane (5 min), followed by surgical transection 1 cm from the tail base. Immediately after amputation, 0.1 ml of hydrogel was applied to the bleeding site, and hemostatic time (defined as complete cessation of bleeding) was recorded. Blood loss was quantified by gravimetric analysis using pre-weighed filter paper. Untreated acute wounds served as controls. For liver injury, a midline laparotomy was performed to expose the liver. After removing surface fluids with sterile filter paper, a standardized incision (2 mm depth × 5 mm length) was created on the left hepatic lobe using a scalpel. Hydrogel (0.5 ml) was applied to the wound, and blood loss was measured via pre- and post-treatment filter paper weight differences. In cardiac puncture, a penetrating wound was induced on the left ventricular wall. Hydrogel (0.2 ml) was administered to the injury site, with hemostatic time and blood loss quantified as described. All procedures adhered to institutional animal care protocols.

**Coagulation experiment**

An in vitro whole blood coagulation assay was employed to evaluate the hemostatic efficacy of the hydrogel. The coagulation-initiating ability of the hydrogel was assessed using heparinized rat whole blood. Briefly, rats were anesthetized, and 0.5 ml of blood was collected from the retinal venous plexus using a capillary tube and transferred into a centrifuge tube containing heparin. Subsequently, 0.5 ml

of the hydrogel was added to the tube. After allowing the mixture to stand for 1 min, the tube was inverted, and the flow of blood was observed.

**Full-thickness skin wound healing**

A full-thickness skin wound healing study was conducted using male SD rats. After anesthetizing the rats by inhalation of 2% isoflurane for 5 min, an 8 mm diameter full-thickness wound was created on the dorsal region. The rats were randomly assigned to the following groups: a model group (no treatment, $n$ = 6), a 3D-Exo group (0.5 ml, $n$ = 6), a 3D-TE-Exo group (0.5 ml, $n$ = 6), an OLUE hydrogel group (0.5 ml, $n$ = 6), and a positive control group (Tegaderm™, $n$ = 6). In addition, 6 healthy SD rats without any model establishment were included as an independent healthy control group. Wound healing was evaluated by periodically capturing optical images of the wounds and calculating the wound closure rate (%) using ImageJ software. On day 7, the rats were euthanized, and wound tissues were collected for histopathological analysis, including H&E and Masson staining.

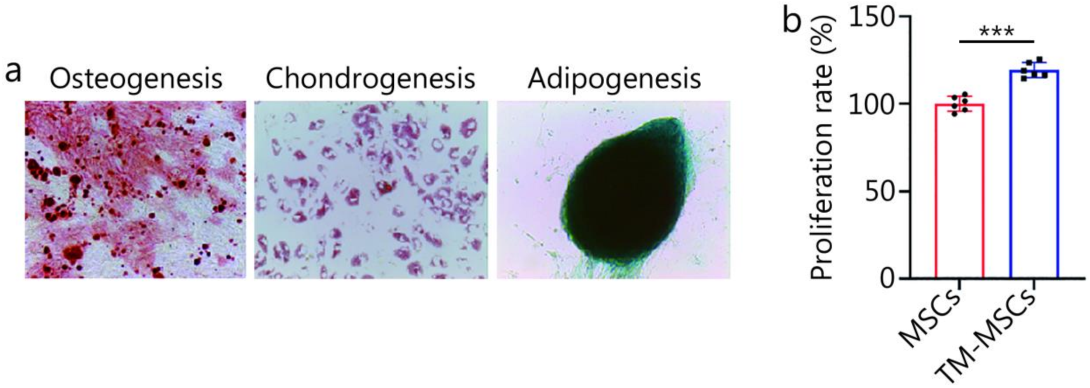


**Fig. S1** Identification and evaluation of MSCs. **a** Tri-lineage differentiation assay of MSCs, including osteogenic, chondrogenic, and adipogenic differentiation. **b** Proliferation rate of MSCs cultured in normal medium and medium containing TE. $^{***}P < 0.001$. MSCs mesenchymal stem cells, TE trace elements

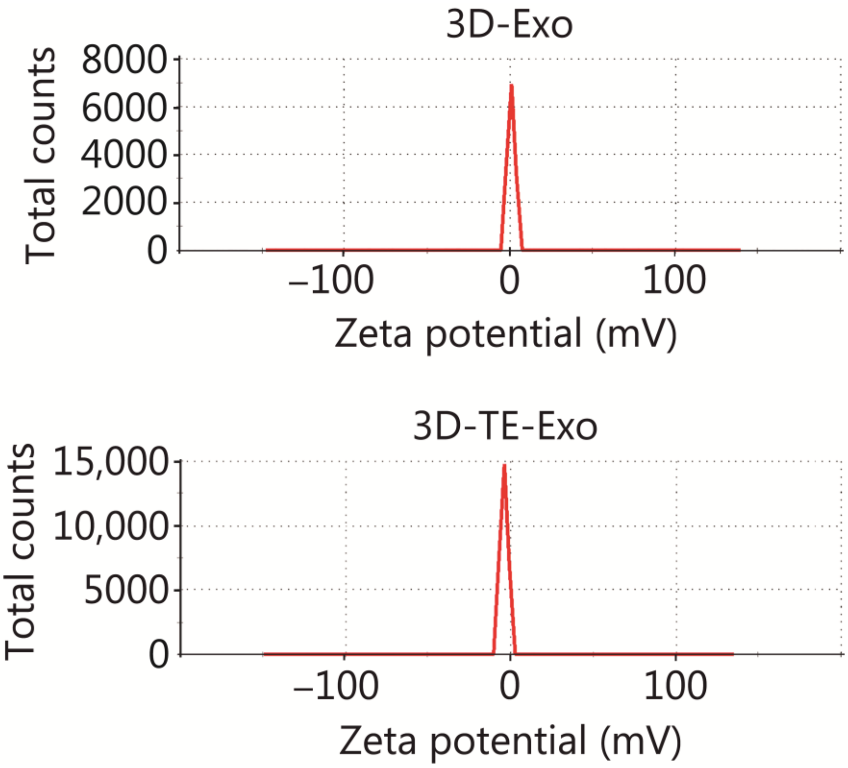


**Fig. S2** Zeta potentials of 3D-Exo and 3D-TE-Exo detected by DLS. 3D-Exo exosome derived from standard medium, 3D-TE-Exo exosome derived from trace element-supplemented medium, DLS dynamic light scattering

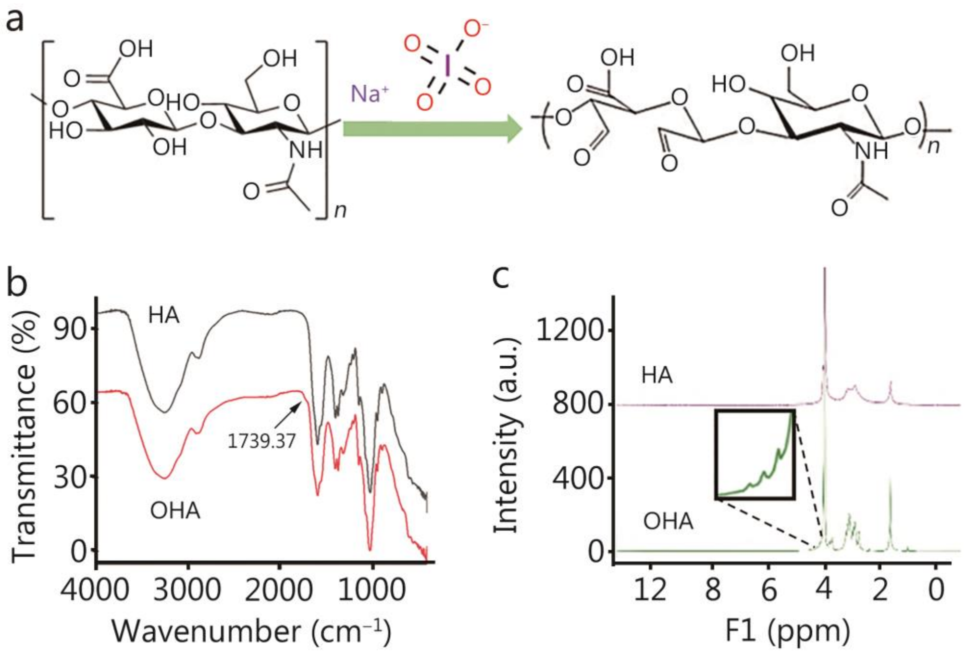


**Fig. S3** Synthesis and identification of OHA. **a** The synthesis equation of OHA. **b** The FT-IR spectra of HA and OHA. **c** The $^1$H-NMR spectrum of HA and OHA. HA hyaluronic acid, OHA oxidized hyaluronic acid, FT-IR fourier transform infrared, $^1$H-NMR nuclear magnetic resonance hydrogen spectroscopy

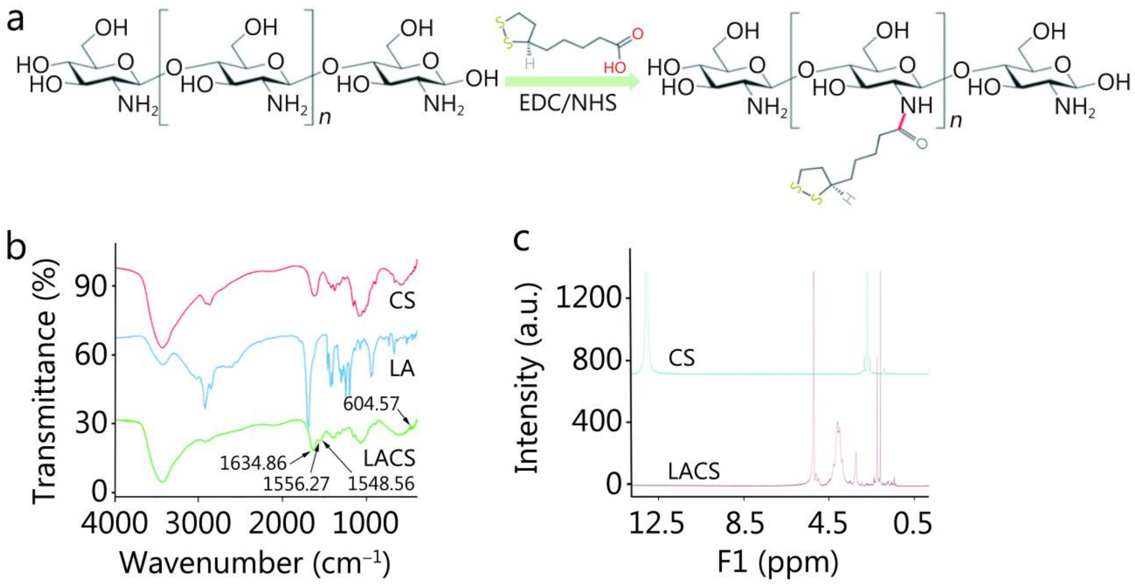


**Fig. S4** Synthesis and identification of LACS. **a** The synthesis equation of LACS. **b** The FT-IR spectra of CS, LA, and LACS. **c** The $^{1}$H-NMR spectrum of CS and LACS. EDC ethyldimethylaminopropyl carbodiimide, NHS N-hydroxysuccinimide, FT-IR fourier transform infrared, CS chitosan, LA lipoic acid, LACS lipoic acid-grafted chitosan, $^{1}$H-NMR nuclear magnetic resonance hydrogen spectroscopy

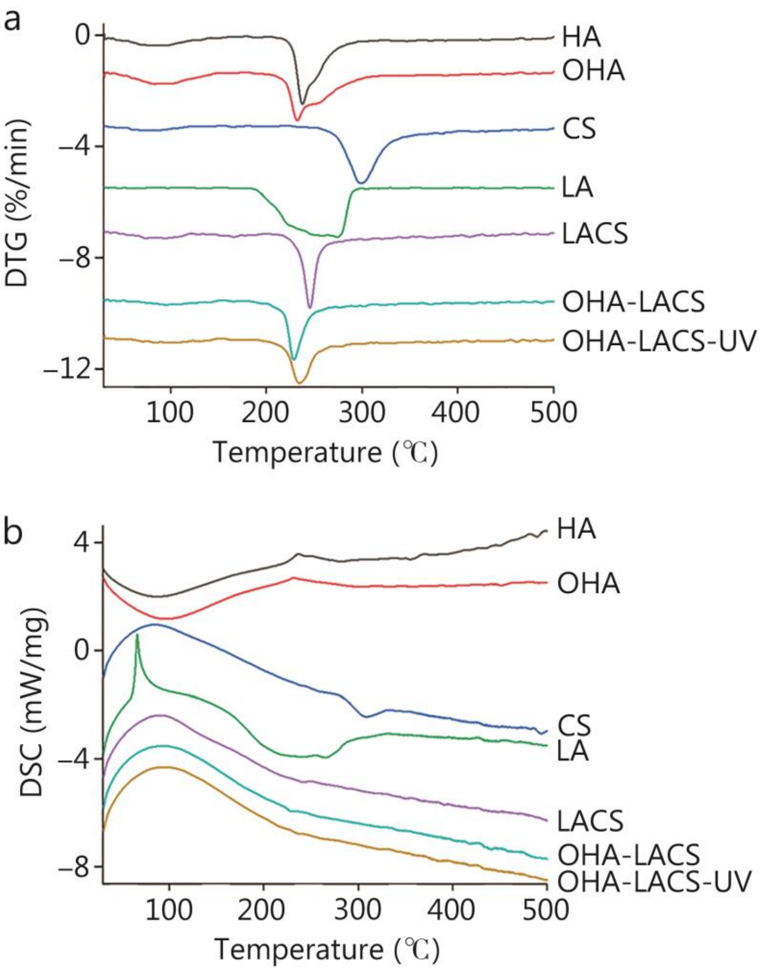


**Fig. S5** Simultaneous thermal analysis of HA, OHA, CS, LA, LACS, OHA-LACS, and OHA-LACS-UV. **a** The DTG spectrum of HA, OHA, CS, LA, LACS, OHA-LACS, and OHA-LACS-UV. **b** The DSC spectrum of HA, OHA, CS, LA, LACS, OHA-LACS, and OHA-LACS-UV. HA hyaluronic acid, OHA oxidized hyaluronic acid, CS chitosan, LA lipoic acid, LACS lipoic acid-grafted chitosan, OHA-LACS hydrogels constructed from oxidized hyaluronic acid and lipoic acid-grafted chitosan, OHA-LACS-UV hydrogels constructed from oxidized hyaluronic acid and lipoic acid-grafted chitosan by ultraviolet light, DTG differential thermogravimetry, DSC differential scanning calorimetry

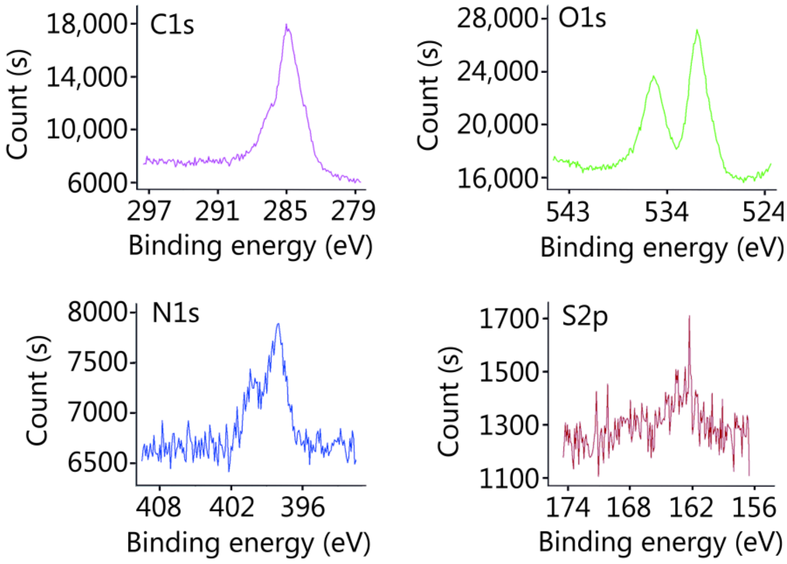


**Fig. S6** The XPS spectra of OHA-LACS hydrogel, including C, O, N, and S. O oxygen, N nitrogen, C carbon, S sulfur, OHA-LACS hydrogels constructed from oxidized hyaluronic acid and lipoic acid-grafted chitosan, XPS X-ray photoelectron spectroscopy

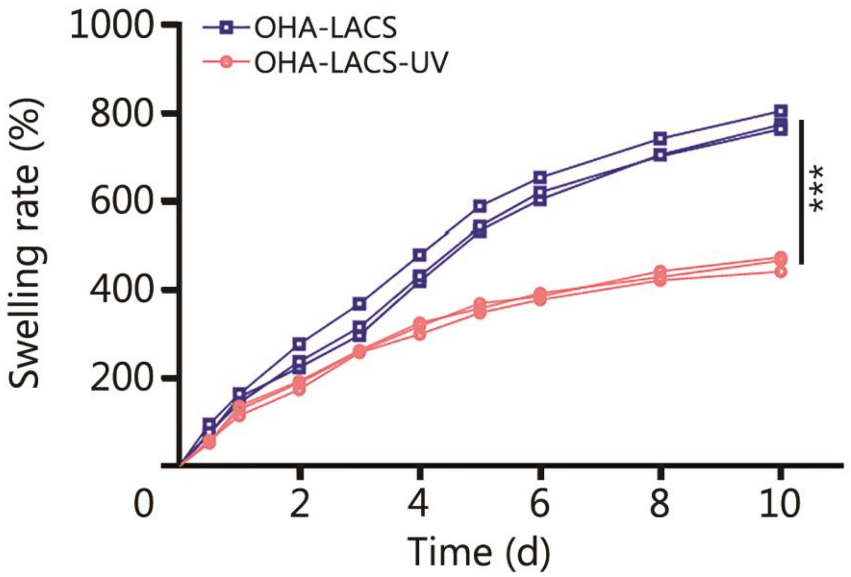


**Fig. S7** The swelling rate of OHA-LACS and OHA-LACS-UV hydrogels ($n$ = 3). $^{***}P < 0.001$. OHA-LACS hydrogels constructed from oxidized hyaluronic acid and lipoic acid-grafted chitosan, OHA-LACS-UV hydrogels constructed from oxidized hyaluronic acid and lipoic acid-grafted chitosan by ultraviolet light

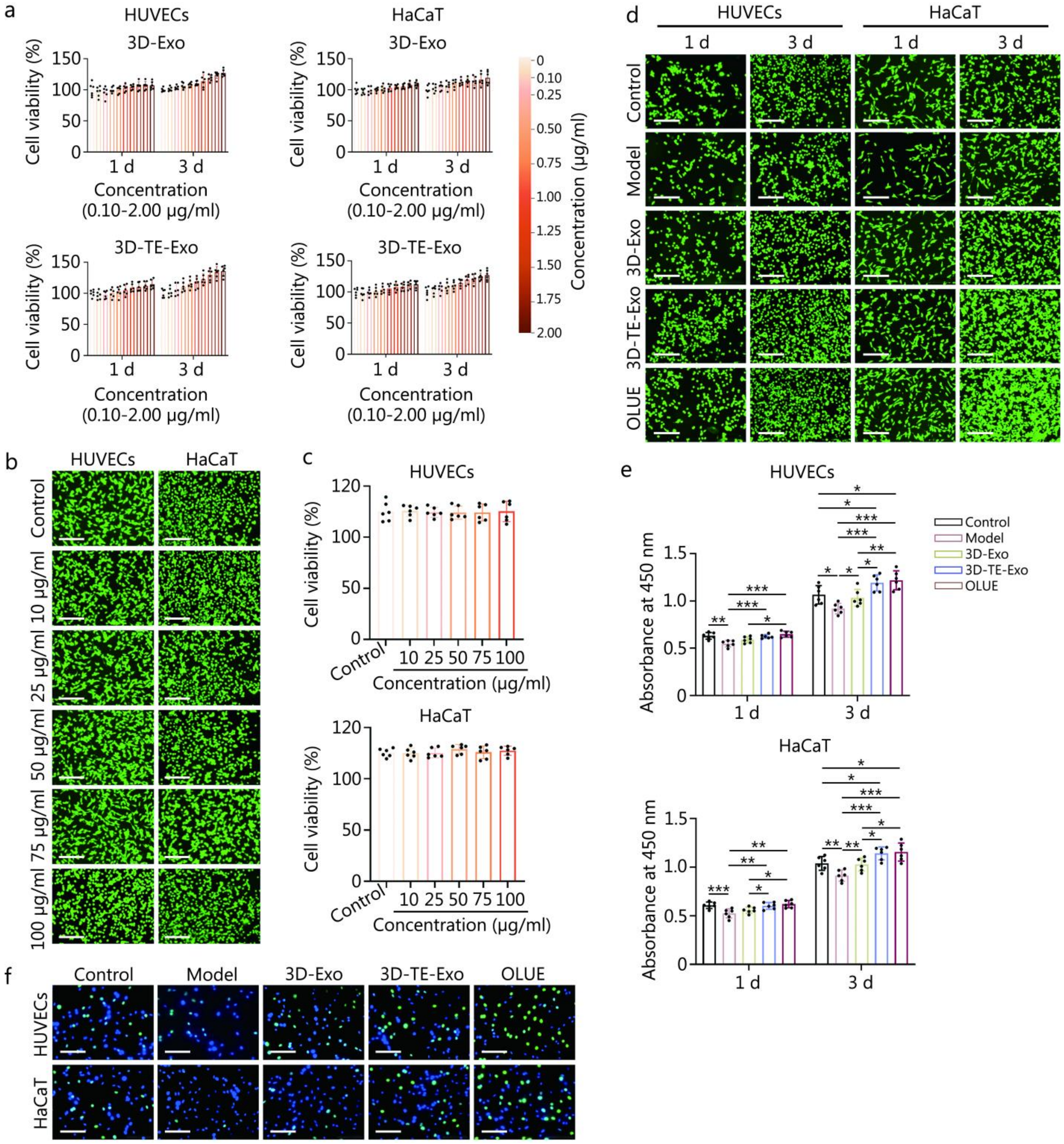


**Fig. S8** Biocompatibility of OLUE hydrogel. **a** Different concentrations of 3D-Exo and 3D-TE-Exo on HUVECs and HaCaT cells (*n* = 6). **b** The Live/dead cell staining of HUVECs and HaCaT incubated with different concentrations of OHA-LACS-UV after 1 and 3 d (scale bar = 200 μm). **c** The CCK-8 results of different concentrations of OHA-LACS-UV on HUVECs and HaCaT cells (*n* = 6). **d** The Live/dead cell staining of HUVECs and HaCaT incubated with different samples after 1 and 3 d (scale bar = 200 μm). **e** The absorbance at 450 nm of HUVECs and HaCaT cells incubated with different samples after 1 and 3 d (*n* = 6). **f** The EdU staining of HUVECs and HaCaT cells incubated with different samples (scale bar = 200 μm). $^{*}P < 0.05$, $^{**}P < 0.01$, $^{***}P < 0.001$. 3D-TE-Exo exosome derived from trace element-supplemented medium, 3D-Exo exosome derived from standard medium, HUVECs human umbilical vein endothelial cells, HaCaT human immortal keratinocyte line, OHA-LACS-UV hydrogels

constructed from oxidized hyaluronic acid and lipoic acid-grafted chitosan by ultraviolet light, CCK-8 cell counting kit-8, OLUE ultraviolet light-irradiated oxidized hyaluronic acid and lipoic acid-grafted chitosan constructed hydrogel for exosomes, EdU 5-ethynyl-2'-deoxyuridine

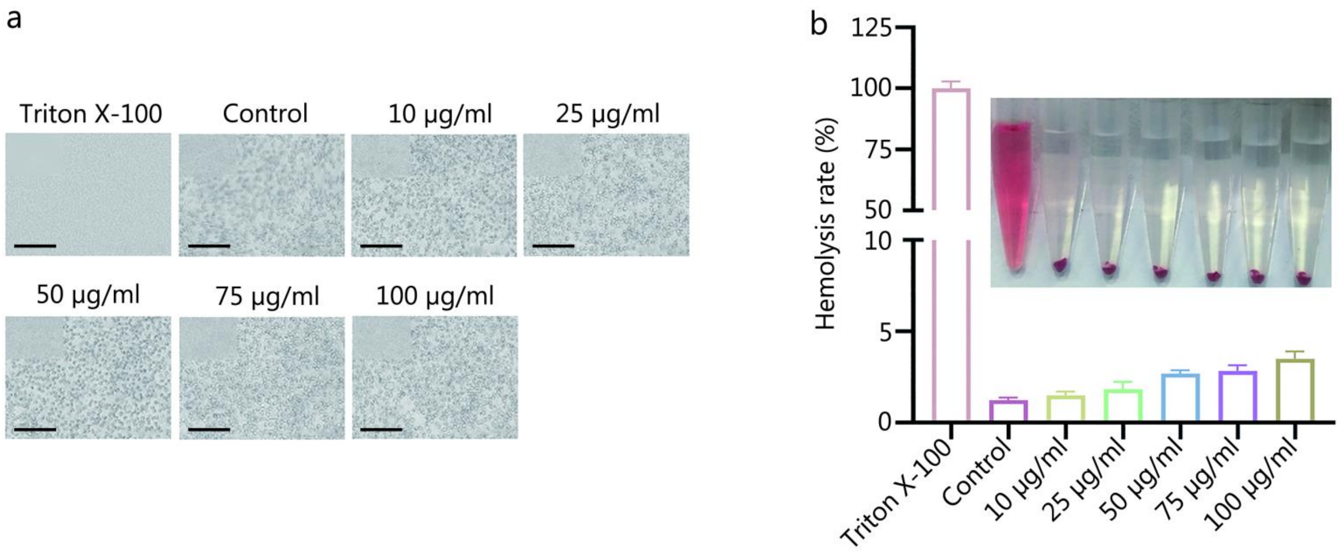


**Fig. S9** Hemolytic properties of OHA-LACS-UV hydrogels. **a** The morphology of red blood cells in different groups (scale bar = 200 μm). **b** Hemolysis rate with red blood cells exposed to OHA-LACS-UV hydrogels (*n* = 3). OHA-LACS-UV hydrogels constructed from oxidized hyaluronic acid and lipoic acid-grafted chitosan by ultraviolet light

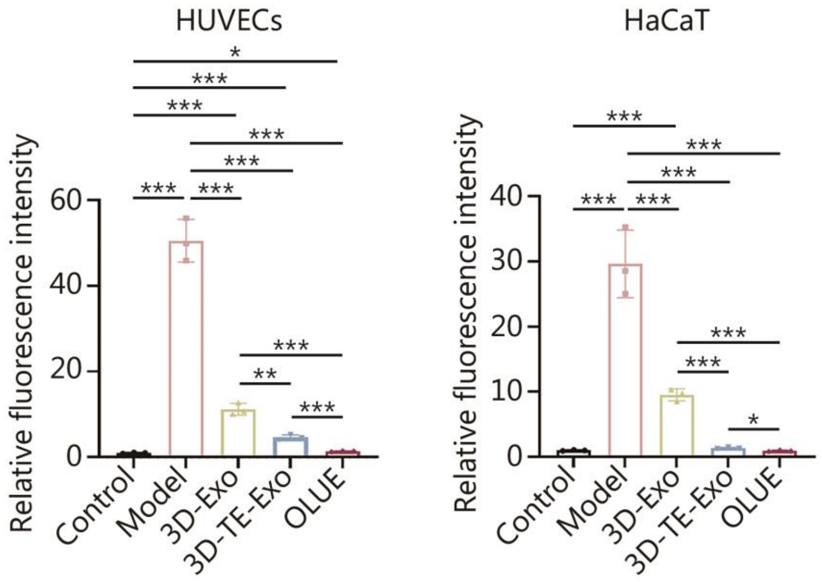


**Fig. S10** Quantification of ROS fluorescence intensity by ImageJ software HUVECs and HaCaT cells ($n$ = 3). $^{*}P$ < 0.05, $^{**}P$ < 0.01, $^{***}P$ < 0.001. ROS reactive oxygen species, HUVECs human umbilical vein endothelial cells, HaCaT human immortal keratinocyte line, 3D-TE-Exo exosome derived from trace element-supplemented medium, 3D-Exo exosome derived from standard medium, OLUE ultraviolet light-irradiated oxidized hyaluronic acid and lipoic acid-grafted chitosan constructed hydrogel for exosomes

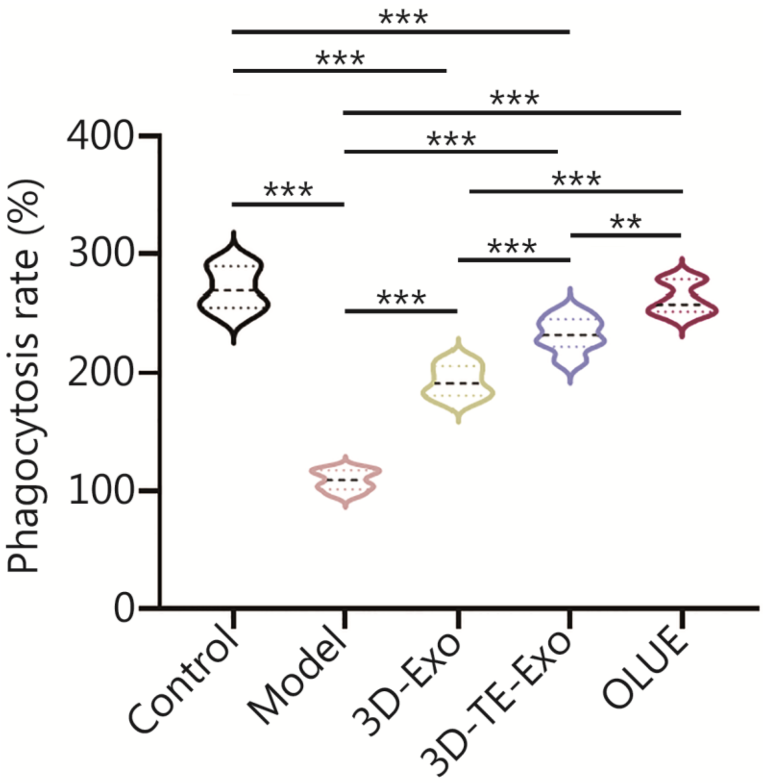


**Fig. S11** Neutral red cell proliferation test ($n$ = 6). $^{**}P < 0.01$, $^{***}P < 0.001$. 3D-TE-Exo exosome derived from trace element-supplemented medium, 3D-Exo exosome derived from standard medium, OLUE ultraviolet light-irradiated oxidized hyaluronic acid and lipoic acid-grafted chitosan constructed hydrogel for exosomes

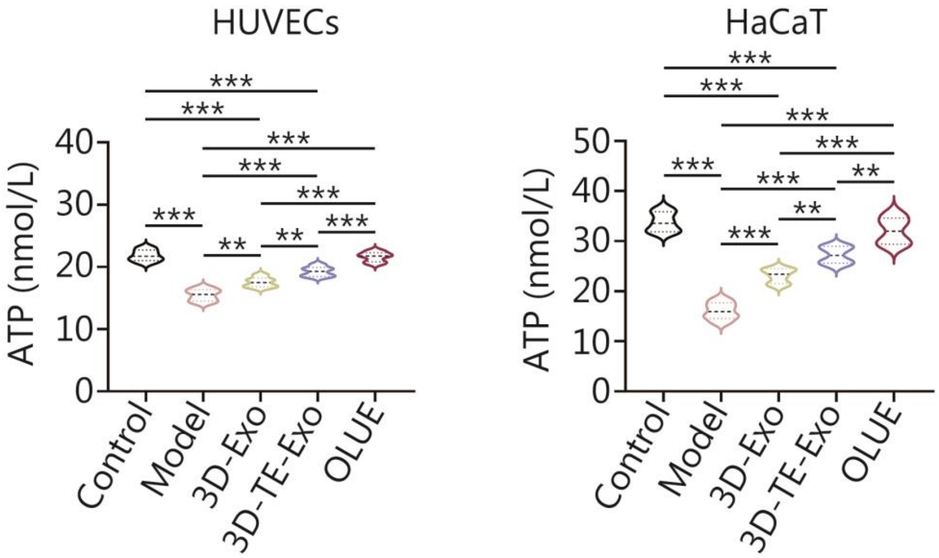


**Fig. S12** ATP in the mitochondria of HUVECs and HaCaT cells ($n = 6$). $^{**}P < 0.01$, $^{***}P < 0.001$. 3D-TE-Exo exosome derived from trace element-supplemented medium, 3D-Exo exosome derived from standard medium, OLUE ultraviolet light-irradiated oxidized hyaluronic acid and lipoic acid-grafted chitosan constructed hydrogel for exosomes, ATP adenosine triphosphate

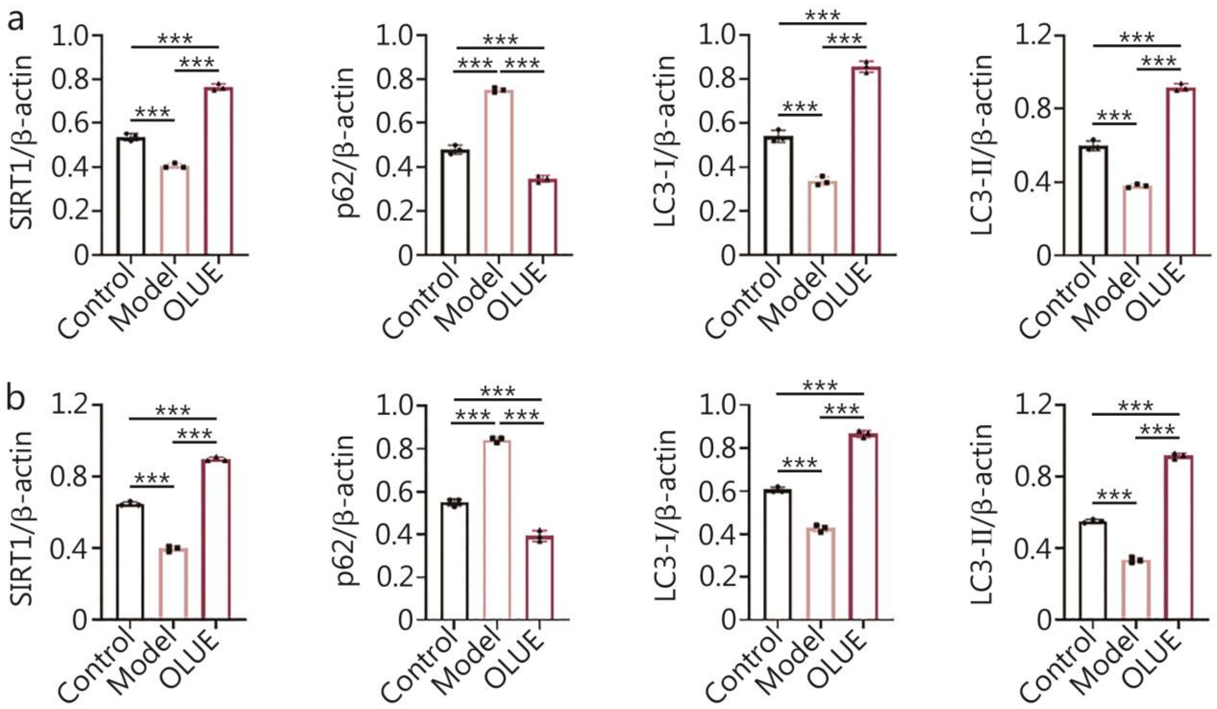


**Fig. S13** Quantification of Western blotting results. **a** Effects of SIRT1, p62, LC3-I, and LC3-II on HUVECs ($n$ = 3). **b** Effects of SIRT1, p62, LC3-I, and LC3-II on HaCaT cells ($n$ = 3). $^{***}P < 0.001$. 3D-TE-Exo exosome derived from trace element-supplemented medium, 3D-Exo exosome derived from standard medium, OLUE ultraviolet light-irradiated oxidized hyaluronic acid and lipoic acid-grafted chitosan constructed hydrogel for exosomes, SIRT1 silent information regulator 1, p62 sequestosome 1, LC3 microtubule-associated protein light chain 3, HUVECs human umbilical vein endothelial cells, HaCaT human immortal keratinocyte line

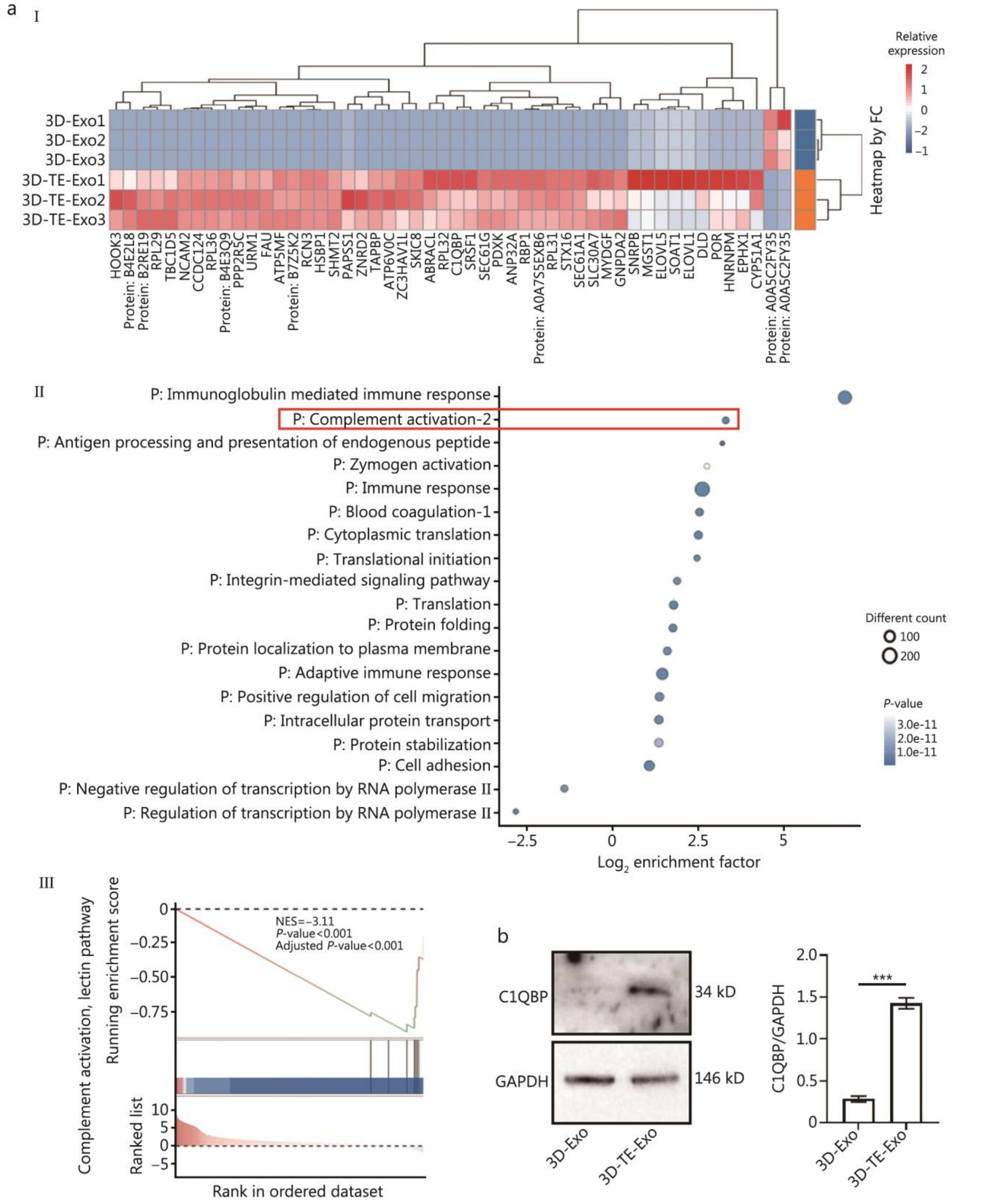


**Fig. S14** Expression and identification of C1QBP in 3D-TE-Exo. **a** Proteomics. Differential protein heatmap (i), Gene Ontology (GO) enrichment bubble chart (ii), and Gene Set Enrichment Analysis for GO (iii) (3D-Exo vs. 3D-TE-Exo). **b** Expression of C1QBP protein in 3D-Exo and 3D-TE-Exo ($n$ = 3). $^{***}P < 0.001$. 3D-Exo exosome derived from standard medium, 3D-TE-Exo exosome derived from trace element-supplemented medium, CYP51A1 cytochrome P450 family 51 subfamily a member 1, EPHX1 epoxide hydrolase 1, HNRNPM heterogeneous nuclear

ribonucleoprotein m, POR cytochrome P450 oxidoreductase, DLD dihydrolipoamide dehydrogenase, ELOVL1 elongation of very long chain fatty acids protein 1, SOAT1 sterol o-acyltransferase 1, ELOVL5 elongation of very long chain fatty acids protein 5, MGST1 microsomal glutathione s-transferase 1, SNRPB small nuclear ribonucleoprotein polypeptide b, GNPDA2 glucosamine-6-phosphate deaminase 2, MYDGF myeloid-derived growth factor, SLC30A7 solute carrier family 30 member 7, SEC61A1 SEC61 translocon subunit alpha 1, STX16 syntaxin 16, RPL31 ribosomal protein L31, RBP1 retinol binding protein 1, ANP32A acidic nuclear phosphoprotein 32 family member a, PDXK pyridoxal kinase, SEC61G SEC61 translocon subunit gamma, SRSF1 serine/arginine-rich splicing factor 1, C1QBP complement C1q binding protein, RPL32 ribosomal protein L32, ABRACL ABRA C-terminal like, SKIC8 SKI8 homolog, ZC3HAV1L zinc finger CCCH-type containing antiviral 1 like, ATP6V0C ATPase $H^+$ transporting v0 subunit C, TAPBP TAP binding protein, ZNRD2 zinc ribbon domain containing 2, PAPSS1 3'-phosphoadenosine 5'-phosphosulfate synthase 1, SHMT2 serine hydroxymethyltransferase 2, HSBP1 heat shock factor binding protein 1, RCN3 reticulocalbin 3, ATP5MF ATP synthase membrane subunit f, FAU finkel-biskis-reilly murine sarcoma virus ubiquitously expressed, URM1 ubiquitin related modifier 1, PPP2R5C protein phosphatase 2 regulatory subunit B' gamma, RPL36 60S ribosomal protein L36, CCDC124 coiled-coil domain containing 124, NCAM2 neural cell adhesion molecule 2, TBC1D5 TBC1 domain family member 5, RPL29 ribosomal protein L29, HOOK3 hook microtubule-tethering protein 3, GAPDH glyceraldehyde-3-phosphate dehydrogenase, NES normalized enrichment score

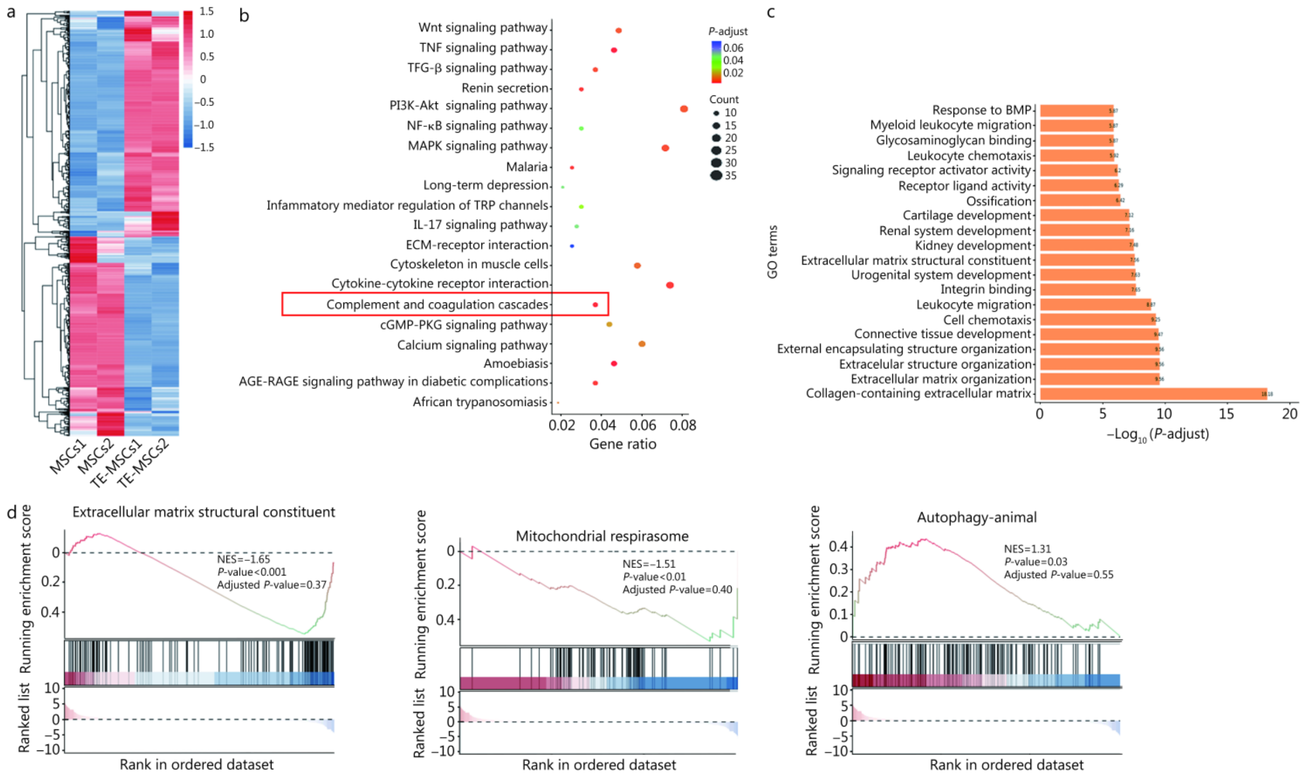


**Fig. S15** Expression and identification of C1QBP in MSCs and TE-MSCs. **a** Gene expression heatmap of MSCs and TE-MSCs. **b** Gene Ontology (GO) enrichment bar plot of MSCs and TE-MSCs. **c** Plot KEGG enrichment of MSCs and TE-MSCs. **d** Gene Set Enrichment Analysis for GO of MSCs and TE-MSCs. MSCs mesenchymal stem cells, TE-MSCs mesenchymal stem cells regulated by trace elements, KEGG Kyoto Encyclopedia of Genes and Genomes, C1QBP complement C1q binding protein, WNT wingless-type MMTV integration site family, TNF tumor necrosis factor, TGF transforming growth factor, PI3K/Akt phosphatidylinositol 3-kinase/Ak strain transforming, NF-κB nuclear factor kappa-light-chain-enhancer of activated B cells, MAPK mitogen-activated protein kinase, TRP transient receptor potential, IL-17 interleukin-17, ECM extracellular matrix, cGMP-PKG cyclic guanosine monophosphate-protein kinase G, AGE-RAGE advanced glycation end products-receptor for advanced glycation end products, BMP bone morphogenetic protein, NES normalized enrichment score

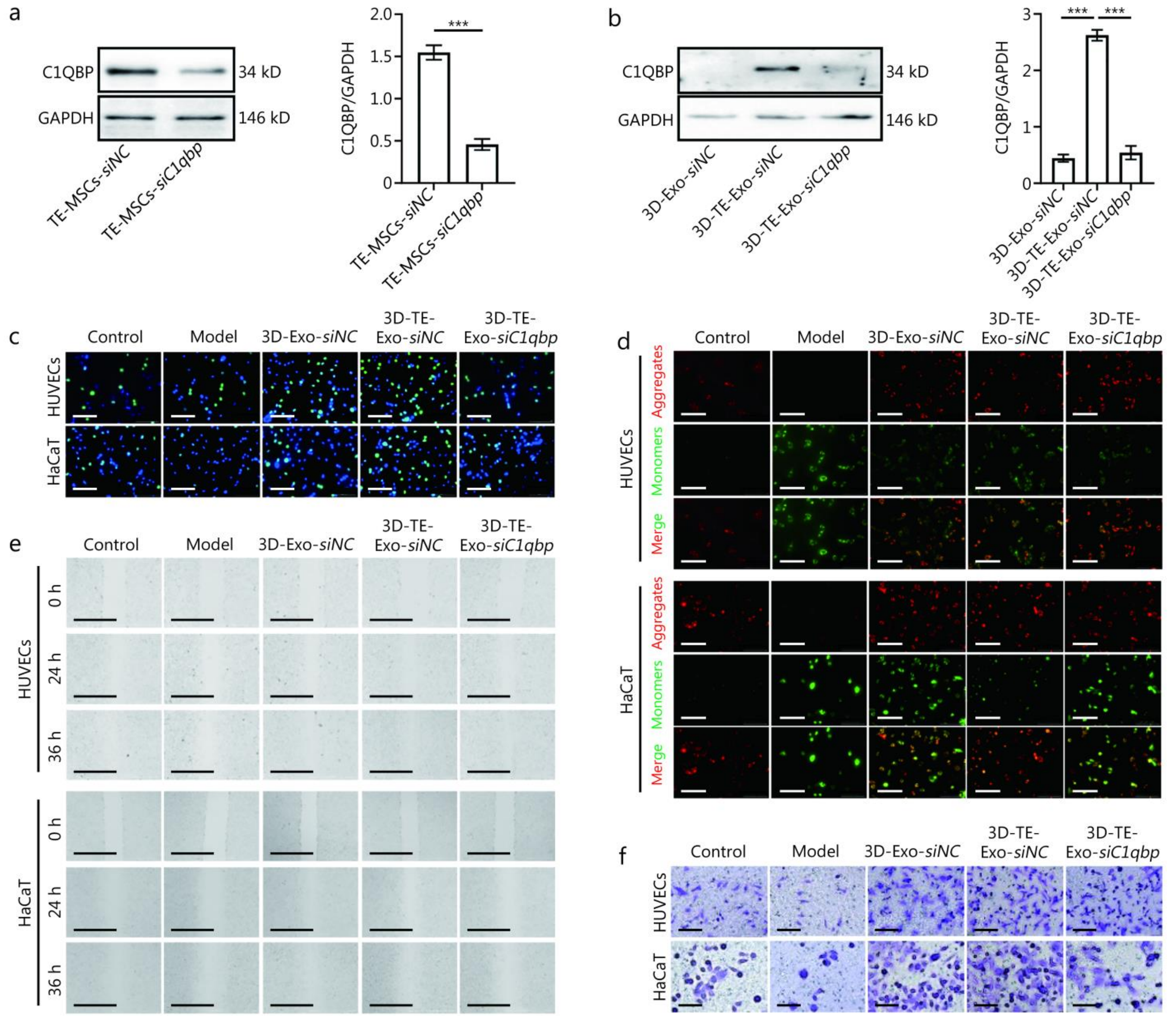


**Fig. S16** Silence and functional evaluation of C1QBP. **a** Expression of C1QBP protein in TE-MSCs silenced by siRNA *C1pbp*. **b** C1QPBP protein levels in 3D-TE-Exo secreted by TE-MSCs with *C1qbp* gene knocked out. **c** The EdU staining of control, model, 3D-Exo-*siNC,* 3D-TE-Exo-*siNC*, and 3D-TE-Exo-*siC1qbp* (scale bar = 200 μm). **d** Representative plot of mitochondrial membrane potential shown by JC-1 staining of HUVECs or HaCaT cells cultured with si-3D-TE-Exo (scale bar = 20 μm). **e** Cell migration of HUVECs or HaCaT cells in different groups at 0, 24, and 36 h (scale bar = 1000 μm). **f** Representative micrographs showing the effect of 3D-TE-Exo-*siC1qbp* on Transwell migration of HUVECs or HaCaT cells (scale bar = 200 μm). $^{***}P < 0.001$. C1QBP complement c1q binding protein, MSCs mesenchymal stem cells, EdU 5-ethynyl-2'-deoxyuridine, JC-1 5,5′,6,6′-tetrachloro-1,1′,3,3′-tetraethyl-imidacarbocyanine iodide, HUVECs human umbilical vein endothelial cells, HaCaT human immortal keratinocyte line, GAPDH glyceraldehyde-3-phosphate dehydrogenase

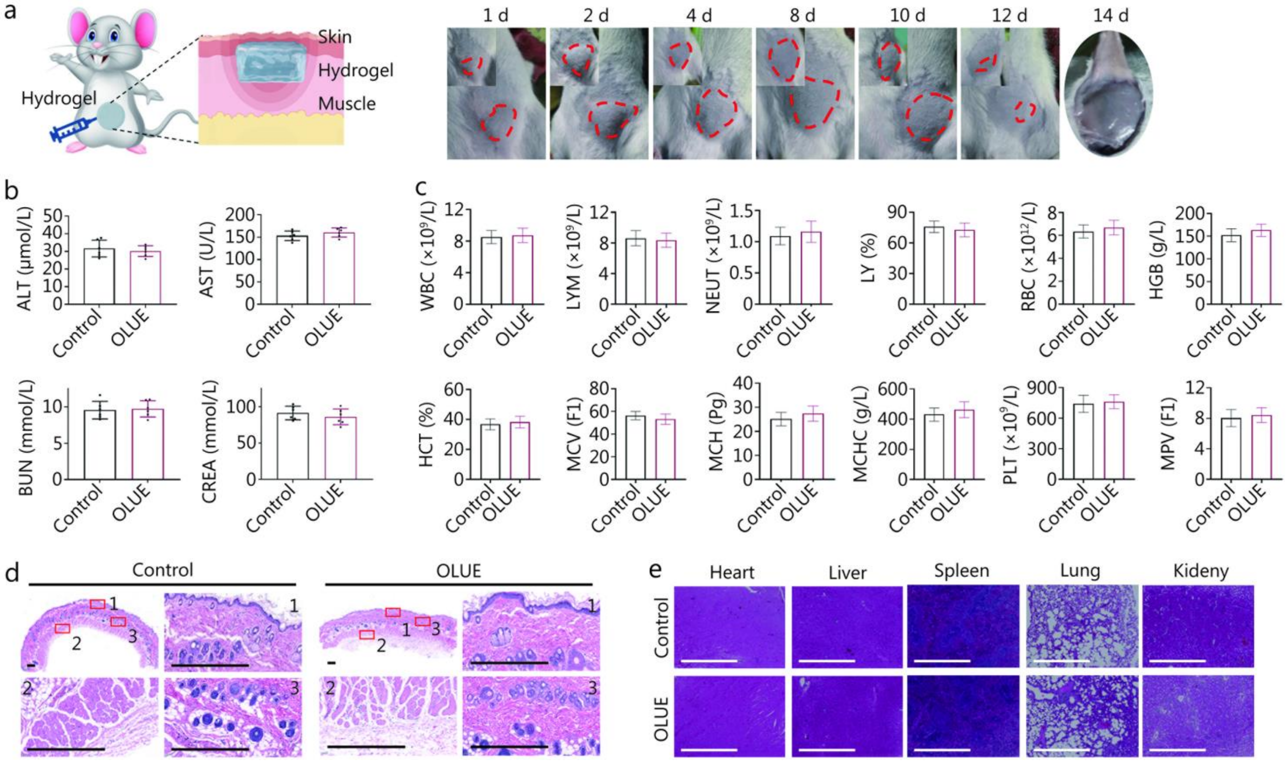


**Fig. S17** In vivo retention and safety properties of OLUE hydrogel. **a** Representative photo of OLUE hydrogel retained subcutaneously. **b** Blood biochemical of rats with OLUE hydrogel retained in vivo for 14 d, including ALT, AST, BUN, and CREA ($n = 6$). **c** Routine blood indexes of rats of control and OLUE groups ($n = 6$). **d** H&E staining of the skin at the OLUE hydrogel retention site at 14 d: epidermal layer (1), dermis layer (2), and subcutaneous tissue (3) (scale bar = 500 μm). **e** H&E staining of rats of control and OLUE groups (scale bar = 1000 μm). OLUE ultraviolet light-irradiated oxidized hyaluronic acid and lipoic acid-grafted chitosan constructed hydrogel for exosomes, ALT alanine aminotransferase, AST aspartate aminotransferase, BUN blood urea nitrogen, CREA creatinine, WBC white blood cell count, LYM lymphocytes, NEUT neutrophils, LY% lymphocyte proportion, RBC red blood cells, HGB hemoglobin, HCT hematocrit, MCV mean corpuscular volume, MCH mean corpuscular hemoglobin, MCHC mean corpuscular hemoglobin concentration, PLT platelet count, MPV mean platelet volume

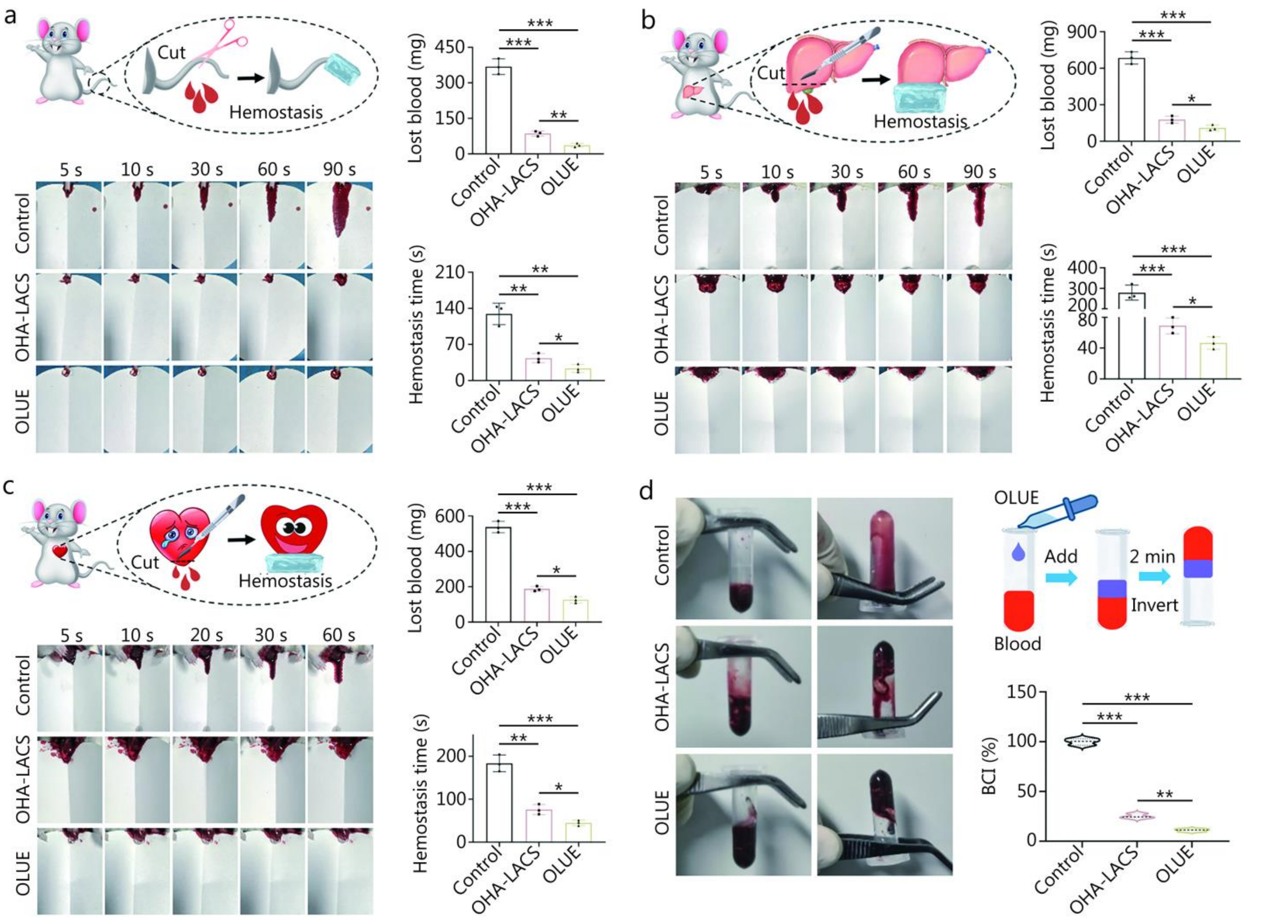


**Fig. S18** In vivo hemostasis and coagulation properties of OLUE hydrogel. **a** The hemostatic effect of OLUE in docking model, including schematic diagram, representative photos, loss blood, and hemostatic time ($n$ = 3). **b** The hemostatic effect of OLUE in liver injury model, including schematic diagram, representative photos, loss blood, and hemostatic time ($n$ = 3). **c** The hemostatic effect of OLUE in heart injury model, including schematic diagram, representative photos, loss blood, and hemostatic time ($n$ = 3). **d** In vitro coagulation effect of hydrogel, including representative pictures, schematics, and BCI ($n$ = 3). $^{*}P < 0.05$, $^{**}P < 0.01$, $^{***}P < 0.001$. OLUE ultraviolet light-irradiated oxidized hyaluronic acid and lipoic acid-grafted chitosan constructed hydrogel for exosomes, OHA-LACS hydrogels constructed from oxidized hyaluronic acid and lipoic acid-grafted chitosan, BCI blood clotting index

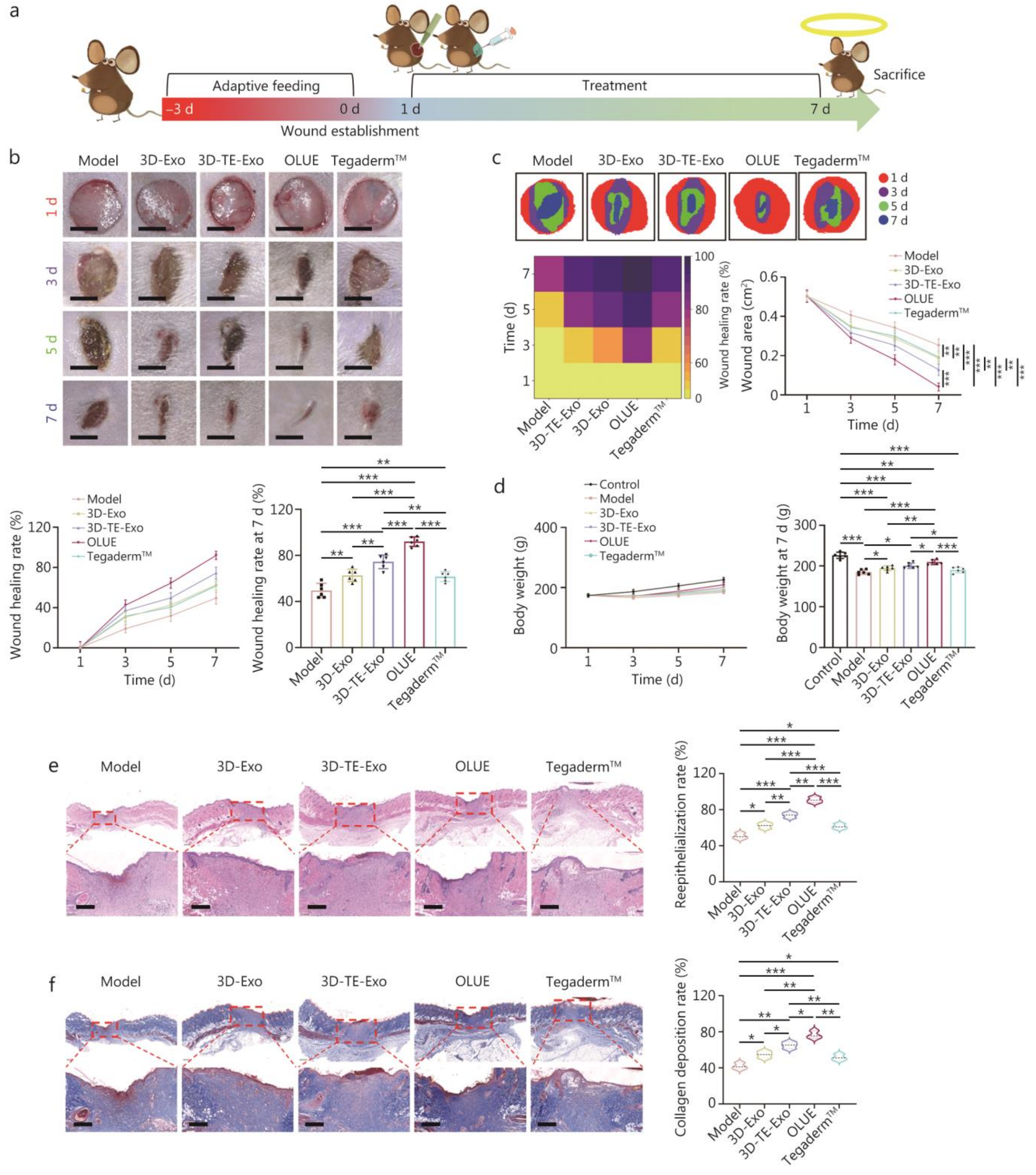


**Fig. S19** In vivo evaluation of OLUE hydrogel in the healthy rats full-thickness wound. **a** Schematic diagram of the establishment and treatment of full-thickness wound model. **b** Macroscopic images (scale bar = 0.5 cm) and wound healing rate ($n$ = 6) at 1, 3, 5, and 7 days of each group. **c** The contour map and change ($n$ = 6) of the wound healing process in each group. **d** Body weight changes in rats at 1, 3, 5, and 7 d ($n$ = 6). **e** Representative images of H&E staining (scale bar = 400 μm) and quantification of reepithelialization rate (%) ($n$ = 3) of wound for different groups at 7 d. **f** Representative images of Masson staining (scale bar = 400 μm) and quantification of collagen deposition rate ($n$ = 3) of different treatments after treatment for 7 d. $^{*}P < 0.05$, $^{**}P < 0.01$, $^{***}P < 0.001$. 3D-TE-Exo exosome

derived from trace element-supplemented medium, 3D-Exo exosome derived from standard medium, OLUE ultraviolet light-irradiated oxidized hyaluronic acid and lipoic acid-grafted chitosan constructed hydrogel for exosomes, H&E staining hematoxylin-eosin staining

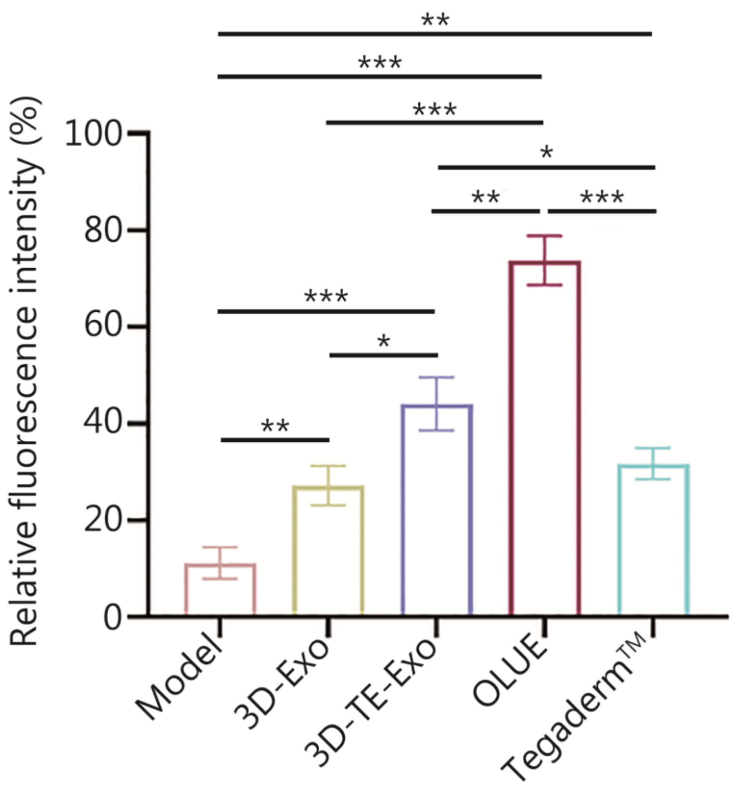


**Fig. S20** Quantification of α-SMA fluorescence intensity by ImageJ software ($n$ = 3). $^{*}P < 0.05$, $^{**}P < 0.01$, $^{***}P < 0.001$. α-SMA α-smooth muscle actin, 3D-TE-Exo exosome derived from trace element-supplemented medium, 3D-Exo exosome derived from standard medium, OLUE ultraviolet light-irradiated oxidized hyaluronic acid and lipoic acid-grafted chitosan constructed hydrogel for exosomes

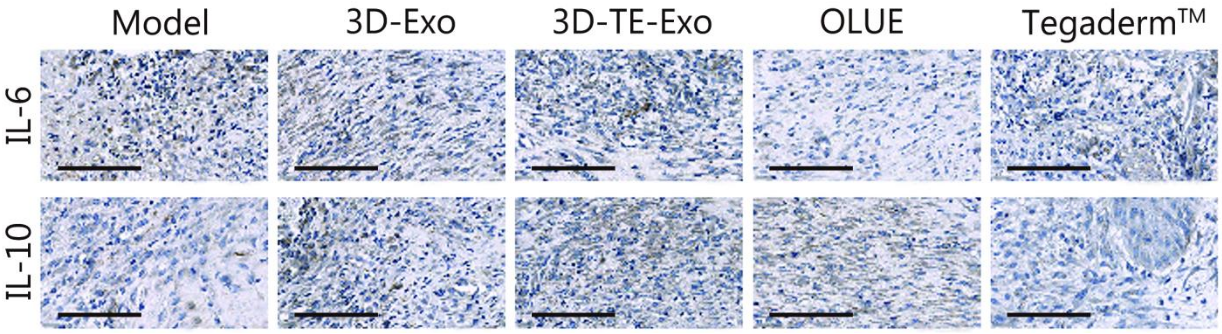


**Fig. S21** Immunohistochemistry staining for IL-6 and IL-10 of representative wound tissues after 14 days of treatment (scale bar = 100 μm). IL-6 interleukin-6, IL-10 interleukin-10, 3D-Exo exosome derived from standard medium, 3D-TE-Exo exosome derived from trace element-supplemented medium, OLUE ultraviolet light-irradiated oxidized hyaluronic acid and lipoic acid-grafted chitosan constructed hydrogel for exosomes

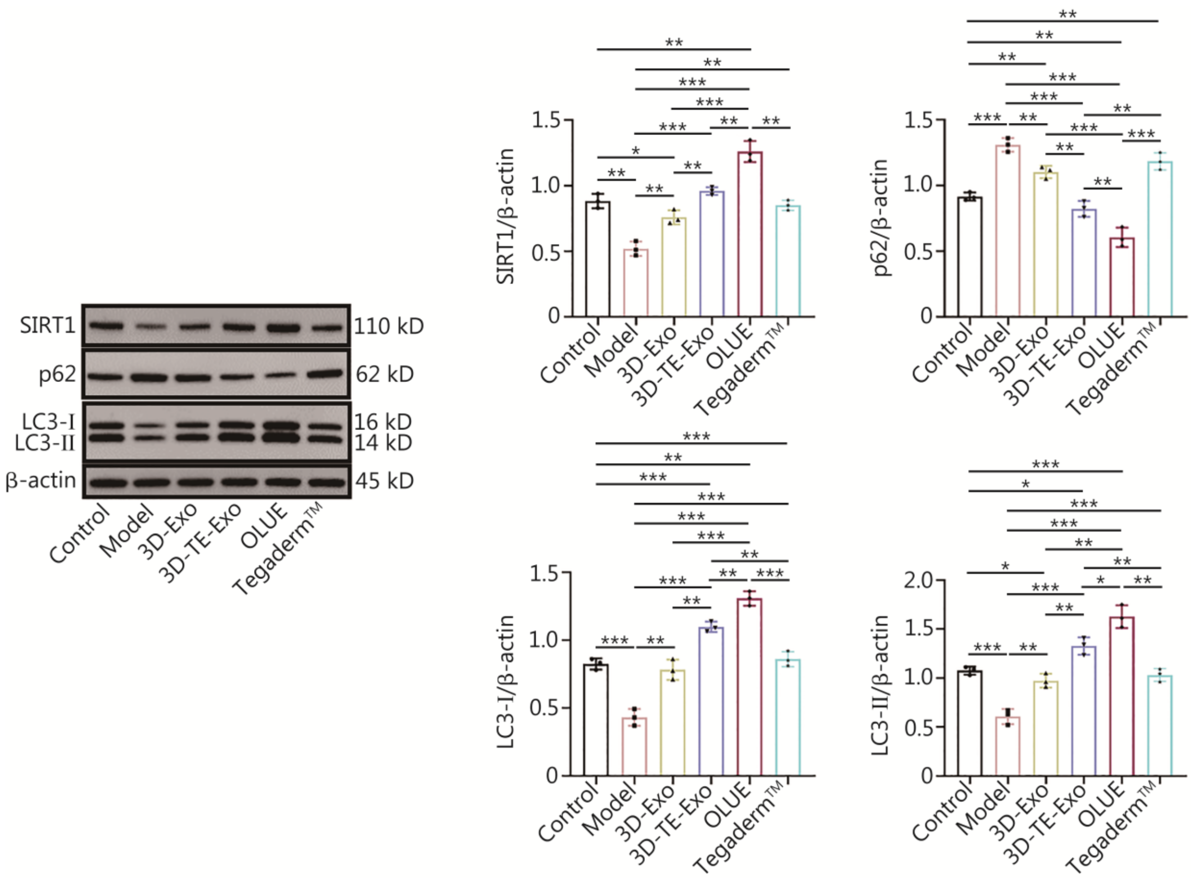


**Fig. S22** Expression of SIRT1 and autophagy pathway proteins in diabetic wound tissues. **a** Western blotting and the quantitative analysis of SIRT1, p62, LC3-I, and LC3-II ($n = 3$). $^{*}P < 0.05$, $^{**}P < 0.01$, $^{***}P < 0.001$. SIRT1 silent information regulator 1, p62 sequestosome 1, LC3 microtubule-associated protein light chain 3, 3D-TE-Exo exosome derived from trace element-supplemented medium, 3D-Exo exosome derived from standard medium, OLUE ultraviolet light-irradiated oxidized hyaluronic acid and lipoic acid-grafted chitosan constructed hydrogel for exosomes

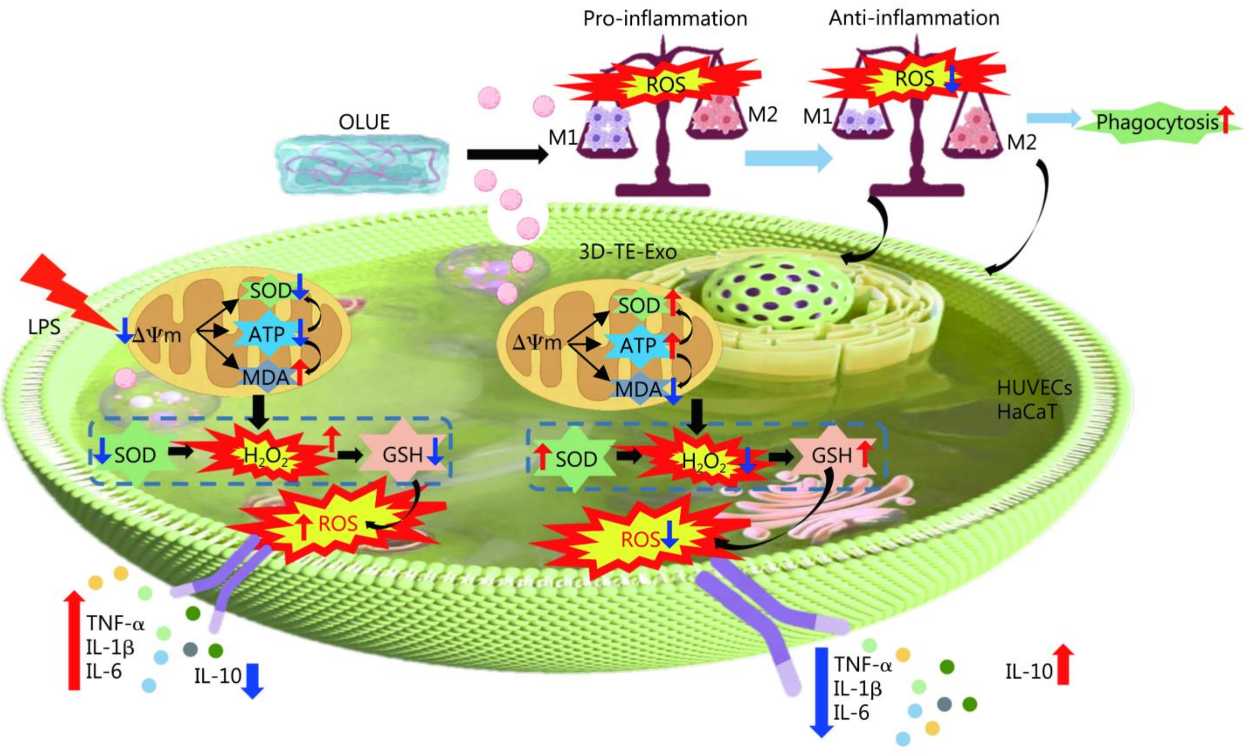


**Fig. S23** Schematic diagram of anti-inflammatory and antioxidant properties of OLUE hydrogel. $H_2O_2$ hydrogen peroxide, HUVECs human umbilical vein endothelial cells, HaCaT human immortal keratinocyte line, MDA malondialdehyde, SOD superoxide dismutase, GSH glutathione, TNF-α tumour necrosis factor-alpha, IL-1β interleukin-1β, IL-6 interleukin-6, IL-10 interleukin-10, ATP adenosine triphosphate, ROS reactive oxygen species, 3D-TE-Exo exosome derived from trace element-supplemented medium, OLUE ultraviolet light-irradiated oxidized hyaluronic acid and lipoic acid-grafted chitosan constructed hydrogel for exosomes, ΔΨm mitochondrial membrane potential, LPS lipopolysaccharide

**Table S1** Primer sequences used for RT-PCR

| Gene | Sequences (5' – 3') |
|---|---|
| *Tnf-α* | Forward: GCTCCCTCTCATCAGTTCCA |
| | Reverse: GCTTGGTGGTTTGCTACGAC |
| *Il-1β* | Forward: TCTGAAGCAGCTATGGCAAC |
| | Reverse: TCAGCCTCAAAGAACAGGTCA |
| *Arg-1* | Forward: TTGATGTTGATGGACTGGA |
| | Reverse: CTCTGGCTTATGATTACCTTC |
| *Il-10* | Forward: ATGCTGCCTGCTCTTACTGACTG |
| | Reverse: CCCAAGTAACCCTTAAAGTCCTGC |
| *GAPDH* | Forward: AGTGCCAGCCTCGTCTCATA |
| | Reverse: TGAACTTGCCGTGGGTAGAG |

*Tnf-α* tumor necrosis factor-α, *Il-1β* interleukin-1β, *Arg-1* arginase-1, *IL-10* interleukin-10, *GAPDH* glyceraldehyde-3-phosphate dehydrogenase, *RT-PCR* reverse transcription-polymerase chain reaction

**Table S2** Trace element species and concentrations in cell culture medium

| **Element types** | **Concentration (μg/L)** |
|---|---|
| Fe | 600 |
| Mg | 559.37 |
| Zn | 189.38 |
| Mn | 48.13 |
| Se | 6.59 |

**Table S3** Mathematical models of the regression for in vitro release profiles of preparations ($R^2$)

| **Dissolution conditions** | **Zero order** | **First order** | **Korsmeyer-Peppas** | **Huguchi** | **Weibull** |
|---|---|---|---|---|---|
| pH 7.4 PBS | 0.9748 | 0.9994 | 0.9965 | 0.8082 | 0.8521 |
| 1 mmol/L $H_2O_2$ | 0.9431 | 0.9940 | 0.9803 | 0.9601 | 0.9987 |
| 3 mg/ml Glu | 0.9888 | 0.9885 | 0.9897 | 0.9062 | 0.9916 |
| 1 mmol/L $H_2O_2$ + 3 mg/ml Glu | 0.8154 | 0.9845 | 0.9410 | 0.9417 | 0.9984 |

*PBS* phosphate buffered saline, $H_2O_2$ hydrogen peroxide, $R^2$ Regression coefficient, *Glu* glucose
